# *Bacopa monnieri* and Their Bioactive Compounds Inferred Multi-Target Treatment Strategy for Neurological Diseases: A Cheminformatics and System Pharmacology Approach

**DOI:** 10.3390/biom10040536

**Published:** 2020-04-02

**Authors:** Rajendran Jeyasri, Pandiyan Muthuramalingam, Vellaichami Suba, Manikandan Ramesh, Jen-Tsung Chen

**Affiliations:** 1 Department of Biotechnology, Science Campus, Alagappa University, Karaikudi 630 003, India; jeyasri8220@gmail.com (R.J.); pandianmuthuramalingam@gmail.com (P.M.); subahrj@gmail.com (V.S.); 2 Department of Life Sciences, National University of Kaohsiung, Kaohsiung 811, Taiwan; jentsung@nuk.edu.tw

**Keywords:** Alzheimer’s disease, *Bacopa monnieri*, bioactive compounds, cheminformatics, neurological diseases, spinocerebellar ataxia, system pharmacology

## Abstract

Neurological diseases (NDs), especially Alzheimer’s and Spinocerebellar ataxia (SCA), can severely cause biochemical abnormalities in the brain, spinal cord and other nerves of human beings. Their ever-increasing prevalence has led to a demand for new drug development. Indian traditional and Ayurvedic medicine used to combat the complex diseases from a holistic and integrative point of view has shown efficiency and effectiveness in the treatment of NDs. *Bacopa monnieri* is a potent Indian medicinal herb used for multiple ailments, but is significantly known as a nootropic or brain tonic and memory enhancer. This annual herb has various active compounds and acts as an alternative and complementary medicine in various countries. However, system-level insights of the molecular mechanism of a multiscale treatment strategy for NDs is still a bottleneck. Considering its prominence, we used cheminformatics and system pharmacological approaches, with the aim to unravel the various molecular mechanisms represented by Bacopa-derived compounds in identifying the active human targets when treating NDs. First, using cheminformatics analysis combined with the drug target mining process, 52 active compounds and their corresponding 780 direct receptors were retrieved and computationally validated. Based on the molecular properties, bioactive scores and comparative analysis with commercially available drugs, novel and active compounds such as asiatic acid (ASTA) and loliolide (LLD) to treat the Alzheimer’s and SCA were identified. According to the interactions among the active compounds, the targets and diseases were further analyzed to decipher the deeper pharmacological actions of the drug. NDs consist of complex regulatory modules that are integrated to dissect the therapeutic effects of compounds derived from Bacopa in various pathological features and their encoding biological processes. All these revealed that Bacopa compounds have several curative activities in regulating the various biological processes of NDs and also pave the way for the treatment of various diseases in modern medicine.

## 1. Introduction

Medicinal plants are a pivotal reservoir of plenty of pharmacologically active compounds, and have been used as a therapeutic medicine for several diseases since the ancient period. Medicinal plants are the essential backbone of traditional medicine; around 3.3 billion people in the least-developed countries are still utilizing them on a regular basis [1]. Medicinal plants offer rich resources of pharmacological ingredients that can be used in drug development. Above and beyond that, these plants play a significant role in the development of human cultures around the globe. According to the International Union for Conservation of Nature (IUCN) report, worldwide, almost 80,000 flowering plant species are used for pharmaceutical purposes.

*Bacopa monnieri* (L.) is an important medicinal plant in Indian traditional Ayurvedic medicines. It is a small perennial herbaceous plant commonly known as ‘Brahmi’, belonging to the family Scrophulariaceae. It is a renowned Indian medicinal plant that has been used as a memory booster in the Ayurvedic medicinal system for more than 3000 years [2,3]. It is used in traditional medicine to treat various nervous disorders, digestive aid, improve learning, memory, and concentration and to provide relief to patients with anxiety, and skin disorders; specific uses include the treatment of asthma, insanity and epilepsy [4,5,6]. The Bacopa herb, also called nootropic herb, helps in the repair of damaged neurons, neuronal synthesis, and the restoration of synaptic activity, and improves brain function. *B. monnieri* contains alkaloid brahmine, nicotinine, herpestine, bacosides A and B, saponins A, B and C, triterpenoid saponins, stigmastanol, β-sitosterol, betulinic acid, D-mannitol, stigmasterol, α-alanine, aspartic acid, glutamic acid, and serine and pseudojujubogenin glycoside [7]. The plant possesses a wide variety of pharmacologically active principles including memory enhancing, tranquillizing, sedative, antidepressant, antioxidant, cognitive, anticancer, antianxiety, adaptogenic, antiepileptic, gastrointestinal effects, endocrine, gastrointestinal, smooth muscle relaxant effects, cardiovascular, analgesic, antipyretic, antidiabetic, antiarthritic, anticancer, antihypertensive, antimicrobial, antilipidemia, anti-inflammatory, neuroprotective, and hepatoprotective activities [8,9,10].

It has a very important role in Ayurvedic therapies for the treatment of cognitive disorders of aging [8,11]. Diverse mechanisms of action for its cognitive effects have been proposed, including acetylcholinesterase (AChE) inhibition, antioxidant neuroprotection, β-amyloid reduction, neurotransmitter modulation (acetylcholine [ACh], 5-hydroxytryptamine [5-HT], dopamine [DA]), choline acetyltransferase activation, and increased cerebral blood flow [12]. Theoretically, paring *B. monnieri* with a stimulant would ward off malaise, but this combination has not been tested [10]. Metabolites or active compounds from *B. monnieri* interact with the dopamine and serotonergic systems, but its main molecular mechanism concerns promoting neuron communication. It does this by increasing the growth of nerve endings, also called dendrites. Characteristics of saponins called “bacosides”, particularly bacoside A, have been considered to be the major bioactive constituents responsible for the cognitive effects of *B. monnieri* [13,14,15]. In addition, herbal medicine contains a plethora of active chemical compounds/molecules evolved through a long-term evolutionary process for safeguarding *B. monnieri* from various environmental stresses such as physio-chemical changes, the attack of pathogens, climatic changes, etc. The drug activity of herbal medicine may be due to the individual and synergistic action of phytomolecules. To the best of our knowledge, the molecular mechanism of such actions and the identification of the novel lead molecules against neurological diseases (NDs) are more difficult to study.

In light of this, and despite the significance of traditional Indian medicine, how the metabolites/compounds work and what their active human targets are are still vague. The present study aimed to explore the holistic molecular mechanism and the biological properties/pharmacological activity of phytomolecules derived from *B. monnieri* against NDs. The following issues need to be solved immediately: (i) Which active phytomolecules are involved in the regulatory processes for the treatment NDs? (ii) Which human targets are linked and modulated by the phytochemicals to achieve the biological activity and the purpose of curing NDs? With the emerging prosperity of pharmacological systems and powerful analytical tools such as cheminformatics, network pharmacology investigation allows us to understand the holistic mechanisms of Indian traditional medicine in treating complex NDs. The current study reveals deeper insights into the molecular mechanisms of active phytomolecules in treating NDs. The cheminformatics analysis was used to filter out the active and novel phytomolecules with potent pharmacological activity; also, the reliability of compound/drug–human target interactions was evaluated. The obtained potential human targets were then inputted into specific repositories to figure out their encoding NDs, pathways and holistic mechanisms of active phytomolecules. We hope that with the help of systems pharmacology and the exploration of molecular cross-talks of Indian traditional medicine, we will positively promote the development of new therapies for a mixture of neurological and other diseases in the near future.

## 2. Materials and Methods

An integrated cheminformatics and system pharmacology approach has been applied for the first time, to unveil the curative effects of Bacopa-derived, pharmacologically active compounds—consisting of: (1) target fishing and functional analysis to identify the compounds—direct target network; (2) network construction and analysis to illustrate the molecular mechanisms of Bacopa-derived compounds in treating NDs, particularly Alzheimer’s and SCA; (3) gene ontology enrichment for targets will pave the way for pathway integration analysis to reveal the regulatory mode of target players in multiple functional nodes from a signaling pathway level.

### 2.1. Collection of Phytomolecules

Detailed information on 52 phytochemicals from *B. monnieri* was collected from the literature and other web sources [16]. A list of biologically phytomolecules is depicted in Table 1.

### 2.2. Phytochemical Information Retrieval

In total, 52 biologically active plant-derived molecules from *B. monnieri* were procured from the PubChem database [17]. The 3D structures and Canonical SMILIES were collected from Molinspiration tool [18] and the PubChem database [17].

### 2.3. Human Target Imputations

The identified compounds with their canonical SMILIES were searched against human in Swiss TargetPrediction tool to retrieve the compounds in combating human targets (www.swisstargetprediction.ch/).

### 2.4. Mining of Human Targets and Its Features

Identified potential human targets were subjected onto the Expression Atlas database for retrieving human targets and their features, such as corresponding genomic transcripts, chromosome numbers, the start and end positions of the targets, and UniProt IDs [19].

### 2.5. Gene Ontology (GO) Analysis

Identified targets and their corresponding official gene symbols were subjected to the GOnet database (https://tools.dice-database.org/GOnet/) [20], to obtain GO against humans with a significant q-value threshold level of <0.05. Targets were also categorized as per GO molecular function, cellular component, and biological process according to the GOnet functional enrichment classification.

### 2.6. Network Construction

#### 2.6.1. Compound–Target-Network (C-T-N)

A C-T-N was constructed to combat NDs by expounding the multi-target therapeutic feature of the pharmacologically active compounds. In this interaction, a possible target protein and a candidate compound were linked if the protein was targeted by the phyto-compound.

#### 2.6.2. Target-Disease-Network (T-D-N)

The specific targets correlated with NDs were sorted out to explore a detailed interrelationship between potential targets and diseases, and those corresponding diseases with their potential targets were obtained from Expression Atlas database. Finally, T-D-N were built by linking all the target proteins with their relevant diseases using the previously obtained target associated disease information.

Visualization of all networks was accomplished by Cytoscape 3.7.2 [21]. In the obtained result network, nodes represent compounds, diseases and targets, whereas edges indicate the interactions between them.

#### 2.6.3. Identification of Properties of Active Compounds

Phytochemicals with their corresponding canonical SMILIES were uploaded on to the Molinspiration tool to extract the significant calculation on phytomolecules with their molecular properties, and also to predict a bioactive score for the vital targets such as GPCR ligand activity (GPCR), Kinase inhibitor activity (Ki), protease inhibitor activity (Pi), enzymes and nuclear receptors (Ncr), and the number of violations (nvio) (http://www.molinspiration.com) [18].

#### 2.6.4. Compound Comparison

Identified potential compounds with their significant molecular and bioactive properties were compared with commercially available drugs which are responsible for two different neurological diseases. The comparison revealed the pharmacologically active compounds with the help of GPCR, nvio, Ki, Pi, Ei, and Ncr properties. 

## 3. Results

### 3.1. Compound Information Retrieval

**Figure 1:**
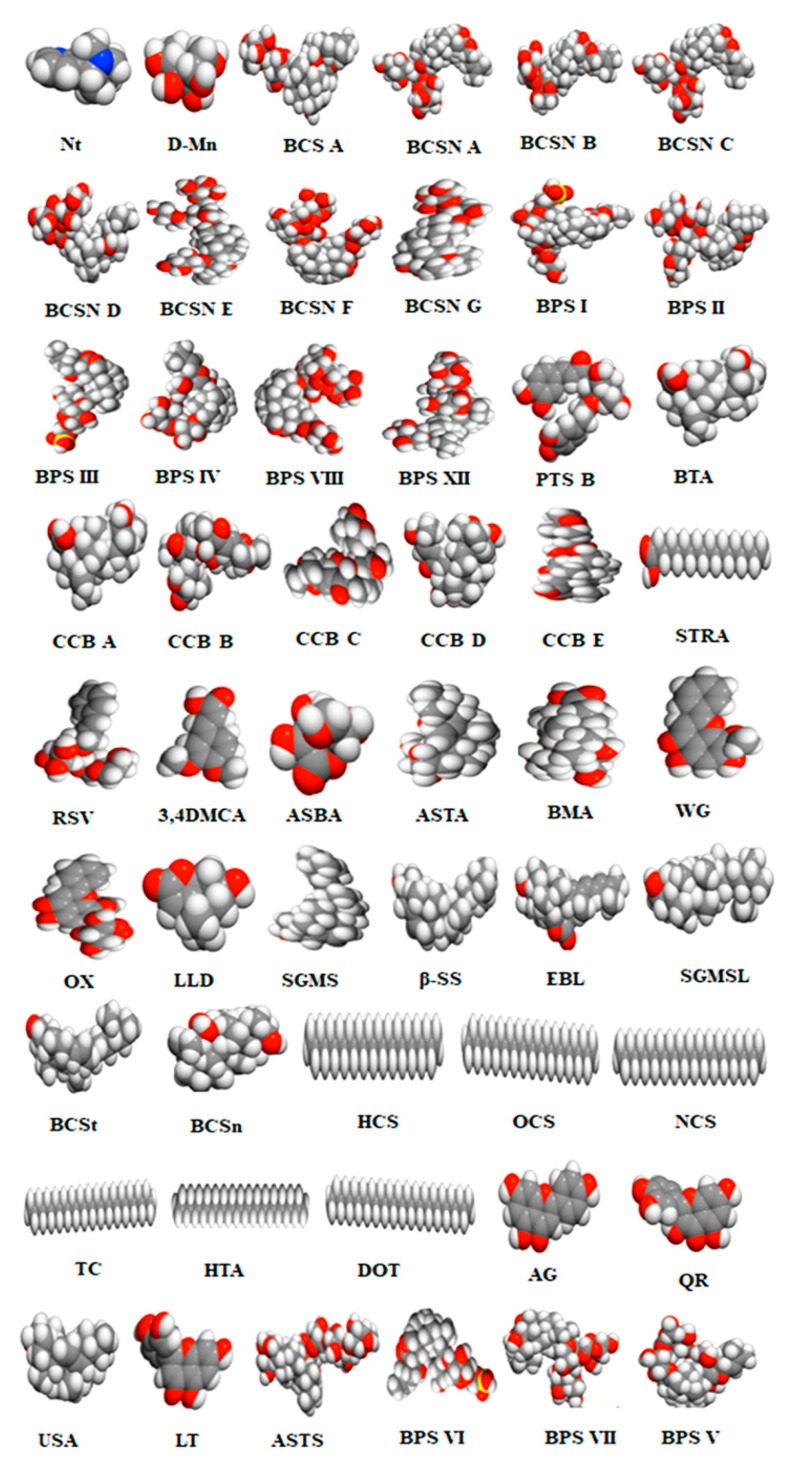
Phytomolecules and their 3D structures.

Fifty-two numbers of phytomolecules were used as a query in the PubChem database and the Molinspiration tool to retrieve the Canonical SMILIES (Table S1) and 3D structure of the compounds (Figure 1). This collected information was used for further analyses.

### 3.2. Identification Compounds Combating Human Targets

Phytomolecules targeting human receptors were identified using the Swiss TargetPrediction tool. The study identified 52 phytomolecules targeting 780 human direct receptors. A list of compound and human target information was given in Table S2. These human receptors/targets involved in various functions, especially on the signal transducer and activator, neuronal acetylcholine receptor, apoptosis regulator Bcl–2, transcription factor activities, controling the microtubule associated protein functions, and so forth. In addition, a functional description of the target proteins is given in Table S3.

### 3.3. Properties of Human Targets

Fifty-two numbers of potential compounds and 52 active human targets with their corresponding information of UniProt ID, chromosome number, start and end position and ortholog information were retrieved, and are given in Table 2. These attributes will pave the way for deciphering their detailed molecular function.

### 3.4. GO Annotation

**Figure 2:**
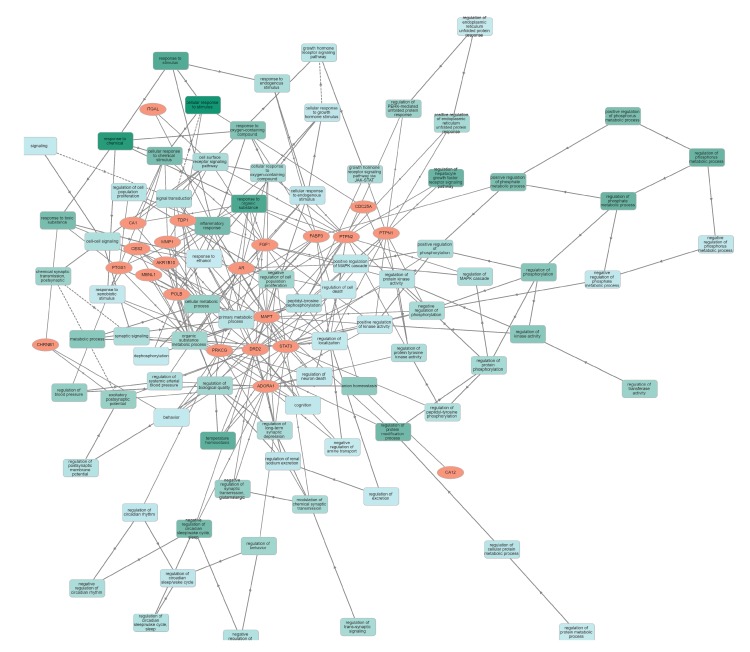
Classification human targets with their biological processes. Orange color encodes human targets; green color represents biological processes.

**Figure 3:**
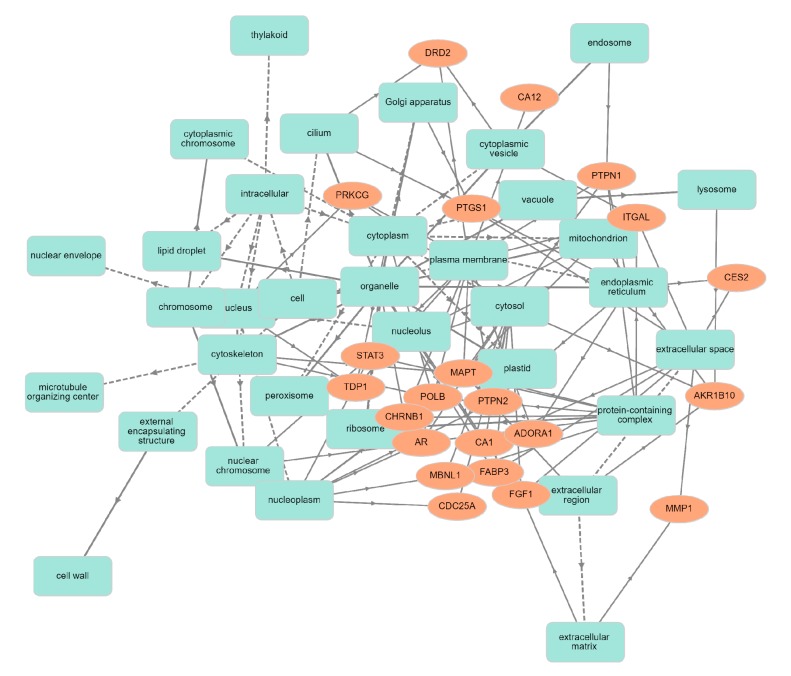
Classification human targets with corresponding cellular component. Orange color encodes human targets; green color represents cellular components.

**Figure 4:**
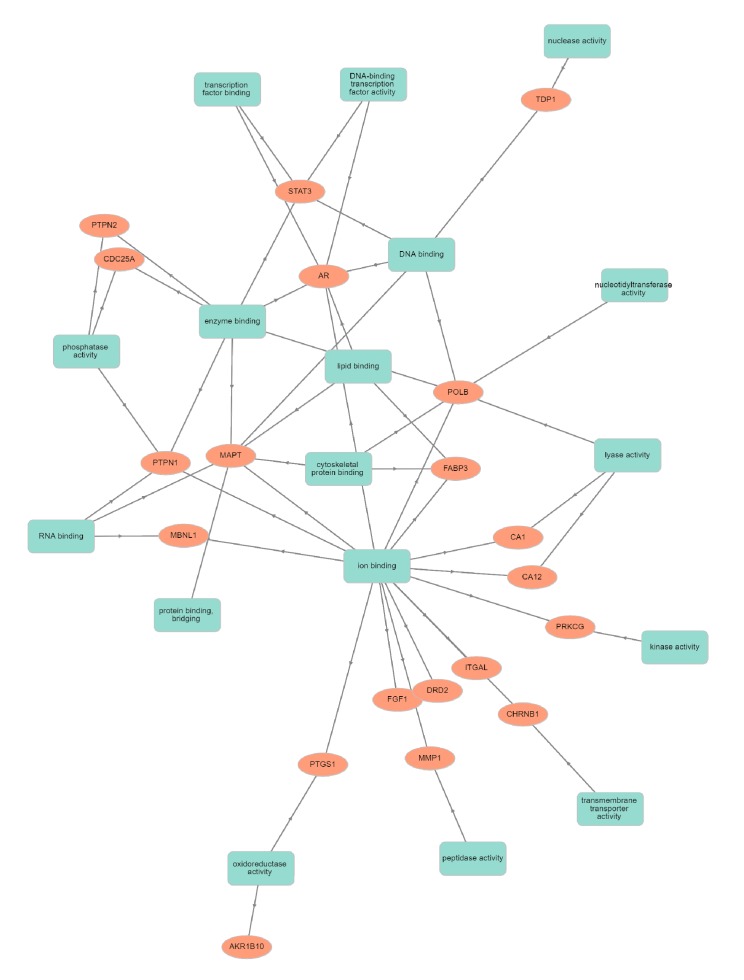
Classification human targets with encoding molecular functions. Orange color encodes human targets; green color represents various molecular functions.

Human targets with their characteristic features were analyzed by official gene symbols using the GOnet database, and showed a significant involvement of these proteins in diverse molecular functions, cellular components and biological processes. The target gene-encoding proteins were predicted to be involved in biological regulation, including in synaptic regulation, immune system processes, cell–cell signaling, responses to stimuli, and developmental growth (Figure 2). In cellular components, targets were present in the synapse, organelle, membrane, and protein-containing complex (Figure 3). The inherent molecular functions of these proteins corresponded to different types of catalytic activity, binding activity and transcription regulator activity (Figure 4). The activation effect of these compounds on their encoding human targets would definitely reduce the risk of NDs and their associated diseases. For example, these active compounds serve as an inhibitor of Aβ – peptide accumulation in the brain in the development of Alzheimer’s disease.

### 3.5. C-T-N Analysis

**Figure 5:**
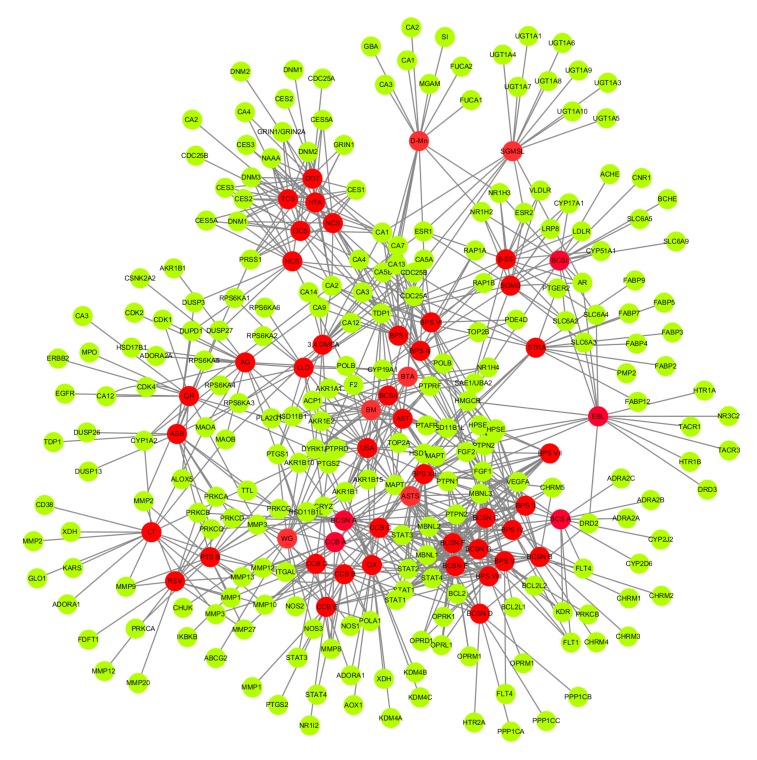
Compound target network (C-T-N). The red color represents compounds and the green color indicates targets.

The C-T-N result was displayed in Figure 5, consisting of 52 compounds and 780 human targets. Network interaction between the compounds and targets revealed the multi-target properties of elements, which is the essence of the plausible action mode of herbal drugs. As for the human targets, 52 compounds (Table 2) had a high probability, which showed the potential therapeutic effect of each compound present in Bacopa for combatting NDs, particularly Alzheimer’s and SCAby—modulating, inhibiting and or transducing the signals of these possible proteins.

### 3.6. T-D-N Analysis

**Figure 6:**
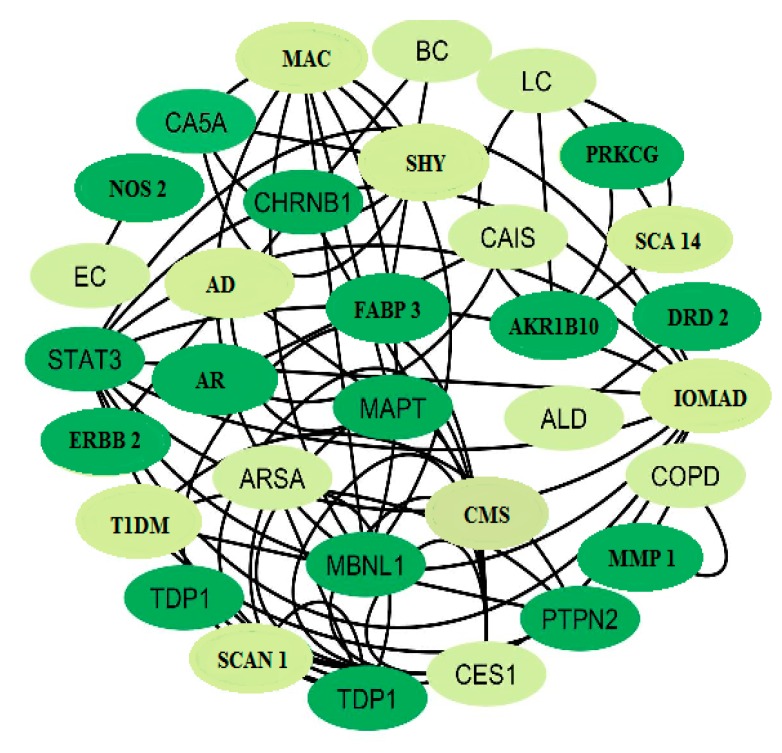
Target disease network (T-D-N). The dark color represents targets and the light color indicates diseases.

T-D-N analysis illustrated the particular mechanisms of the potential drug case by case, which were most relevant to NDs (especially Alzheimer’s and SCA) and their associated targets, and were constructed into T–D interaction (Figure 6). The results showed that the treatment of NDs has the multi-target therapeutic efficacy present in Bacopa-derived compounds. These compounds may alter the targets (yet to be characterized), and their disease-associated pathways deserve more attention in continuous therapy.

### 3.7. Features of Active Molecules and Novel Compounds

The significant calculated properties of GPCR, Ki, Pi, Ei, Ncr, and nVio were retrieved and are given in Table 3. Based on the number of violations, enzyme inhibitor activity feature scores above 0.5 were considered to be of a significant level. According to this, commercially available drugs (responsible for Alzheimer’s and SCA) and compounds were compared, and novel compounds were identified and are listed in Table 4.

## 4. Discussion

The prevalence of NDs and the ineffectiveness of allopathic medicine in dealing with multiple trait disorders mean it is crucial that we research and develop successful curative systems. The significant efficacy of Indian traditional medicine has been well established over 1000 years of practice. Though the pharmaceutical components of drugs have been extracted and purified for new drug development, this method always ends in failure due to the violation of functional drug regulation. Traditional Indian medicine treating multiple complex diseases can be also seen as a complexity fronting another complexity, which primarily speculates through the equilibrium of the entire human body system by controlling the molecular interactions between all the elements within the species. Nevertheless, the exact mode of action on the target protein and pathway stage of traditional or herbal drug mechanisms remains a roadblock to us. Therefore, in the present study, we applied a cheminformatics and integrated pharmacology approach to human systems to unravel the pharmacological role of Bacopa-derived phytomolecules in the treatment of NDs at a molecular system level. This finding also described the novel compounds for treating Alzheimer’s and SCA by inhibiting AChE, β-amyloid accumulation in the brain and degenerative changes in the cerebellum and spinal cord, respectively.

In this study, with the help of cheminformatics and the PubChem database, 52 active compounds [16] were identified. All 52 compounds strongly interact with 780 direct human targets by drug targeting. Interestingly, it was predicted to be involved in diverse biological activities against NDs (Table S3) that have not been previously reported, demonstrating the reliability of the Swiss TargetPrediction, UniProtKB and GOnet evaluation methods. A Cytoscape v3.7.2 was then executed to get the molecular interaction straight to C-T-N. The analytical results revealed the various biological processes and modes of action used by drug compounds to achieve their curative effects. Finally, in further analyses to decipher their therapeutic potential of Bacopa, a T-D-N and an NDs-associated pathway (especially for Alzheimer’s and SCA/long-term depression) were constructed (Figures S1 and S2).

Previous reports have detailed that even though the “unique/single target” compounds utilize their utmost suppressive effects on their direct human targets, they may not always produce appropriate results [22]. To the best of our knowledge, various compounds acting on multiple or the same targets hit by the same compounds gain more efficacy on binding opportunities with each other and gain more chances of affecting the entire interaction equilibrium, making the Indian traditional medicine therapy more efficacious and fruitful to society. The C-T-N analytical results showed that there are multi-target functional modules in a single active compound. Of the 52 compounds with corresponding human targets, all of them were able to act on more than 15 active targets. For example, CHRNB1 (acetylcholine ion-channel activation receptor), indicated for the activation of the cholinergic receptor nicotinic β1 subunit, has shown that myasthenic syndrome is usually the result of the prolonged activation of CHRNB1, which is mechanistically correlated with the development of NDs. Accordingly, active compounds can exert their various biological effects in this complex human system with numerous compound–target–disease interactions by controlling the associated goals and pathways and achieving curative outcomes for NDs.

NDs are not caused by a single factor, affecting different types of NDs and aggravating one another. Some stressors involved in the development of NDs are complex, but at the same time drugs have not yet emerged specifically for those conditions. Therefore, the multi-level curative effects of Bacopa phytomolecules include controlling various physiological functional regulation, transcriptional reprogramming—particularly on cerebral blood flow, and their important effectiveness in the battle against NDs from a panoramic perspective. Since modern medicine is incapable of making a breakthrough so far in the treatment of NDs, why should we not transfer our focus to Indian traditional medicine, which is well documented as confronting complex NDs?

## 5. Conclusions

*B. monnieri* showed various potential actions in the amelioration of cognitive disorders and cognitive enhancement in healthy people. Biomedical research on *B. monnieri* is still at a roadblock. Notably, our results on novel compounds—with their encoding properties, active targets, biological processes, and interactions—have opened the research floodgates with the integration of Ayurveda to the modern medicine era. This study also hypothesizes that Bacopa compounds and their combination with other substances—as is recommended by the Ayurvedic and modern medicine system—may result in synergistic effects and need to be studied further. The ethical implications of drugs which enhance cognition are vital, but should be appropriately mitigated with social and ethical considerations as field researchers enter the brave, advancing world of neural enhancement.

## Figures and Tables

**Table 1:** List of Phytomolecuels.

*S. No*	*Compounds*	*Abbreviations/Acronyms*
*1*	*Nicotine*	*Nt*
*2*	*D-Mannitol*	*D-Mn*
*3*	*Bacoside A*	*BCS A*
*4*	*Bacopasaponin A*	*BCSN A*
*5*	*Bacopasaponin B*	*BCSN B*
*6*	*Bacopasaponin C*	*BCSN C*
*7*	*Bacopasaponin D*	*BCSN D*
*8*	*Bacopasaponin E*	*BCSN E*
*9*	*Bacopasaponin F*	*BCSN F*
*10*	*Bacopasaponin G*	*BCSN G*
*11*	*Bacopaside I*	*BPS I*
*12*	*Bacopaside II*	*BPS II*
*13*	*Bacopaside III*	*BPS III*
*14*	*Bacopaside IV*	*BPS IV*
*15*	*Bacopaside V*	*BPS V*
*16*	*Bacopaside VIII*	*BPS VIII*
*17*	*Bacopaside XII*	*BPS XII*
*18*	*Plantainoside B*	*PTS B*
*19*	*Betulinic acid*	*BTA*
*20*	*Cucurbitacin A*	*CCB A*
*21*	*Cucurbitacin B*	*CCB B*
*22*	*Cucurbitacin C*	*CCB C*
*23*	*Cucurbitacin D*	*CCB D*
*24*	*Cucurbitacin E*	*CCB E*
*25*	*Stearic acid*	*STRA*
*26*	*Rosavin*	*RSV*
*27*	*3,4Dimethoxycinnamic acid*	*3,4DMCA*
*28*	*Ascorbic acid*	*ASBA*
*29*	*Asiatic acid*	*ASTA*
*30*	*Brahmic acid*	*BMA*
*31*	*Wogonin*	*WG*
*32*	*Oroxindin*	*OX*
*33*	*Loliolide*	*LLD*
*34*	*Stigmasterol*	*SGMS*
*35*	*β-sitosterol*	*β-SS*
*36*	*Ebelin lactone*	*EBL*
*37*	*Stigmastanol*	*SGMSL*
*38*	*Bacosterol*	*BCSt*
*39*	*Bacosine*	*BCSn*
*40*	*Heptacosane*	*HCS*
*41*	*Octacosane*	*OCS*
*42*	*Nonacosane*	*NCS*
*43*	*Triacontane*	*TC*
*44*	*Hentriacontane*	*HTA*
*45*	*Dotriacontane*	*DOT*
*46*	*Apigenin*	*AG*
*47*	*Quercetin*	*QR*
*48*	*Ursolic acid*	*USA*
*49*	*Luteolin*	*LT*
*50*	*Asiaticoside*	*ASTS*
*51*	*Bacopaside VI*	*BPS VI*
*52*	*Bacopaside VII*	*BPS VII*

**Table 2:** Features of human active targets.

*Compound Name*	*Target*	*Uniprot ID*	*Chr. No*	*Start*	*End*	*Orthologs*
*Nt*	*CHRNB1*	*P11230*	*17*	*7445061*	*7457707*	*Chrnb1 (Mus musculus)*
*D-Mn*	*TDP1*	*Q9NUW8*	*14*	*89954939*	*90044768*	*Tdp1 (Mus musculus)*
*BCS A*	*STAT3*	*P40763*	*17*	*42313324*	*42388568*	*Stat3 (Mus musculus)*
*BCSN A*	*MBNL1*	*Q9NR56*	*3*	*152243828*	*152465780*	*Mbnl1 (Mus musculus)*
*BCSN B*	*STAT3*	*P40763*	*17*	*42313324*	*42388568*	*Stat3 (Mus musculus)*
*BCSN C*	*PTPN2*	*P17706*	*18*	*12785478*	*12929643*	*Ptpn2 (Mus musculus)*
*BCSN D*	*PTPN1*	*P18031*	*20*	*50510321*	*50585241*	*Ptpn1 (Mus musculus)*
*BCSN E*	*MBNL1*	*Q9NR56*	*3*	*152243828*	*152465780*	*Mbnl1 (Mus musculus)*
*BCSN F*	*MBNL1*	*Q9NR56*	*3*	*152243828*	*152465780*	*Mbnl1 (Mus musculus)*
*BCSN G*	*MAPT*	*P10636*	*17*	*45894382*	*46028334*	*Mapt (Mus musculus)*
*BPS I*	*FGF1*	*P05230*	*5*	*142592178*	*142698070*	*Fgf1 (Mus musculus)*
*BPS II*	*PTPN1*	*P18031*	*20*	*50510321*	*50585241*	*Ptpn1 (Mus musculus)*
*BPS III*	*FGF1*	*P05230*	*5*	*142592178*	*142698070*	*Fgf1 (Mus musculus)*
*BPS IV*	*MBNL1*	*Q9NR56*	*3*	*152243828*	*152465780*	*Mbnl1 (Mus musculus)*
*BPS V*	*STAT3*	*P40763*	*17*	*42313324*	*42388568*	*Stat3 (Mus musculus)*
*BPS VIII*	*MBNL1*	*Q9NR56*	*3*	*152243828*	*152465780*	*Mbnl1 (Mus musculus)*
*BPS XII*	*STAT3*	*P40763*	*17*	*42313324*	*42388568*	*Stat3 (Mus musculus)*
*PTS B*	*PRKCG*	*P05129*	*19*	*53879190*	*53907652*	*Prkcg (Mus musculus),*
*BTA*	*AKR1B10*	*O60218*	*7*	*134527592*	*134541408*	*Akr1b10 (Mus musculus)*
*CCB A*	*ITGAL*	*P20701*	*16*	*30472658*	*30523185*	*Itgal (Mus musculus)*
*CCB B*	*ITGAL*	*P20701*	*16*	*30472658*	*30523185*	*Itgal (Mus musculus)*
*CCB C*	*ITGAL*	*P20701*	*16*	*30472658*	*30523185*	*Itgal (Mus musculus)*
*CCB D*	*ITGAL*	*P20701*	*16*	*30472658*	*30523185*	*Itgal (Mus musculus)*
*CCB E*	*ITGAL*	*P20701*	*16*	*30472658*	*30523185*	*Itgal (Mus musculus)*
*STRA*	*FABP3*	*P05413*	*1*	*31365625*	*31376850*	*Fabp3 (Mus musculus)*
*RSV*	*MMP1*	*P03956*	*11*	*102789920*	*102798160*	*Mmp1a (Mus musculus)*
*3,4DMCA*	*CA1*	*P00915*	*8*	*85327608*	*85379014*	*Car1 (Mus musculus)*
*ASBA*	*TDP1*	*Q9NUW8*	*14*	*89954939*	*90044768*	*Tdp1 (Mus musculus)*
*ASTA*	*AKR1B10*	*AKR1B10*	*7*	*134527592*	*134541408*	*Akr1b10 (Mus musculus)*
*BMA*	*AKR1B10*	*O60218*	*7*	*134527592*	*134541408*	*Akr1b10 (Mus musculus)*
*WG*	*PTGS1*	*P23219*	*9*	*122370530*	*122395703*	*Ptgs1 (Mus musculus)*
*OX*	*ADORA1*	*P30542*	*1*	*203090654*	*203167405*	*Adora1 (Mus musculus)*
*LLD*	*TDP1*	*Q9NUW8*	*14*	*89954939*	*90044768*	*Tdp1 (Mus musculus)*
*SGMS*	*AR*	*P10275*	*10*	*67544032*	*67730619*	*Ar (Mus musculus)*
*β-SS*	*TDP1*	*Q9NUW8*	*14*	*89954939*	*90044768*	*Tdp1 (Mus musculus)*
*EBL*	*DRD2*	*DRD2*	*11*	*113409615*	*113475691*	*Drd2 (Mus musculus)*
*SGMSL*	*AR*	*P10275*	*10*	*67544032*	*67730619*	*Ar (Mus musculus)*
*BCSt*	*TDP1*	*Q9NUW8*	*14*	*89954939*	*90044768*	*Tdp1 (Mus musculus)*
*BCSn*	*POLB*	*P06746*	*8*	*42338454*	*42371808*	*Polb (Mus musculus)*
*HCS*	*CES2*	*O00748*	*16*	*66934444*	*66945096*	*Ces2c (Mus musculus)*
*OCS*	*CES2*	*O00748*	*16*	*66934444*	*66945096*	*Ces2c (Mus musculus)*
*NCS*	*CDC25A*	*P30304*	*3*	*48157146*	*48188402*	*Cdc25a (Mus musculus)*
*TC*	*CES2*	*O00748*	*16*	*66934444*	*66945096*	*Ces2c (Mus musculus)*
*HTA*	*CES2*	*O00748*	*16*	*66934444*	*66945096*	*Ces2c (Mus musculus)*
*DOT*	*CES2*	*O00748*	*16*	*66934444*	*66945096*	*Ces2c (Mus musculus)*
*AG*	*AKR1B10*	*O60218*	*7*	*134527592*	*134541408*	*Akr1b10 (Mus musculus)*
*QR*	*CA12*	*O43570*	*15*	*63321378*	*63382161*	*Car12 (Mus musculus)*
*USA*	*POLB*	*P06746*	*8*	*42338454*	*42371808*	*Polb (Mus musculus)*
*LT*	*MMP1*	*P03956*	*11*	*102789920*	*102798160*	*Mmp1a (Mus musculus)*
*ASTS*	*STAT3*	*P40763*	*17*	*42313324*	*42388568*	*Stat3 (Mus musculus)*
*BPS VI*	*FGF1*	*P05230*	*5*	*142592178*	*142698070*	*Fgf1 (Mus musculus)*
*BPS VII*	*MAPT*	*P10636*	*17*	*45894382*	*46028334*	*Mapt (Mus musculus)*

**Table 3:** Active Compounds and its Features.

*Compound*	*GPCR lg*	*Ki*	*Ncr*	*Pi*	*Ei*	*Nvio*
*Nt*	*−0.32*	*−0.79*	*−1.57*	*−0.8*	*−0.31*	*0*
*D-Mn*	*−0.64*	*−0.88*	*−0.88*	*−0.72*	*−0.01*	*1*
*BCS A*	*−1.05*	*−2*	*−1.64*	*−0.74*	*−1.14*	*3*
*BCSN A*	*−0.77*	*−1.61*	*−1.13*	*−0.48*	*−0.68*	*3*
*BCSN B*	*−0.57*	*−1.51*	*−1.21*	*−0.36*	*−0.58*	*3*
*BCSN C*	*−2.69*	*−3.53*	*−3.34*	*−2.21*	*−2.67*	*3*
*BCSN D*	*−0.89*	*−1.87*	*−1.54*	*−0.59*	*−0.97*	*3*
*BCSN E*	*−3.6*	*−3.79*	*−3.73*	*−3.53*	*−3.51*	*3*
*BCSN F*	*−3.65*	*−3.82*	*−3.77*	*−3.6*	*−3.57*	*3*
*BCSN G*	*−0.62*	*−1.61*	*−1.24*	*−0.46*	*−0.52*	*3*
*BPS I*	*−3.13*	*−3.68*	*−3.61*	*−2.75*	*−2.9*	*3*
*BPS II*	*−2.99*	*−3.6*	*−3.48*	*−2.6*	*−2.95*	*3*
*BPS III*	*−1.65*	*−2.85*	*−2.47*	*−1.05*	*−1.7*	*3*
*BPS IV*	*−1.04*	*−1.99*	*−1.52*	*−0.76*	*−1.04*	*3*
*BPS V*	*−1.04*	*−1.99*	*−1.52*	*−0.76*	*−1.04*	*3*
*BPS VIII*	*−3.65*	*−3.82*	*−3.77*	*−3.6*	*−3.57*	*3*
*BPS XII*	*−3.65*	*−3.8*	*−3.77*	*−3.58*	*−3.59*	*3*
*PTS B*	*0.21*	*−0.04*	*0.1*	*0.13*	*0.36*	*2*
*BTA*	*0.31*	*−0.5*	*0.93*	*0.14*	*0.55*	*1*
*CCB A*	*0.45*	*−0.42*	*0.79*	*0.1*	*0.52*	*1*
*CCB B*	*0.52*	*−0.4*	*0.88*	*0.1*	*0.56*	*1*
*CCB C*	*0.42*	*−0.36*	*0.8*	*0.12*	*0.61*	*1*
*CCB D*	*0.54*	*−0.33*	*0.91*	*0.06*	*0.65*	*1*
*CCB E*	*0.47*	*−0.38*	*0.69*	*0.04*	*0.47*	*1*
*STRA*	*0.11*	*−0.2*	*0.17*	*0.06*	*0.2*	*1*
*RSV*	*0.14*	*−0.08*	*−0.08*	*0.02*	*0.34*	*1*
*3,4DMCA*	*−0.42*	*−0.66*	*−0.15*	*−0.68*	*−0.13*	*0*
*ASBA*	*−0.53*	*−1.09*	*−0.01*	*−0.81*	*0.2*	*0*
*ASTA*	*0.2*	*−0.46*	*0.91*	*0.28*	*0.66*	*0*
*BMA*	*0.25*	*−0.45*	*0.93*	*0.29*	*0.75*	*1*
*WG*	*−0.14*	*0.12*	*0.13*	*−0.31*	*0.23*	*0*
*OX*	*0.02*	*−0.06*	*0.24*	*−0.06*	*0.39*	*1*
*LLD*	*−0.45*	*−0.91*	*−0.04*	*−0.33*	*0.56*	*0*
*SGMS*	*0.12*	*−0.48*	*0.74*	*−0.02*	*0.53*	*1*
*β-SS*	*0.14*	*−0.51*	*0.73*	*0.07*	*0.51*	*1*
*EBL*	*0.33*	*−0.12*	*0.92*	*0.13*	*0.68*	*1*
*SGMSL*	*0.21*	*−0.35*	*0.65*	*0.24*	*0.48*	*1*
*BCSt*	*0.21*	*−0.37*	*0.56*	*0.15*	*0.47*	*1*
*BCSn*	*0.29*	*−0.49*	*0.91*	*0.19*	*0.56*	*1*
*HCS*	*0.04*	*−0.04*	*0.04*	*0.04*	*0.03*	*1*
*OCS*	*0.04*	*−0.04*	*0.04*	*0.04*	*0.03*	*1*
*NCS*	*0.04*	*−0.04*	*0.04*	*0.03*	*0.02*	*1*
*TC*	*0.04*	*−0.04*	*0.04*	*0.03*	*0.02*	*1*
*HTA*	*0.03*	*−0.03*	*0.04*	*0.03*	*0.02*	*1*
*DOT*	*0.03*	*−0.03*	*0.03*	*0.03*	*0.02*	*1*
*AG*	*−0.07*	*0.18*	*0.34*	*−0.25*	*0.26*	*0*
*QR*	*−0.06*	*0.28*	*0.36*	*−0.25*	*0.28*	*0*
*USA*	*0.28*	*−0.5*	*0.89*	*0.23*	*0.69*	*1*
*LT*	*−0.02*	*0.26*	*0.39*	*−0.22*	*0.28*	*0*
*ASTS*	*−3.38*	*−3.7*	*−3.55*	*−2.96*	*−3.26*	*3*
*BPS VI*	*−1.65*	*−2.85*	*−2.47*	*−1.05*	*−1.7*	*3*
*BPS VII*	*−2.73*	*−3.56*	*−3.37*	*−2.28*	*−2.62*	*3*

**Table 4:** Comparison of Drug and Novel Compounds.

*Drug*	*GPCR lg*	*Ki*	*Ncr*	*Pi*	*Ei*	*Nvio*
*Alzheimer’s Disease*						
*Tacrine*	*−0.11*	*−0.37*	*−0.93*	*−0.59*	*0.43*	*0*
*Edrophonium*	*−0.64*	*−1.59*	*−1.42*	*−0.87*	*0.69*	*0*
*Neostigmine*	*−0.22*	*−0.82*	*−0.35*	*−0.07*	*−0.66*	*0*
*Donepezil*	*0.22*	*−0.16*	*0.03*	*0.03*	*0.25*	*0*
*Pyriostigmine*	*−0.19*	*−0.86*	*−2.19*	*−0.46*	*0.59*	*0*
*Compound*	*GPCR lg*	*Ki*	*Ncr*	*Pi*	*Ei*	*nvio*
*WG*	*−0.14*	*0.12*	*0.13*	*−0.31*	*0.23*	*0*
*ASTA*	*0.2*	*−0.46*	*0.91*	*0.28*	*0.66*	*0*
*AG*	*−0.07*	*0.18*	*0.34*	*−0.25*	*0.26*	*0*
*QR*	*−0.06*	*0.28*	*0.36*	*−0.25*	*0.28*	*0*
*LLD*	*−0.45*	*−0.91*	*-0.04*	*−0.33*	*0.56*	*0*
*CCB A*	*0.45*	*−0.42*	*0.79*	*0.1*	*0.52*	*1*
*CCB B*	*0.52*	*−0.4*	*0.88*	*0.1*	*0.56*	*1*
*CCB C*	*0.42*	*−0.36*	*0.8*	*0.12*	*0.61*	*1*
*BMA*	*0.25*	*−0.45*	*0.93*	*0.29*	*0.75*	*1*
*OX*	*0.02*	*−0.06*	*0.24*	*−0.06*	*0.39*	*1*
*USA*	*0.28*	*−0.5*	*0.89*	*0.23*	*0.69*	*1*
*BCSn*	*0.29*	*−0.49*	*0.91*	*0.19*	*0.56*	*1*
*BTA*	*0.31*	*−0.5*	*0.93*	*0.14*	*0.55*	*1*
*Spinocerebellar Ataxia*						
*Drug*	*GPCR lg*	*Ki*	*Ncr*	*Pi*	*Ei*	*nvio*
*Phenytoin*	*0.07*	*−0.47*	*−0.32*	*0.01*	*−0.02*	*0*
*Primidone*	*−0.06*	*−0.58*	*−0.64*	*−0.38*	*−0.06*	*0*
*Valporic Acid*	*−0.83*	*−1.55*	*−0.78*	*−0.74*	*−0.39*	*0*
*Trimethadione*	*−0.59*	*−1.44*	*−1.53*	*−0.54*	*−0.6*	*0*
*Mephenytoin*	*−0.65*	*−1.25*	*−1.68*	*−0.76*	*−0.51*	*0*
*Lamotrigine*	*−0.16*	*0.36*	*−1.12*	*−0.84*	*0.08*	*0*
*Ethosuximide*	*−0.76*	*−2.1*	*−1.7*	*−1.1*	*−0.38*	*0*
*Ethotoin*	*−0.22*	*−0.98*	*−1.32*	*−0.43*	*−0.16*	*0*
*Oxcarbazepine*	*−0.02*	*0.1*	*−0.35*	*−0.26*	*−0.2*	*0*
*Compound*	*GPCR lg*	*Ki*	*Ncr*	*Pi*	*Ei*	*nvio*
*ASTA*	*0.2*	*−0.46*	*0.91*	*0.28*	*0.66*	*0*
*WG*	*−0.14*	*0.12*	*0.13*	*−0.31*	*0.23*	*0*
*LLD*	*−0.45*	*−0.91*	*−0.04*	*−0.33*	*0.56*	*0*
*OX*	*0.02*	*−0.06*	*0.24*	*−0.06*	*0.39*	*1*
*BCSn*	*0.29*	*−0.49*	*0.91*	*0.19*	*0.56*	*1*
*BTA*	*0.31*	*−0.5*	*0.93*	*0.14*	*0.55*	*1*
*D-Mn*	*−0.64*	*−0.88*	*−0.88*	*−0.72*	*−0.01*	*1*
*SGMS*	*0.12*	*−0.48*	*0.74*	*−0.02*	*0.53*	*1*
*β-SS*	*0.14*	*−0.51*	*0.73*	*0.07*	*0.51*	*1*
*BCSt*	*0.21*	*−0.37*	*0.56*	*0.15*	*0.47*	*1*
*HCS*	*0.04*	*−0.04*	*0.04*	*0.04*	*0.03*	*1*
*OCS*	*0.04*	*−0.04*	*0.04*	*0.04*	*0.03*	*1*
*NCS*	*0.04*	*−0.04*	*0.04*	*0.03*	*0.02*	*1*
*TC*	*0.04*	*−0.04*	*0.04*	*0.03*	*0.02*	*1*
*HTA*	*0.03*	*−0.03*	*0.04*	*0.03*	*0.02*	*1*
*DOT*	*0.03*	*−0.03*	*0.03*	*0.03*	*0.02*	*1*

## Supplementary Materials

The following are available online at https://www.mdpi.com/2218-273X/10/4/536/s1, Figure S1: title, Table S1: title, Video S1: title.
