# Supplementary material for: Bacopa monnieri and Their Bioactive Compounds Inferred Multi-Target Treatment Strategy for Neurological Diseases: A Cheminformatics and System Pharmacology Approach
Source: Biomolecules. 2020 Apr 2;10(4):536. doi: 10.3390/biom10040536 (PMC7225932; doi:10.3390/biom10040536)
Supplement: Supplementary file 1 [file biomolecules-10-00536-s001.zip › Table S3 BM merged target pdf.pdf]

SwissTargetPrediction report:

Reference:
Gfeller D., Michielin O. & Zoete V.
Shaping the interaction landscape of bioactive molecules, *Bioinformatics* (2013) 29:3073-3079.

Query Molecule

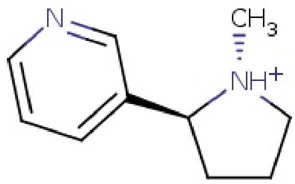

Frequency of Target Class

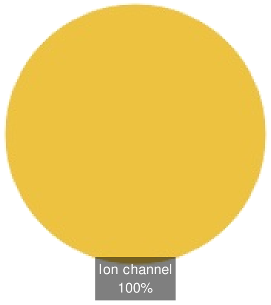

Target	Uniprot ID	Gene code	ChEMBL ID	Probability	# sim. cmpds (3D / 2D)	Target Class
Complex	P11230/Q07001/P02708/P07510	CHRNB1/CHRND/CHRNA1/CHRNG	CHEMBL1907588		3 / 1	Ion channel
Complex	P17787/P43681	CHRNB2/CHRNA4	CHEMBL1907589		53 / 96	Ion channel
Complex	P30926/P32297	CHRNB4/CHRNA3	CHEMBL1907594		14 / 26	Ion channel
Complex	P30926/P43681	CHRNB4/CHRNA4	CHEMBL1907591		2 / 4	Ion channel
Complex	P30926/Q15822	CHRNB4/CHRNA2	CHEMBL2109230		2 / 4	Ion channel
Neuronal acetylcholine receptor subunit alpha-3	P32297	CHRNA3	CHEMBL3068		11 / 2	Ion channel
Complex	P32297/P17787	CHRNA3/CHRNB2	CHEMBL2109234		4 / 4	Ion channel
Neuronal acetylcholine receptor subunit alpha-7	P36544	CHRNA7	CHEMBL2492		70 / 125	Ion channel
Neuronal acetylcholine receptor subunit alpha-4 *(by homology)*	P43681	CHRNA4	CHEMBL1882		14 / 6	Ion channel
Complex	Q15822/P17787	CHRNA2/CHRNB2	CHEMBL2109236		1 / 1	Ion channel
CHRNA7-FAM7A fusion protein *(by homology)*	Q494W8	CHRFAM7A			70 / 125	Ion channel
Neuronal acetylcholine receptor subunit alpha-2 *(by homology)*	Q15822	CHRNA2	CHEMBL1794		14 / 6	Ion channel

Neuronal acetylcholine receptor subunit beta-2 *(by homology)*	P17787	CHRNB2	CHEMBL1883		4 / 2	Ion channel
Neuronal acetylcholine receptor subunit beta-4 *(by homology)*	P30926	CHRNB4	CHEMBL3002		4 / 2	Ion channel
Neuronal acetylcholine receptor subunit alpha-10 *(by homology)*	Q9GZZ6	CHRNA10	CHEMBL2551		18 / 1	Ion channel

SwissTargetPrediction report:

Reference:
Gfeller D., Michielin O. & Zoete V.
Shaping the interaction landscape of bioactive molecules, *Bioinformatics* (2013) 29:3073-3079.

Query Molecule

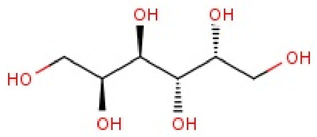

Frequency of Target Class

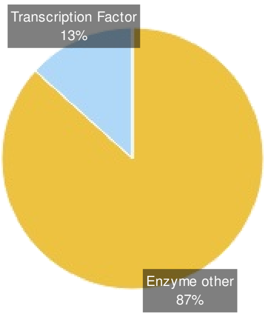

Target	Uniprot ID	Gene code	ChEMBL ID	Probability	# sim. cmpds (3D / 2D)	Target Class
Tyrosyl-DNA phosphodiesterase 1	Q9NUW8	TDP1	CHEMBL1075138		2 / 5	Enzyme
Glucosylceramidase	P04062	GBA	CHEMBL2179		2 / 2	Enzyme
Carbonic anhydrase 1	P00915	CA1	CHEMBL261		3 / 2	Enzyme
Carbonic anhydrase 2	P00918	CA2	CHEMBL205		3 / 2	Enzyme
Carbonic anhydrase 3 *(by homology)*	P07451	CA3	CHEMBL2885		3 / 2	Enzyme
Carbonic anhydrase 5A, mitochondrial *(by homology)*	P35218	CA5A	CHEMBL4789		3 / 2	Enzyme
Carbonic anhydrase 7 *(by homology)*	P43166	CA7	CHEMBL2326		3 / 2	Enzyme
Carbonic anhydrase 13 *(by homology)*	Q8N1Q1	CA13	CHEMBL3912		3 / 2	Enzyme
Carbonic anhydrase 5B, mitochondrial *(by homology)*	Q9Y2D0	CA5B	CHEMBL3969		3 / 2	Enzyme
Tissue alpha-L-fucosidase	P04066	FUCA1	CHEMBL4176		4 / 5	Enzyme
Plasma alpha-L-fucosidase *(by homology)*	Q9BTY2	FUCA2	CHEMBL2271		4 / 5	Enzyme
Oxysterols receptor LXR-beta	P55055	NR1H2	CHEMBL4093		0 / 2	Transcription Factor
Oxysterols receptor LXR-alpha	Q13133	NR1H3	CHEMBL2808		0 / 2	Transcription Factor
Glucoamylase	O43451	MGAM	CHEMBL2074		2 / 3	Enzyme
Sucrase	P14410	SI	CHEMBL2748		2 / 3	Enzyme

SwissTargetPrediction report:

Reference:
Gfeller D., Michielin O. & Zoete V.
Shaping the interaction landscape of bioactive molecules, *Bioinformatics* (2013) 29:3073-3079.

Query Molecule

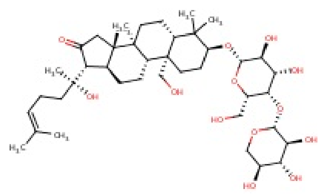

Frequency of Target Class

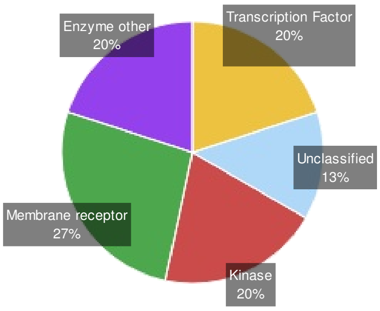

Target	Uniprot ID	Gene code	ChEMBL ID	Probability	# sim. cmpds (3D / 2D)	Target Class
Signal transducer and activator of transcription 3	P40763	STAT3	CHEMBL4026		1 / 4	Transcription Factor
Signal transducer and activator of transcription 1-alpha/beta *(by homology)*	P42224	STAT1	CHEMBL6101		1 / 4	Unclassified
Signal transducer and activator of transcription 2 *(by homology)*	P52630	STAT2			1 / 4	Transcription Factor
Signal transducer and activator of transcription 4 *(by homology)*	Q14765	STAT4			1 / 4	Transcription Factor
Microtubule-associated protein tau	P10636	MAPT	CHEMBL1293224		1 / 19	Unclassified
Vascular endothelial growth factor receptor 1 *(by homology)*	P17948	FLT1	CHEMBL1868		5 / 1	Tyr Kinase
Vascular endothelial growth factor receptor 2	P35968	KDR	CHEMBL279		5 / 1	Tyr Kinase
Vascular endothelial growth factor receptor 3 *(by homology)*	P35916	FLT4	CHEMBL1955		5 / 1	Tyr Kinase
Alpha-2A adrenergic receptor	P08913	ADRA2A	CHEMBL1867		2 / 2	Membrane receptor
Alpha-2B adrenergic receptor	P18089	ADRA2B	CHEMBL1942		2 / 2	Membrane receptor
Alpha-2C adrenergic receptor	P18825	ADRA2C	CHEMBL1916		2 / 2	Membrane receptor
Cytochrome P450 2D6	P10635	CYP2D6	CHEMBL289		1 / 1	Enzyme
Cytochrome P450 2J2	P51589	CYP2J2	CHEMBL3491		1 / 1	Enzyme
Platelet-activating factor receptor	P25105	PTAFR	CHEMBL250		1 / 39	Membrane receptor
Corticosteroid 11-beta-dehydrogenase isozyme 1	P28845	HSD11B1	CHEMBL4235		0 / 4	Enzyme

SwissTargetPrediction report:

Reference:
Gfeller D., Michielin O. & Zoete V.
Shaping the interaction landscape of bioactive molecules, *Bioinformatics* (2013) 29:3073-3079.

Query Molecule

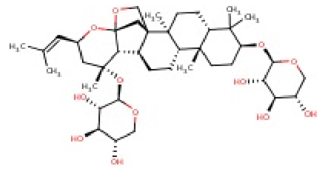

Frequency of Target Class

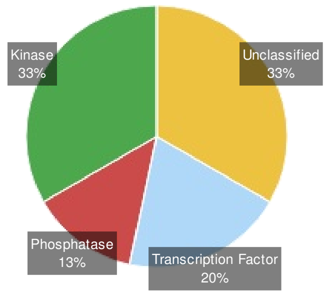

Target	Uniprot ID	Gene code	ChEMBL ID	Probability	# sim. cmpds (3D / 2D)	Target Class
Muscleblind-like protein 1	Q9NR56	MBNL1	CHEMBL1293317		1 / 2	Unclassified
Muscleblind-like protein 2 *(by homology)*	Q5VZF2	MBNL2			1 / 2	Unclassified
Muscleblind-like protein 3 *(by homology)*	Q9NUK0	MBNL3			1 / 2	Unclassified
Signal transducer and activator of transcription 3	P40763	STAT3	CHEMBL4026		0 / 4	Transcription Factor
Signal transducer and activator of transcription 1-alpha/beta *(by homology)*	P42224	STAT1	CHEMBL6101		0 / 4	Unclassified
Signal transducer and activator of transcription 2 *(by homology)*	P52630	STAT2			0 / 4	Transcription Factor
Signal transducer and activator of transcription 4 *(by homology)*	Q14765	STAT4			0 / 4	Transcription Factor
Tyrosine-protein phosphatase non-receptor type 2	P17706	PTPN2	CHEMBL3807		1 / 7	Tyr Phosphatase
Tyrosine-protein phosphatase non-receptor type 1	P18031	PTPN1	CHEMBL335		1 / 7	Tyr Phosphatase
Microtubule-associated protein tau	P10636	MAPT	CHEMBL1293224		0 / 12	Unclassified
Protein kinase C gamma type *(by homology)*	P05129	PRKCG	CHEMBL2938		1 / 123	Ser_Thr Kinase
Protein kinase C beta type *(by homology)*	P05771	PRKCB	CHEMBL3045		1 / 123	Ser_Thr Kinase
Protein kinase C alpha type	P17252	PRKCA	CHEMBL299		1 / 123	Ser_Thr Kinase
Protein kinase C theta type *(by homology)*	Q04759	PRKCQ	CHEMBL3920		1 / 126	Ser_Thr Kinase
Protein kinase C delta type regulatory subunit *(by homology)*	Q05655	PRKCD	CHEMBL2996		1 / 126	Ser_Thr Kinase

SwissTargetPrediction report:

Reference:
Gfeller D., Michielin O. & Zoete V.
Shaping the interaction landscape of bioactive molecules, *Bioinformatics* (2013) 29:3073-3079.

Query Molecule

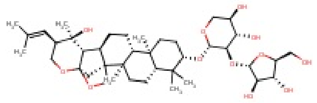

Frequency of Target Class

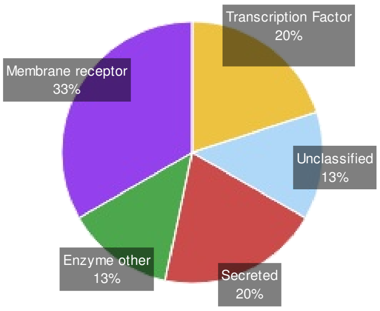

Target	Uniprot ID	Gene code	ChEMBL ID	Probability	# sim. cmpds (3D / 2D)	Target Class
Signal transducer and activator of transcription 3	P40763	STAT3	CHEMBL4026		0 / 4	Transcription Factor
Signal transducer and activator of transcription 1-alpha/beta *(by homology)*	P42224	STAT1	CHEMBL6101		0 / 4	Unclassified
Signal transducer and activator of transcription 2 *(by homology)*	P52630	STAT2			0 / 4	Transcription Factor
Signal transducer and activator of transcription 4 *(by homology)*	Q14765	STAT4			0 / 4	Transcription Factor
Fibroblast growth factor 1	P05230	FGF1	CHEMBL2120		0 / 7	Secreted
Fibroblast growth factor 2	P09038	FGF2	CHEMBL3107		0 / 7	Secreted
Vascular endothelial growth factor A	P15692	VEGFA	CHEMBL1783		0 / 2	Secreted
Heparanase 8 kDa subunit	Q9Y251	HPSE	CHEMBL3921		0 / 4	Enzyme
Inactive heparanase-2 *(by homology)*	Q8WWQ2	HPSE2			0 / 4	Enzyme
Muscarinic acetylcholine receptor M5 *(by homology)*	P08912	CHRM5	CHEMBL2035		0 / 2	Membrane receptor
Muscarinic acetylcholine receptor M1	P11229	CHRM1	CHEMBL216		0 / 2	Membrane receptor
Muscarinic acetylcholine receptor M3 *(by homology)*	P20309	CHRM3	CHEMBL245		0 / 2	Membrane receptor
Muscarinic acetylcholine receptor M2 *(by homology)*	P08172	CHRM2	CHEMBL211		0 / 2	Membrane receptor
Muscarinic acetylcholine receptor M4 *(by homology)*	P08173	CHRM4	CHEMBL1821		0 / 2	Membrane receptor
Muscleblind-like protein 1	Q9NR56	MBNL1	CHEMBL1293317		0 / 3	Unclassified

SwissTargetPrediction report:

Reference:
Gfeller D., Michielin O. & Zoete V.
Shaping the interaction landscape of bioactive molecules, *Bioinformatics* (2013) 29:3073-3079.

Query Molecule

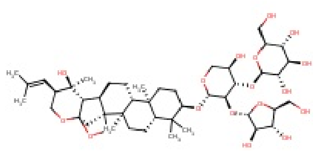

Frequency of Target Class

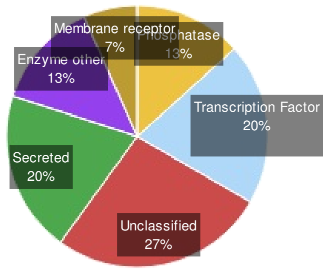

Target	Uniprot ID	Gene code	ChEMBL ID	Probability	# sim. cmpds (3D / 2D)	Target Class
Tyrosine-protein phosphatase non-receptor type 2 *(by homology)*	P17706	PTPN2	CHEMBL3807		6 / 2	Tyr Phosphatase
Tyrosine-protein phosphatase non-receptor type 1	P18031	PTPN1	CHEMBL335		6 / 2	Tyr Phosphatase
Signal transducer and activator of transcription 3	P40763	STAT3	CHEMBL4026		0 / 4	Transcription Factor
Signal transducer and activator of transcription 1-alpha/beta *(by homology)*	P42224	STAT1	CHEMBL6101		0 / 4	Unclassified
Signal transducer and activator of transcription 2 *(by homology)*	P52630	STAT2			0 / 4	Transcription Factor
Signal transducer and activator of transcription 4 *(by homology)*	Q14765	STAT4			0 / 4	Transcription Factor
Fibroblast growth factor 1	P05230	FGF1	CHEMBL2120		0 / 8	Secreted
Fibroblast growth factor 2	P09038	FGF2	CHEMBL3107		0 / 8	Secreted
Vascular endothelial growth factor A	P15692	VEGFA	CHEMBL1783		0 / 2	Secreted
Heparanase 8 kDa subunit	Q9Y251	HPSE	CHEMBL3921		0 / 4	Enzyme
Inactive heparanase-2 *(by homology)*	Q8WWQ2	HPSE2			0 / 4	Enzyme
Muscleblind-like protein 1	Q9NR56	MBNL1	CHEMBL1293317		0 / 3	Unclassified
Muscleblind-like protein 2 *(by homology)*	Q5VZF2	MBNL2			0 / 3	Unclassified
Muscleblind-like protein 3 *(by homology)*	Q9NUK0	MBNL3			0 / 3	Unclassified
Muscarinic acetylcholine receptor M5 *(by homology)*	P08912	CHRM5	CHEMBL2035		0 / 2	Membrane receptor

SwissTargetPrediction report:

Reference:
Gfeller D., Michielin O. & Zoete V.
Shaping the interaction landscape of bioactive molecules, *Bioinformatics* (2013) 29:3073-3079.

Query Molecule

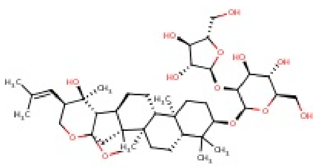

Frequency of Target Class

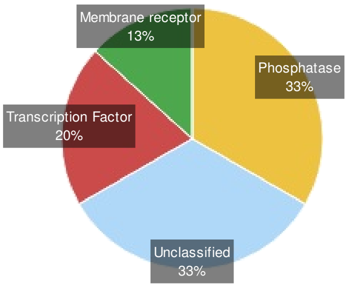

Target	Uniprot ID	Gene code	ChEMBL ID	Probability	# sim. cmpds (3D / 2D)	Target Class
Tyrosine-protein phosphatase non-receptor type 1	P18031	PTPN1	CHEMBL335		4 / 2	Tyr Phosphatase
Tyrosine-protein phosphatase non-receptor type 2 *(by homology)*	P17706	PTPN2	CHEMBL3807		4 / 2	Tyr Phosphatase
Muscleblind-like protein 1	Q9NR56	MBNL1	CHEMBL1293317		1 / 3	Unclassified
Muscleblind-like protein 2 *(by homology)*	Q5VZF2	MBNL2			1 / 3	Unclassified
Muscleblind-like protein 3 *(by homology)*	Q9NUK0	MBNL3			1 / 3	Unclassified
Microtubule-associated protein tau	P10636	MAPT	CHEMBL1293224		1 / 10	Unclassified
Serine/threonine-protein phosphatase PP1-gamma catalytic subunit	P36873	PPP1CC	CHEMBL4438		1 / 2	Ser_Thr Phosphatase
Serine/threonine-protein phosphatase PP1-alpha catalytic subunit	P62136	PPP1CA	CHEMBL2164		1 / 2	Ser_Thr Phosphatase
Serine/threonine-protein phosphatase PP1-beta catalytic subunit *(by homology)*	P62140	PPP1CB	CHEMBL4546		1 / 2	Ser_Thr Phosphatase
Signal transducer and activator of transcription 3	P40763	STAT3	CHEMBL4026		0 / 4	Transcription Factor
Signal transducer and activator of transcription 1-alpha/beta *(by homology)*	P42224	STAT1	CHEMBL6101		0 / 4	Unclassified
Signal transducer and activator of transcription 2 *(by homology)*	P52630	STAT2			0 / 4	Transcription Factor
Signal transducer and activator of transcription 4 *(by homology)*	Q14765	STAT4			0 / 4	Transcription Factor
D(2) dopamine receptor	P14416	DRD2	CHEMBL217		4 / 2	Membrane receptor
5-hydroxytryptamine receptor 2A	P28223	HTR2A	CHEMBL224		1 / 1	Membrane receptor

SwissTargetPrediction report:

Reference:
Gfeller D., Michielin O. & Zoete V.
Shaping the interaction landscape of bioactive molecules, *Bioinformatics* (2013) 29:3073-3079.

Query Molecule

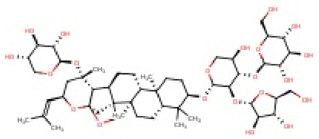

Frequency of Target Class

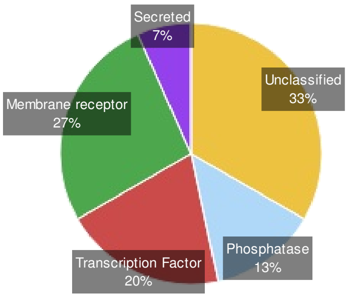

Target	Uniprot ID	Gene code	ChEMBL ID	Probability	# sim. cmpds (3D / 2D)	Target Class
Muscleblind-like protein 1	Q9NR56	MBNL1	CHEMBL1293317		0 / 2	Unclassified
Muscleblind-like protein 2 *(by homology)*	Q5VZF2	MBNL2			0 / 2	Unclassified
Muscleblind-like protein 3 *(by homology)*	Q9NUK0	MBNL3			0 / 2	Unclassified
Tyrosine-protein phosphatase non-receptor type 1	P18031	PTPN1	CHEMBL335		2 / 6	Tyr Phosphatase
Tyrosine-protein phosphatase non-receptor type 2 *(by homology)*	P17706	PTPN2	CHEMBL3807		2 / 6	Tyr Phosphatase
Signal transducer and activator of transcription 3	P40763	STAT3	CHEMBL4026		0 / 4	Transcription Factor
Signal transducer and activator of transcription 1-alpha/beta *(by homology)*	P42224	STAT1	CHEMBL6101		0 / 4	Unclassified
Signal transducer and activator of transcription 2 *(by homology)*	P52630	STAT2			0 / 4	Transcription Factor
Signal transducer and activator of transcription 4 *(by homology)*	Q14765	STAT4			0 / 4	Transcription Factor
Microtubule-associated protein tau	P10636	MAPT	CHEMBL1293224		0 / 12	Unclassified
Mu-type opioid receptor	P35372	OPRM1	CHEMBL233		1 / 6	Membrane receptor
Delta-type opioid receptor	P41143	OPRD1	CHEMBL236		1 / 6	Membrane receptor
Kappa-type opioid receptor	P41145	OPRK1	CHEMBL237		1 / 6	Membrane receptor
Nociceptin receptor *(by homology)*	P41146	OPRL1	CHEMBL2014		1 / 6	Membrane receptor
Fibroblast growth factor 1	P05230	FGF1	CHEMBL2120		0 / 6	Secreted

SwissTargetPrediction report:

Reference:
Gfeller D., Michielin O. & Zoete V.
Shaping the interaction landscape of bioactive molecules, *Bioinformatics* (2013) 29:3073-3079.

Query Molecule

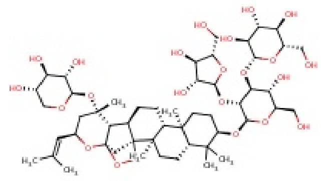

Frequency of Target Class

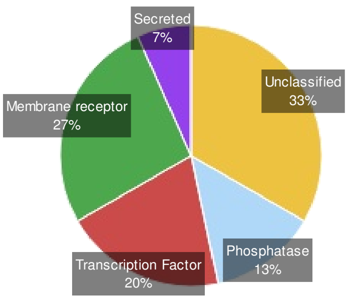

Target	Uniprot ID	Gene code	ChEMBL ID	Probability	# sim. cmpds (3D / 2D)	Target Class
Muscleblind-like protein 1	Q9NR56	MBNL1	CHEMBL1293317		0 / 2	Unclassified
Muscleblind-like protein 2 *(by homology)*	Q5VZF2	MBNL2			0 / 2	Unclassified
Muscleblind-like protein 3 *(by homology)*	Q9NUK0	MBNL3			0 / 2	Unclassified
Tyrosine-protein phosphatase non-receptor type 1	P18031	PTPN1	CHEMBL335		2 / 6	Tyr Phosphatase
Tyrosine-protein phosphatase non-receptor type 2 *(by homology)*	P17706	PTPN2	CHEMBL3807		2 / 6	Tyr Phosphatase
Signal transducer and activator of transcription 3	P40763	STAT3	CHEMBL4026		0 / 4	Transcription Factor
Signal transducer and activator of transcription 1-alpha/beta *(by homology)*	P42224	STAT1	CHEMBL6101		0 / 4	Unclassified
Signal transducer and activator of transcription 2 *(by homology)*	P52630	STAT2			0 / 4	Transcription Factor
Signal transducer and activator of transcription 4 *(by homology)*	Q14765	STAT4			0 / 4	Transcription Factor
Microtubule-associated protein tau	P10636	MAPT	CHEMBL1293224		0 / 12	Unclassified
Mu-type opioid receptor	P35372	OPRM1	CHEMBL233		1 / 6	Membrane receptor
Delta-type opioid receptor	P41143	OPRD1	CHEMBL236		1 / 6	Membrane receptor
Kappa-type opioid receptor	P41145	OPRK1	CHEMBL237		1 / 6	Membrane receptor
Nociceptin receptor *(by homology)*	P41146	OPRL1	CHEMBL2014		1 / 6	Membrane receptor
Fibroblast growth factor 1	P05230	FGF1	CHEMBL2120		0 / 6	Secreted

SwissTargetPrediction report:

Reference:
Gfeller D., Michielin O. & Zoete V.
Shaping the interaction landscape of bioactive molecules, *Bioinformatics* (2013) 29:3073-3079.

Query Molecule

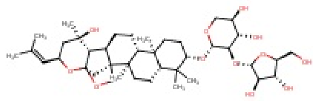

Frequency of Target Class

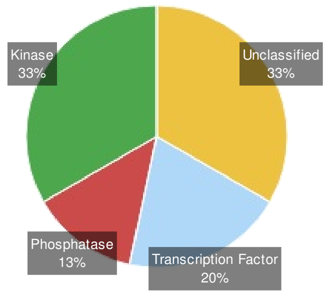

Target	Uniprot ID	Gene code	ChEMBL ID	Probability	# sim. cmpds (3D / 2D)	Target Class
Microtubule-associated protein tau	P10636	MAPT	CHEMBL1293224		1 / 12	Unclassified
Signal transducer and activator of transcription 3	P40763	STAT3	CHEMBL4026		2 / 4	Transcription Factor
Signal transducer and activator of transcription 1-alpha/beta *(by homology)*	P42224	STAT1	CHEMBL6101		2 / 4	Unclassified
Signal transducer and activator of transcription 2 *(by homology)*	P52630	STAT2			2 / 4	Transcription Factor
Signal transducer and activator of transcription 4 *(by homology)*	Q14765	STAT4			2 / 4	Transcription Factor
Muscleblind-like protein 1	Q9NR56	MBNL1	CHEMBL1293317		0 / 2	Unclassified
Muscleblind-like protein 2 *(by homology)*	Q5VZF2	MBNL2			0 / 2	Unclassified
Muscleblind-like protein 3 *(by homology)*	Q9NUK0	MBNL3			0 / 2	Unclassified
Tyrosine-protein phosphatase non-receptor type 1	P18031	PTPN1	CHEMBL335		1 / 6	Tyr Phosphatase
Tyrosine-protein phosphatase non-receptor type 2 *(by homology)*	P17706	PTPN2	CHEMBL3807		1 / 6	Tyr Phosphatase
Vascular endothelial growth factor receptor 1	P17948	FLT1	CHEMBL1868		1 / 1	Tyr Kinase
Vascular endothelial growth factor receptor 2	P35968	KDR	CHEMBL279		1 / 1	Tyr Kinase
Vascular endothelial growth factor receptor 3 *(by homology)*	P35916	FLT4	CHEMBL1955		1 / 1	Tyr Kinase
Protein kinase C gamma type *(by homology)*	P05129	PRKCG	CHEMBL2938		1 / 137	Ser_Thr Kinase
Protein kinase C beta type	P05771	PRKCB	CHEMBL3045		1 / 137	Ser_Thr Kinase

SwissTargetPrediction report:

Reference:
Gfeller D., Michielin O. & Zoete V.
Shaping the interaction landscape of bioactive molecules, *Bioinformatics* (2013) 29:3073-3079.

Query Molecule

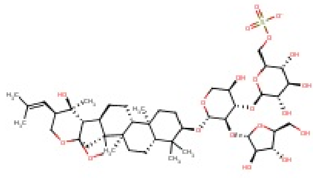

Frequency of Target Class

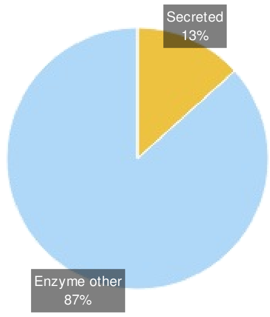

Target	Uniprot ID	Gene code	ChEMBL ID	Probability	# sim. cmpds (3D / 2D)	Target Class
Fibroblast growth factor 1	P05230	FGF1	CHEMBL2120		0 / 13	Secreted
Fibroblast growth factor 2	P09038	FGF2	CHEMBL3107		0 / 13	Secreted
Heparanase 8 kDa subunit	Q9Y251	HPSE	CHEMBL3921		0 / 7	Enzyme
Inactive heparanase-2 *(by homology)*	Q8WWQ2	HPSE2			0 / 7	Enzyme
Carbonic anhydrase 1	P00915	CA1	CHEMBL261		2 / 21	Enzyme
Carbonic anhydrase 2	P00918	CA2	CHEMBL205		2 / 21	Enzyme
Carbonic anhydrase 5A, mitochondrial *(by homology)*	P35218	CA5A	CHEMBL4789		2 / 21	Enzyme
Carbonic anhydrase 7 *(by homology)*	P43166	CA7	CHEMBL2326		2 / 21	Enzyme
Carbonic anhydrase 13 *(by homology)*	Q8N1Q1	CA13	CHEMBL3912		2 / 21	Enzyme
Carbonic anhydrase 5B, mitochondrial *(by homology)*	Q9Y2D0	CA5B	CHEMBL3969		2 / 21	Enzyme
Carbonic anhydrase 3 *(by homology)*	P07451	CA3	CHEMBL2885		2 / 21	Enzyme
Carbonic anhydrase 4	P22748	CA4	CHEMBL3729		2 / 1	Enzyme
Carbonic anhydrase 12	O43570	CA12	CHEMBL3242		0 / 13	Enzyme
Carbonic anhydrase 9	Q16790	CA9	CHEMBL3594		0 / 15	Enzyme
Carbonic anhydrase 14 *(by homology)*	Q9ULX7	CA14	CHEMBL3510		0 / 13	Enzyme

SwissTargetPrediction report:

Reference:
Gfeller D., Michielin O. & Zoete V.
Shaping the interaction landscape of bioactive molecules, *Bioinformatics* (2013) 29:3073-3079.

Query Molecule

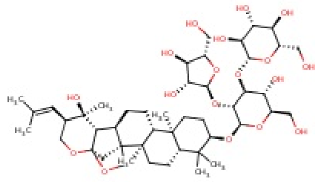

Frequency of Target Class

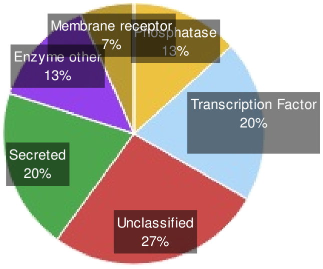

Target	Uniprot ID	Gene code	ChEMBL ID	Probability	# sim. cmpds (3D / 2D)	Target Class
Tyrosine-protein phosphatase non-receptor type 1	P18031	PTPN1	CHEMBL335		4 / 2	Tyr Phosphatase
Tyrosine-protein phosphatase non-receptor type 2 *(by homology)*	P17706	PTPN2	CHEMBL3807		4 / 2	Tyr Phosphatase
Signal transducer and activator of transcription 3	P40763	STAT3	CHEMBL4026		0 / 4	Transcription Factor
Signal transducer and activator of transcription 1-alpha/beta *(by homology)*	P42224	STAT1	CHEMBL6101		0 / 4	Unclassified
Signal transducer and activator of transcription 2 *(by homology)*	P52630	STAT2			0 / 4	Transcription Factor
Signal transducer and activator of transcription 4 *(by homology)*	Q14765	STAT4			0 / 4	Transcription Factor
Fibroblast growth factor 1	P05230	FGF1	CHEMBL2120		0 / 8	Secreted
Fibroblast growth factor 2	P09038	FGF2	CHEMBL3107		0 / 8	Secreted
Vascular endothelial growth factor A	P15692	VEGFA	CHEMBL1783		0 / 2	Secreted
Heparanase 8 kDa subunit	Q9Y251	HPSE	CHEMBL3921		0 / 4	Enzyme
Inactive heparanase-2 *(by homology)*	Q8WWQ2	HPSE2			0 / 4	Enzyme
Muscleblind-like protein 1	Q9NR56	MBNL1	CHEMBL1293317		0 / 3	Unclassified
Muscleblind-like protein 2 *(by homology)*	Q5VZF2	MBNL2			0 / 3	Unclassified
Muscleblind-like protein 3 *(by homology)*	Q9NUK0	MBNL3			0 / 3	Unclassified
Muscarinic acetylcholine receptor M5 *(by homology)*	P08912	CHRM5	CHEMBL2035		0 / 2	Membrane receptor

SwissTargetPrediction report:

Reference:
Gfeller D., Michielin O. & Zoete V.
Shaping the interaction landscape of bioactive molecules, *Bioinformatics* (2013) 29:3073-3079.

Query Molecule

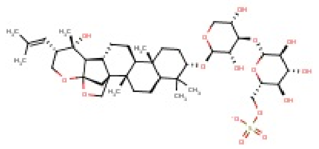

Frequency of Target Class

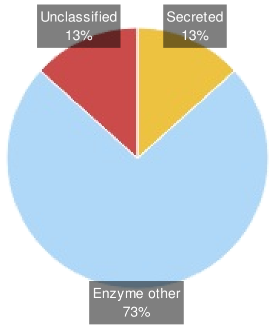

Target	Uniprot ID	Gene code	ChEMBL ID	Probability	# sim. cmpds (3D / 2D)	Target Class
Fibroblast growth factor 1	P05230	FGF1	CHEMBL2120		0 / 12	Secreted
Fibroblast growth factor 2	P09038	FGF2	CHEMBL3107		0 / 12	Secreted
Heparanase 8 kDa subunit	Q9Y251	HPSE	CHEMBL3921		0 / 7	Enzyme
Inactive heparanase-2 *(by homology)*	Q8WWQ2	HPSE2			0 / 7	Enzyme
Carbonic anhydrase 1	P00915	CA1	CHEMBL261		1 / 21	Enzyme
Carbonic anhydrase 2	P00918	CA2	CHEMBL205		1 / 21	Enzyme
Carbonic anhydrase 5A, mitochondrial *(by homology)*	P35218	CA5A	CHEMBL4789		1 / 21	Enzyme
Carbonic anhydrase 7 *(by homology)*	P43166	CA7	CHEMBL2326		1 / 21	Enzyme
Carbonic anhydrase 13 *(by homology)*	Q8N1Q1	CA13	CHEMBL3912		1 / 21	Enzyme
Carbonic anhydrase 5B, mitochondrial *(by homology)*	Q9Y2D0	CA5B	CHEMBL3969		1 / 21	Enzyme
Carbonic anhydrase 3 *(by homology)*	P07451	CA3	CHEMBL2885		1 / 21	Enzyme
Carbonic anhydrase 4	P22748	CA4	CHEMBL3729		1 / 1	Enzyme
Ras-related protein Rap-1A	P62834	RAP1A	CHEMBL1255139		0 / 1	Unclassified
cAMP-specific 3',5'-cyclic phosphodiesterase 4D	Q08499	PDE4D	CHEMBL288		0 / 1	Enzyme
Ras-related protein Rap-1b *(by homology)*	P61224	RAP1B			0 / 1	Unclassified

SwissTargetPrediction report:

Reference:
Gfeller D., Michielin O. & Zoete V.
Shaping the interaction landscape of bioactive molecules, *Bioinformatics* (2013) 29:3073-3079.

Query Molecule

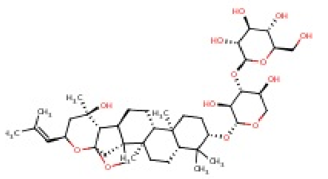

Frequency of Target Class

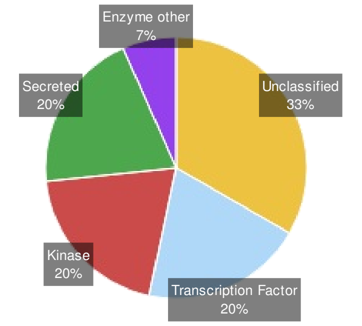

Target	Uniprot ID	Gene code	ChEMBL ID	Probability	# sim. cmpds (3D / 2D)	Target Class
Muscleblind-like protein 1	Q9NR56	MBNL1	CHEMBL1293317		0 / 2	Unclassified
Muscleblind-like protein 2 *(by homology)*	Q5VZF2	MBNL2			0 / 2	Unclassified
Muscleblind-like protein 3 *(by homology)*	Q9NUK0	MBNL3			0 / 2	Unclassified
Signal transducer and activator of transcription 3	P40763	STAT3	CHEMBL4026		0 / 4	Transcription Factor
Signal transducer and activator of transcription 1-alpha/beta *(by homology)*	P42224	STAT1	CHEMBL6101		0 / 4	Unclassified
Signal transducer and activator of transcription 2 *(by homology)*	P52630	STAT2			0 / 4	Transcription Factor
Signal transducer and activator of transcription 4 *(by homology)*	Q14765	STAT4			0 / 4	Transcription Factor
Vascular endothelial growth factor receptor 1	P17948	FLT1	CHEMBL1868		1 / 1	Tyr Kinase
Vascular endothelial growth factor receptor 2	P35968	KDR	CHEMBL279		1 / 1	Tyr Kinase
Vascular endothelial growth factor receptor 3 *(by homology)*	P35916	FLT4	CHEMBL1955		1 / 1	Tyr Kinase
Microtubule-associated protein tau	P10636	MAPT	CHEMBL1293224		0 / 12	Unclassified
Fibroblast growth factor 1	P05230	FGF1	CHEMBL2120		0 / 6	Secreted
Fibroblast growth factor 2	P09038	FGF2	CHEMBL3107		0 / 6	Secreted
Vascular endothelial growth factor A	P15692	VEGFA	CHEMBL1783		0 / 2	Secreted
Heparanase 8 kDa subunit	Q9Y251	HPSE	CHEMBL3921		0 / 4	Enzyme

SwissTargetPrediction report:

Reference:
Gfeller D., Michielin O. & Zoete V.
Shaping the interaction landscape of bioactive molecules, *Bioinformatics* (2013) 29:3073-3079.

Query Molecule

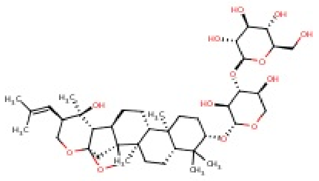

Frequency of Target Class

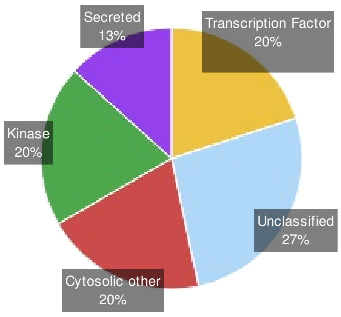

Target	Uniprot ID	Gene code	ChEMBL ID	Probability	# sim. cmpds (3D / 2D)	Target Class
Signal transducer and activator of transcription 3	P40763	STAT3	CHEMBL4026		0 / 4	Transcription Factor
Signal transducer and activator of transcription 1-alpha/beta *(by homology)*	P42224	STAT1	CHEMBL6101		0 / 4	Unclassified
Signal transducer and activator of transcription 2 *(by homology)*	P52630	STAT2			0 / 4	Transcription Factor
Signal transducer and activator of transcription 4 *(by homology)*	Q14765	STAT4			0 / 4	Transcription Factor
Bcl-2-like protein 1	Q07817	BCL2L1	CHEMBL4625		0 / 1	Cytosolic other
Apoptosis regulator Bcl-2 *(by homology)*	P10415	BCL2	CHEMBL4860		0 / 1	Cytosolic other
Bcl-2-like protein 2 *(by homology)*	Q92843	BCL2L2	CHEMBL4677		0 / 1	Cytosolic other
Muscleblind-like protein 1	Q9NR56	MBNL1	CHEMBL1293317		0 / 3	Unclassified
Muscleblind-like protein 2 *(by homology)*	Q5VZF2	MBNL2			0 / 3	Unclassified
Muscleblind-like protein 3 *(by homology)*	Q9NUK0	MBNL3			0 / 3	Unclassified
Vascular endothelial growth factor receptor 1	P17948	FLT1	CHEMBL1868		1 / 1	Tyr Kinase
Vascular endothelial growth factor receptor 2	P35968	KDR	CHEMBL279		1 / 1	Tyr Kinase
Vascular endothelial growth factor receptor 3 *(by homology)*	P35916	FLT4	CHEMBL1955		1 / 1	Tyr Kinase
Fibroblast growth factor 1	P05230	FGF1	CHEMBL2120		0 / 6	Secreted
Fibroblast growth factor 2	P09038	FGF2	CHEMBL3107		0 / 6	Secreted

SwissTargetPrediction report:

Reference:
Gfeller D., Michielin O. & Zoete V.
Shaping the interaction landscape of bioactive molecules, *Bioinformatics* (2013) 29:3073-3079.

Query Molecule

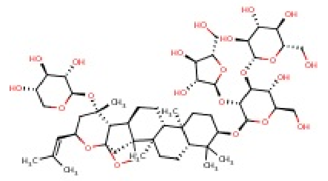

Frequency of Target Class

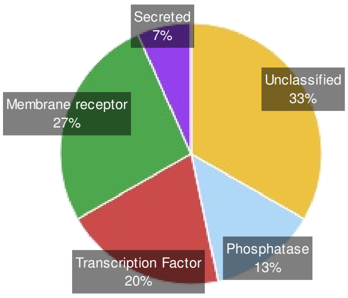

Target	Uniprot ID	Gene code	ChEMBL ID	Probability	# sim. cmpds (3D / 2D)	Target Class
Muscleblind-like protein 1	Q9NR56	MBNL1	CHEMBL1293317		0 / 2	Unclassified
Muscleblind-like protein 2 *(by homology)*	Q5VZF2	MBNL2			0 / 2	Unclassified
Muscleblind-like protein 3 *(by homology)*	Q9NUK0	MBNL3			0 / 2	Unclassified
Tyrosine-protein phosphatase non-receptor type 1	P18031	PTPN1	CHEMBL335		2 / 6	Tyr Phosphatase
Tyrosine-protein phosphatase non-receptor type 2 *(by homology)*	P17706	PTPN2	CHEMBL3807		2 / 6	Tyr Phosphatase
Signal transducer and activator of transcription 3	P40763	STAT3	CHEMBL4026		0 / 4	Transcription Factor
Signal transducer and activator of transcription 1-alpha/beta *(by homology)*	P42224	STAT1	CHEMBL6101		0 / 4	Unclassified
Signal transducer and activator of transcription 2 *(by homology)*	P52630	STAT2			0 / 4	Transcription Factor
Signal transducer and activator of transcription 4 *(by homology)*	Q14765	STAT4			0 / 4	Transcription Factor
Microtubule-associated protein tau	P10636	MAPT	CHEMBL1293224		0 / 12	Unclassified
Mu-type opioid receptor	P35372	OPRM1	CHEMBL233		1 / 6	Membrane receptor
Delta-type opioid receptor	P41143	OPRD1	CHEMBL236		1 / 6	Membrane receptor
Kappa-type opioid receptor	P41145	OPRK1	CHEMBL237		1 / 6	Membrane receptor
Nociceptin receptor *(by homology)*	P41146	OPRL1	CHEMBL2014		1 / 6	Membrane receptor
Fibroblast growth factor 1	P05230	FGF1	CHEMBL2120		0 / 6	Secreted

SwissTargetPrediction report:

Reference:
Gfeller D., Michielin O. & Zoete V.
Shaping the interaction landscape of bioactive molecules, *Bioinformatics* (2013) 29:3073-3079.

Query Molecule

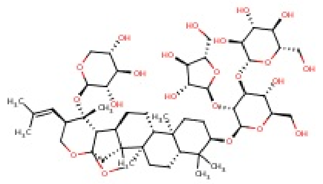

Frequency of Target Class

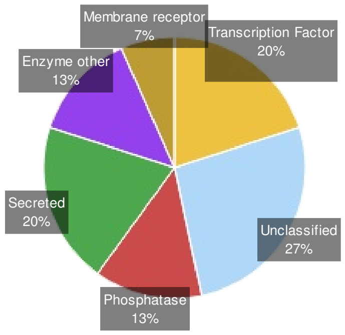

Target	Uniprot ID	Gene code	ChEMBL ID	Probability	# sim. cmpds (3D / 2D)	Target Class
Signal transducer and activator of transcription 3	P40763	STAT3	CHEMBL4026		0 / 4	Transcription Factor
Signal transducer and activator of transcription 1-alpha/beta *(by homology)*	P42224	STAT1	CHEMBL6101		0 / 4	Unclassified
Signal transducer and activator of transcription 2 *(by homology)*	P52630	STAT2			0 / 4	Transcription Factor
Signal transducer and activator of transcription 4 *(by homology)*	Q14765	STAT4			0 / 4	Transcription Factor
Tyrosine-protein phosphatase non-receptor type 1	P18031	PTPN1	CHEMBL335		2 / 2	Tyr Phosphatase
Tyrosine-protein phosphatase non-receptor type 2 *(by homology)*	P17706	PTPN2	CHEMBL3807		2 / 2	Tyr Phosphatase
Fibroblast growth factor 1	P05230	FGF1	CHEMBL2120		0 / 8	Secreted
Fibroblast growth factor 2	P09038	FGF2	CHEMBL3107		0 / 8	Secreted
Vascular endothelial growth factor A	P15692	VEGFA	CHEMBL1783		0 / 2	Secreted
Heparanase 8 kDa subunit	Q9Y251	HPSE	CHEMBL3921		0 / 4	Enzyme
Inactive heparanase-2 *(by homology)*	Q8WWQ2	HPSE2			0 / 4	Enzyme
Muscleblind-like protein 1	Q9NR56	MBNL1	CHEMBL1293317		0 / 3	Unclassified
Muscleblind-like protein 2 *(by homology)*	Q5VZF2	MBNL2			0 / 3	Unclassified
Muscleblind-like protein 3 *(by homology)*	Q9NUK0	MBNL3			0 / 3	Unclassified
Muscarinic acetylcholine receptor M5 *(by homology)*	P08912	CHRM5	CHEMBL2035		0 / 2	Membrane receptor

SwissTargetPrediction report:

Reference:
Gfeller D., Michielin O. & Zoete V.
Shaping the interaction landscape of bioactive molecules, *Bioinformatics* (2013) 29:3073-3079.

Query Molecule

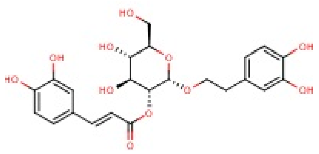

Frequency of Target Class

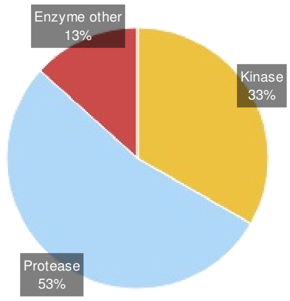

Target	Uniprot ID	Gene code	ChEMBL ID	Probability	# sim. cmpds (3D / 2D)	Target Class
Protein kinase C gamma type *(by homology)*	P05129	PRKCG	CHEMBL2938		10 / 43	Ser_Thr Kinase
Protein kinase C beta type *(by homology)*	P05771	PRKCB	CHEMBL3045		10 / 43	Ser_Thr Kinase
Protein kinase C alpha type	P17252	PRKCA	CHEMBL299		10 / 43	Ser_Thr Kinase
Protein kinase C theta type *(by homology)*	Q04759	PRKCQ	CHEMBL3920		10 / 45	Ser_Thr Kinase
Protein kinase C delta type regulatory subunit *(by homology)*	Q05655	PRKCD	CHEMBL2996		10 / 45	Ser_Thr Kinase
PEX	P08253	MMP2	CHEMBL333		17 / 6	Metallo Protease
67 kDa matrix metalloproteinase-9 *(by homology)*	P14780	MMP9	CHEMBL321		17 / 6	Metallo Protease
22 kDa interstitial collagenase	P03956	MMP1	CHEMBL332		17 / 5	Metallo Protease
Stromelysin-1 *(by homology)*	P08254	MMP3	CHEMBL283		17 / 5	Metallo Protease
Macrophage metalloelastase	P39900	MMP12	CHEMBL4393		14 / 5	Metallo Protease
Collagenase 3	P45452	MMP13	CHEMBL280		14 / 5	Metallo Protease
Stromelysin-2 *(by homology)*	P09238	MMP10	CHEMBL4270		17 / 5	Metallo Protease
Matrix metalloproteinase-27 *(by homology)*	Q9H306	MMP27			17 / 5	Metallo Protease
Aldo-keto reductase family 1 member B10	O60218	AKR1B10	CHEMBL5983		15 / 23	Enzyme
Aldose reductase	P15121	AKR1B1	CHEMBL1900		15 / 23	Enzyme

SwissTargetPrediction report:

Reference:
Gfeller D., Michielin O. & Zoete V.
Shaping the interaction landscape of bioactive molecules, *Bioinformatics* (2013) 29:3073-3079.

Query Molecule

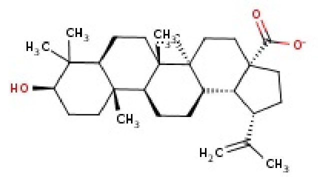

Frequency of Target Class

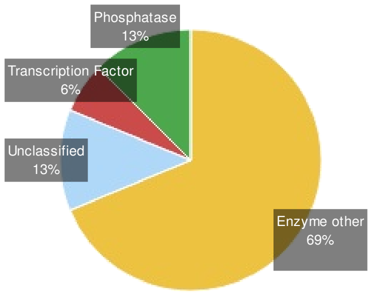

Target	Uniprot ID	Gene code	ChEMBL ID	Probability	# sim. cmpds (3D / 2D)	Target Class
Aldo-keto reductase family 1 member B10	O60218	AKR1B10	CHEMBL5983		4 / 8	Enzyme
DNA polymerase beta *(by homology)*	P06746	POLB	CHEMBL2392		7 / 11	Enzyme
Aldose reductase *(by homology)*	P15121	AKR1B1	CHEMBL1900		4 / 8	Enzyme
Aldo-keto reductase family 1 member B15 *(by homology)*	C9JRZ8	AKR1B15			4 / 8	Enzyme
Alcohol dehydrogenase [NADP(+)] *(by homology)*	P14550	AKR1A1	CHEMBL2246		4 / 8	Enzyme
1,5-anhydro-D-fructose reductase *(by homology)*	Q96JD6	AKR1E2			4 / 8	Enzyme
Complex	Q9UBE0/Q9UBT2	SAE1/UBA2	CHEMBL2095174		1 / 1	Unclassified /Enzyme
Microtubule-associated protein tau	P10636	MAPT	CHEMBL1293224		4 / 17	Unclassified
Corticosteroid 11-beta-dehydrogenase isozyme 1	P28845	HSD11B1	CHEMBL4235		20 / 21	Enzyme
Hydroxysteroid 11-beta-dehydrogenase 1-like protein *(by homology)*	Q7Z5J1	HSD11B1L			20 / 21	Enzyme
Bile acid receptor	Q96RI1	NR1H4	CHEMBL2047		22 / 13	Transcription Factor
M-phase inducer phosphatase 1	P30304	CDC25A	CHEMBL3775		3 / 19	Ser_Thr_Tyr Phosphatase
M-phase inducer phosphatase 2 *(by homology)*	P30305	CDC25B	CHEMBL4804		3 / 19	Ser_Thr_Tyr Phosphatase
DNA topoisomerase 2-alpha	P11388	TOP2A	CHEMBL1806		1 / 2	Enzyme
DNA topoisomerase 2-beta *(by homology)*	Q02880	TOP2B	CHEMBL3396		1 / 2	Enzyme

SwissTargetPrediction report:

Reference:
Gfeller D., Michielin O. & Zoete V.
Shaping the interaction landscape of bioactive molecules, *Bioinformatics* (2013) 29:3073-3079.

Query Molecule

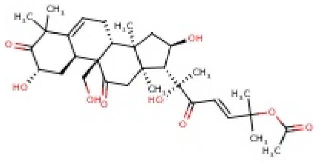

Frequency of Target Class

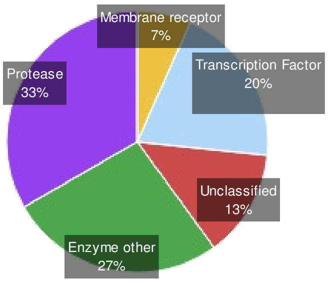

Target	Uniprot ID	Gene code	ChEMBL ID	Probability	# sim. cmpds (3D / 2D)	Target Class
Integrin alpha-L	P20701	ITGAL	CHEMBL1803		1 / 4	Membrane receptor
Signal transducer and activator of transcription 3	P40763	STAT3	CHEMBL4026		2 / 1	Transcription Factor
Signal transducer and activator of transcription 1-alpha/beta *(by homology)*	P42224	STAT1	CHEMBL6101		2 / 1	Unclassified
Signal transducer and activator of transcription 2 *(by homology)*	P52630	STAT2			2 / 1	Transcription Factor
Signal transducer and activator of transcription 4 *(by homology)*	Q14765	STAT4			2 / 1	Transcription Factor
DNA polymerase alpha catalytic subunit	P09884	POLA1	CHEMBL1828		2 / 10	Enzyme
Quinone oxidoreductase *(by homology)*	Q08257	CRYZ	CHEMBL6118		10 / 24	Enzyme
Microtubule-associated protein tau	P10636	MAPT	CHEMBL1293224		102 / 31	Unclassified
3-hydroxy-3-methylglutaryl-coenzyme A reductase *(by homology)*	P04035	HMGCR	CHEMBL402		27 / 75	Enzyme
Tubulin--tyrosine ligase	Q8NG68	TTL	CHEMBL5549		7 / 12	Enzyme
22 kDa interstitial collagenase	P03956	MMP1	CHEMBL332		118 / 3	Metallo Protease
Stromelysin-1	P08254	MMP3	CHEMBL283		118 / 3	Metallo Protease
Neutrophil collagenase *(by homology)*	P22894	MMP8	CHEMBL4588		94 / 3	Metallo Protease
Macrophage metalloelastase *(by homology)*	P39900	MMP12	CHEMBL4393		110 / 3	Metallo Protease
Collagenase 3 *(by homology)*	P45452	MMP13	CHEMBL280		110 / 3	Metallo Protease

SwissTargetPrediction report:

Reference:
Gfeller D., Michielin O. & Zoete V.
Shaping the interaction landscape of bioactive molecules, *Bioinformatics* (2013) 29:3073-3079.

Query Molecule

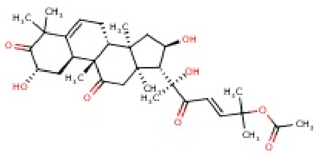

Frequency of Target Class

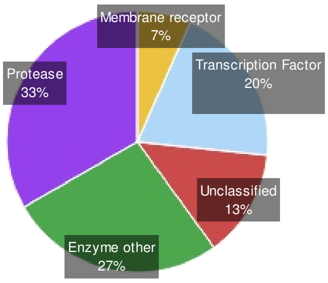

Target	Uniprot ID	Gene code	ChEMBL ID	Probability	# sim. cmpds (3D / 2D)	Target Class
Integrin alpha-L	P20701	ITGAL	CHEMBL1803		3 / 4	Membrane receptor
Signal transducer and activator of transcription 3	P40763	STAT3	CHEMBL4026		4 / 1	Transcription Factor
Signal transducer and activator of transcription 1-alpha/beta *(by homology)*	P42224	STAT1	CHEMBL6101		4 / 1	Unclassified
Signal transducer and activator of transcription 4 *(by homology)*	Q14765	STAT4			4 / 1	Transcription Factor
Signal transducer and activator of transcription 2 *(by homology)*	P52630	STAT2			3 / 1	Transcription Factor
Microtubule-associated protein tau	P10636	MAPT	CHEMBL1293224		234 / 32	Unclassified
DNA polymerase alpha catalytic subunit	P09884	POLA1	CHEMBL1828		3 / 10	Enzyme
Nitric oxide synthase, brain *(by homology)*	P29475	NOS1	CHEMBL3568		12 / 15	Enzyme
Nitric oxide synthase, inducible	P35228	NOS2	CHEMBL4481		12 / 15	Enzyme
Nitric oxide synthase, endothelial *(by homology)*	P29474	NOS3	CHEMBL4803		12 / 15	Enzyme
22 kDa interstitial collagenase	P03956	MMP1	CHEMBL332		280 / 3	Metallo Protease
Stromelysin-1	P08254	MMP3	CHEMBL283		280 / 3	Metallo Protease
Stromelysin-2 *(by homology)*	P09238	MMP10	CHEMBL4270		280 / 3	Metallo Protease
Neutrophil collagenase *(by homology)*	P22894	MMP8	CHEMBL4588		212 / 3	Metallo Protease
Macrophage metalloelastase *(by homology)*	P39900	MMP12	CHEMBL4393		264 / 3	Metallo Protease

SwissTargetPrediction report:

Reference:
Gfeller D., Michielin O. & Zoete V.
Shaping the interaction landscape of bioactive molecules, *Bioinformatics* (2013) 29:3073-3079.

Query Molecule

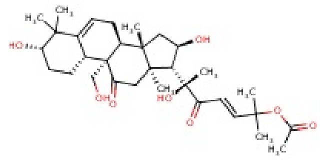

Frequency of Target Class

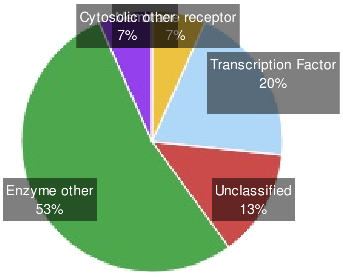

Target	Uniprot ID	Gene code	ChEMBL ID	Probability	# sim. cmpds (3D / 2D)	Target Class
Integrin alpha-L	P20701	ITGAL	CHEMBL1803		3 / 4	Membrane receptor
Signal transducer and activator of transcription 3	P40763	STAT3	CHEMBL4026		3 / 1	Transcription Factor
Signal transducer and activator of transcription 1-alpha/beta *(by homology)*	P42224	STAT1	CHEMBL6101		3 / 1	Unclassified
Signal transducer and activator of transcription 4 *(by homology)*	Q14765	STAT4			3 / 1	Transcription Factor
Signal transducer and activator of transcription 2 *(by homology)*	P52630	STAT2			2 / 1	Transcription Factor
Microtubule-associated protein tau	P10636	MAPT	CHEMBL1293224		70 / 32	Unclassified
DNA polymerase alpha catalytic subunit	P09884	POLA1	CHEMBL1828		2 / 10	Enzyme
Quinone oxidoreductase *(by homology)*	Q08257	CRYZ	CHEMBL6118		10 / 24	Enzyme
Corticosteroid 11-beta-dehydrogenase isozyme 1	P28845	HSD11B1	CHEMBL4235		64 / 21	Enzyme
Hydroxysteroid 11-beta-dehydrogenase 1-like protein *(by homology)*	Q7Z5J1	HSD11B1L			64 / 21	Enzyme
Nitric oxide synthase, inducible *(by homology)*	P35228	NOS2	CHEMBL4481		2 / 15	Enzyme
Nitric oxide synthase, endothelial *(by homology)*	P29474	NOS3	CHEMBL4803		2 / 15	Enzyme
Nitric oxide synthase, brain *(by homology)*	P29475	NOS1	CHEMBL3568		2 / 15	Enzyme
3-hydroxy-3-methylglutaryl-coenzyme A reductase *(by homology)*	P04035	HMGCR	CHEMBL402		32 / 79	Enzyme
Apoptosis regulator Bcl-2	P10415	BCL2	CHEMBL4860		7 / 2	Cytosolic other

SwissTargetPrediction report:

Reference:
Gfeller D., Michielin O. & Zoete V.
Shaping the interaction landscape of bioactive molecules, *Bioinformatics* (2013) 29:3073-3079.

Query Molecule

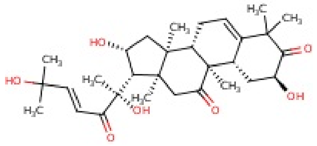

Frequency of Target Class

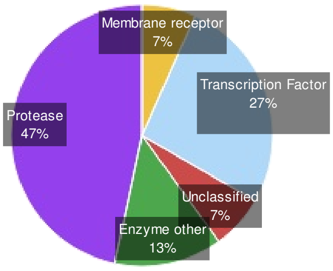

Target	Uniprot ID	Gene code	ChEMBL ID	Probability	# sim. cmpds (3D / 2D)	Target Class
Integrin alpha-L	P20701	ITGAL	CHEMBL1803		3 / 4	Membrane receptor
Signal transducer and activator of transcription 3	P40763	STAT3	CHEMBL4026		3 / 1	Transcription Factor
Signal transducer and activator of transcription 1-alpha/beta *(by homology)*	P42224	STAT1	CHEMBL6101		3 / 1	Unclassified
Signal transducer and activator of transcription 2 *(by homology)*	P52630	STAT2			3 / 1	Transcription Factor
Signal transducer and activator of transcription 4 *(by homology)*	Q14765	STAT4			3 / 1	Transcription Factor
Prostaglandin G/H synthase 1 *(by homology)*	P23219	PTGS1	CHEMBL221		18 / 6	Enzyme
Prostaglandin G/H synthase 2	P35354	PTGS2	CHEMBL230		18 / 6	Enzyme
22 kDa interstitial collagenase	P03956	MMP1	CHEMBL332		102 / 3	Metallo Protease
Stromelysin-1	P08254	MMP3	CHEMBL283		102 / 3	Metallo Protease
Neutrophil collagenase	P22894	MMP8	CHEMBL4588		88 / 3	Metallo Protease
Macrophage metalloelastase *(by homology)*	P39900	MMP12	CHEMBL4393		91 / 3	Metallo Protease
Collagenase 3 *(by homology)*	P45452	MMP13	CHEMBL280		91 / 3	Metallo Protease
Stromelysin-2 *(by homology)*	P09238	MMP10	CHEMBL4270		102 / 3	Metallo Protease
Matrix metalloproteinase-27 *(by homology)*	Q9H306	MMP27			102 / 3	Metallo Protease
Glucocorticoid receptor	P04150	NR3C1	CHEMBL2034		52 / 39	Transcription Factor

SwissTargetPrediction report:

Reference:
Gfeller D., Michielin O. & Zoete V.
Shaping the interaction landscape of bioactive molecules, *Bioinformatics* (2013) 29:3073-3079.

Query Molecule

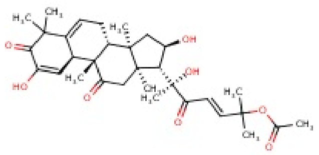

Frequency of Target Class

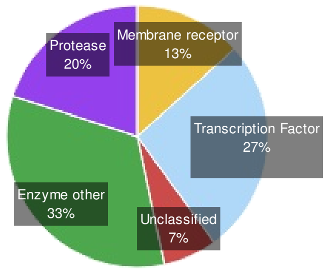

Target	Uniprot ID	Gene code	ChEMBL ID	Probability	# sim. cmpds (3D / 2D)	Target Class
Integrin alpha-L	P20701	ITGAL	CHEMBL1803		2 / 4	Membrane receptor
Signal transducer and activator of transcription 3	P40763	STAT3	CHEMBL4026		2 / 1	Transcription Factor
Signal transducer and activator of transcription 1-alpha/beta *(by homology)*	P42224	STAT1	CHEMBL6101		2 / 1	Unclassified
Signal transducer and activator of transcription 4 *(by homology)*	Q14765	STAT4			2 / 1	Transcription Factor
Signal transducer and activator of transcription 2 *(by homology)*	P52630	STAT2			1 / 1	Transcription Factor
Quinone oxidoreductase *(by homology)*	Q08257	CRYZ	CHEMBL6118		4 / 24	Enzyme
DNA polymerase alpha catalytic subunit	P09884	POLA1	CHEMBL1828		1 / 7	Enzyme
Adenosine receptor A1	P30542	ADORA1	CHEMBL226		41 / 1	Membrane receptor
Nuclear receptor subfamily 1 group I member 2	O75469	NR1I2	CHEMBL3401		5 / 1	Transcription Factor
Prostaglandin G/H synthase 1	P23219	PTGS1	CHEMBL221		3 / 8	Enzyme
Prostaglandin G/H synthase 2	P35354	PTGS2	CHEMBL230		3 / 8	Enzyme
Cytochrome P450 19A1	P11511	CYP19A1	CHEMBL1978		1 / 71	Enzyme
22 kDa interstitial collagenase	P03956	MMP1	CHEMBL332		94 / 2	Metallo Protease
Stromelysin-1	P08254	MMP3	CHEMBL283		94 / 2	Metallo Protease
Neutrophil collagenase *(by homology)*	P22894	MMP8	CHEMBL4588		86 / 2	Metallo Protease

SwissTargetPrediction report:

Reference:
Gfeller D., Michielin O. & Zoete V.
Shaping the interaction landscape of bioactive molecules, *Bioinformatics* (2013) 29:3073-3079.

Query Molecule

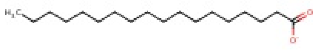

Frequency of Target Class

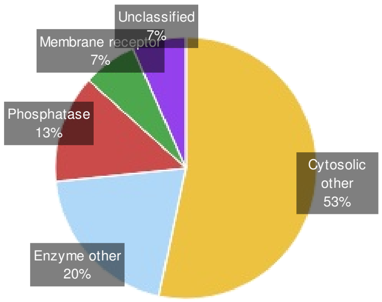

Target	Uniprot ID	Gene code	ChEMBL ID	Probability	# sim. cmpds (3D / 2D)	Target Class
Fatty acid-binding protein, heart	P05413	FABP3	CHEMBL3344		9 / 3	Cytosolic other
Fatty acid-binding protein, adipocyte	P15090	FABP4	CHEMBL2083		9 / 3	Cytosolic other
Fatty acid-binding protein, epidermal	Q01469	FABP5	CHEMBL3674		9 / 3	Cytosolic other
Fatty acid-binding protein 12 *(by homology)*	A6NFH5	FABP12			9 / 3	Cytosolic other
Fatty acid-binding protein, brain *(by homology)*	O15540	FABP7			9 / 3	Cytosolic other
Myelin P2 protein *(by homology)*	P02689	PMP2			9 / 3	Cytosolic other
Fatty acid-binding protein 9 *(by homology)*	Q0Z7S8	FABP9			9 / 3	Cytosolic other
Fatty acid-binding protein, intestinal	P12104	FABP2	CHEMBL4879		1 / 1	Cytosolic other
Tyrosyl-DNA phosphodiesterase 1	Q9NUW8	TDP1	CHEMBL1075138		6 / 13	Enzyme
M-phase inducer phosphatase 1	P30304	CDC25A	CHEMBL3775		12 / 16	Ser_Thr_Tyr Phosphatase
M-phase inducer phosphatase 2 *(by homology)*	P30305	CDC25B	CHEMBL4804		12 / 16	Ser_Thr_Tyr Phosphatase
Corticosteroid 11-beta-dehydrogenase isozyme 1	P28845	HSD11B1	CHEMBL4235		16 / 10	Enzyme
Hydroxysteroid 11-beta-dehydrogenase 1-like protein *(by homology)*	Q7Z5J1	HSD11B1L			16 / 10	Enzyme
Prostaglandin E2 receptor EP2 subtype	P43116	PTGER2	CHEMBL1881		93 / 5	Membrane receptor
Microtubule-associated protein tau	P10636	MAPT	CHEMBL1293224		3 / 8	Unclassified

SwissTargetPrediction report:

Reference:
Gfeller D., Michielin O. & Zoete V.
Shaping the interaction landscape of bioactive molecules, *Bioinformatics* (2013) 29:3073-3079.

Query Molecule

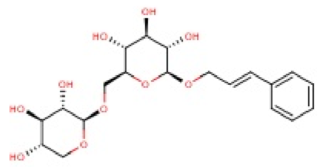

Frequency of Target Class

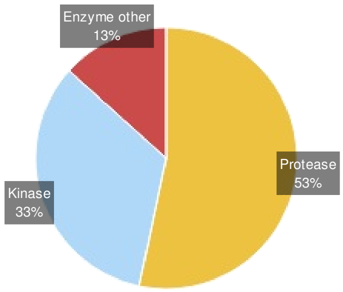

Target	Uniprot ID	Gene code	ChEMBL ID	Probability	# sim. cmpds (3D / 2D)	Target Class
22 kDa interstitial collagenase	P03956	MMP1	CHEMBL332		1 / 2	Metallo Protease
Macrophage metalloelastase	P39900	MMP12	CHEMBL4393		1 / 2	Metallo Protease
Collagenase 3	P45452	MMP13	CHEMBL280		1 / 2	Metallo Protease
Stromelysin-1 *(by homology)*	P08254	MMP3	CHEMBL283		1 / 2	Metallo Protease
Stromelysin-2 *(by homology)*	P09238	MMP10	CHEMBL4270		1 / 2	Metallo Protease
Matrix metalloproteinase-27 *(by homology)*	Q9H306	MMP27			1 / 2	Metallo Protease
PEX	P08253	MMP2	CHEMBL333		1 / 2	Metallo Protease
67 kDa matrix metalloproteinase-9 *(by homology)*	P14780	MMP9	CHEMBL321		1 / 2	Metallo Protease
Protein kinase C gamma type *(by homology)*	P05129	PRKCG	CHEMBL2938		2 / 22	Ser_Thr Kinase
Protein kinase C beta type *(by homology)*	P05771	PRKCB	CHEMBL3045		2 / 22	Ser_Thr Kinase
Protein kinase C alpha type	P17252	PRKCA	CHEMBL299		2 / 22	Ser_Thr Kinase
Protein kinase C theta type *(by homology)*	Q04759	PRKCQ	CHEMBL3920		2 / 23	Ser_Thr Kinase
Protein kinase C delta type regulatory subunit *(by homology)*	Q05655	PRKCD	CHEMBL2996		2 / 23	Ser_Thr Kinase
Squalene synthase	P37268	FDFT1	CHEMBL3338		117 / 162	Enzyme
Matrix metalloproteinase-20 *(by homology)*	O60882	MMP20	CHEMBL1938226		1 / 1	Enzyme

SwissTargetPrediction report:

Reference:
Gfeller D., Michielin O. & Zoete V.
Shaping the interaction landscape of bioactive molecules, *Bioinformatics* (2013) 29:3073-3079.

Query Molecule

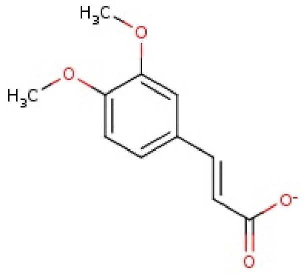

Frequency of Target Class

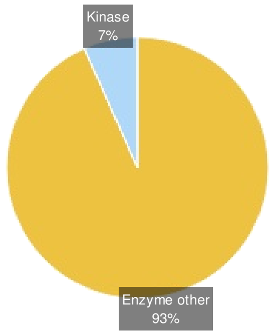

Target	Uniprot ID	Gene code	ChEMBL ID	Probability	# sim. cmpds (3D / 2D)	Target Class
Carbonic anhydrase 1	P00915	CA1	CHEMBL261		31 / 13	Enzyme
Carbonic anhydrase 2	P00918	CA2	CHEMBL205		31 / 13	Enzyme
Carbonic anhydrase 3 *(by homology)*	P07451	CA3	CHEMBL2885		31 / 13	Enzyme
Carbonic anhydrase 5A, mitochondrial	P35218	CA5A	CHEMBL4789		31 / 13	Enzyme
Carbonic anhydrase 7	P43166	CA7	CHEMBL2326		31 / 13	Enzyme
Carbonic anhydrase 13 *(by homology)*	Q8N1Q1	CA13	CHEMBL3912		31 / 13	Enzyme
Carbonic anhydrase 5B, mitochondrial *(by homology)*	Q9Y2D0	CA5B	CHEMBL3969		31 / 13	Enzyme
Carbonic anhydrase 12	O43570	CA12	CHEMBL3242		10 / 10	Enzyme
Carbonic anhydrase 9	Q16790	CA9	CHEMBL3594		10 / 10	Enzyme
Carbonic anhydrase 14	Q9ULX7	CA14	CHEMBL3510		10 / 10	Enzyme
Dual specificity tyrosine-phosphorylation-regulated kinase 1A *(by homology)*	Q13627	DYRK1A	CHEMBL2292		14 / 14	Ser_Thr_Tyr Kinase
Amine oxidase [flavin-containing] A	P21397	MAOA	CHEMBL1951		3 / 10	Enzyme
Amine oxidase [flavin-containing] B	P27338	MAOB	CHEMBL2039		3 / 11	Enzyme
Prostaglandin G/H synthase 1	P23219	PTGS1	CHEMBL221		54 / 20	Enzyme
Prostaglandin G/H synthase 2	P35354	PTGS2	CHEMBL230		54 / 20	Enzyme

SwissTargetPrediction report:

Reference:
Gfeller D., Michielin O. & Zoete V.
Shaping the interaction landscape of bioactive molecules, *Bioinformatics* (2013) 29:3073-3079.

Query Molecule

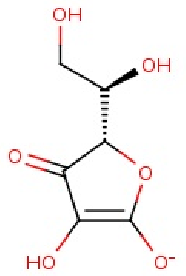

Frequency of Target Class

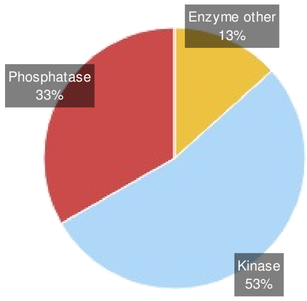

Target	Uniprot ID	Gene code	ChEMBL ID	Probability	# sim. cmpds (3D / 2D)	Target Class
Tyrosyl-DNA phosphodiesterase 1	Q9NUW8	TDP1	CHEMBL1075138		0 / 2	Enzyme
Tubulin--tyrosine ligase	Q8NG68	TTL	CHEMBL5549		0 / 12	Enzyme
Protein kinase C alpha type	P17252	PRKCA	CHEMBL299		0 / 141	Ser_Thr Kinase
Protein kinase C delta type regulatory subunit *(by homology)*	Q05655	PRKCD	CHEMBL2996		0 / 143	Ser_Thr Kinase
Protein kinase C theta type *(by homology)*	Q04759	PRKCQ	CHEMBL3920		0 / 143	Ser_Thr Kinase
Protein kinase C gamma type *(by homology)*	P05129	PRKCG	CHEMBL2938		0 / 141	Ser_Thr Kinase
Protein kinase C beta type *(by homology)*	P05771	PRKCB	CHEMBL3045		0 / 141	Ser_Thr Kinase
Dual specificity protein phosphatase 3	P51452	DUSP3	CHEMBL2635		0 / 1	Ser_Thr_Tyr Phosphatase
Inactive dual specificity phosphatase 27 *(by homology)*	Q5VZP5	DUSP27			0 / 1	Ser_Thr_Tyr Phosphatase
Dual specificity phosphatase DUPD1 *(by homology)*	Q68J44	DUPD1			0 / 1	Ser_Thr_Tyr Phosphatase
Dual specificity protein phosphatase 13 isoform MDSP *(by homology)*	Q6B8I1	DUSP13			0 / 1	Ser_Thr_Tyr Phosphatase
Dual specificity protein phosphatase 26 *(by homology)*	Q9BV47	DUSP26			0 / 1	Ser_Thr_Tyr Phosphatase
Ribosomal protein S6 kinase alpha-5	O75582	RPS6KA5	CHEMBL4237		0 / 3	Ser_Thr Kinase
Ribosomal protein S6 kinase alpha-4 *(by homology)*	O75676	RPS6KA4	CHEMBL3125		0 / 3	Ser_Thr Kinase
Ribosomal protein S6 kinase alpha-3 *(by homology)*	P51812	RPS6KA3	CHEMBL2345		0 / 3	Ser_Thr Kinase

SwissTargetPrediction report:

Reference:
Gfeller D., Michielin O. & Zoete V.
Shaping the interaction landscape of bioactive molecules, *Bioinformatics* (2013) 29:3073-3079.

Query Molecule

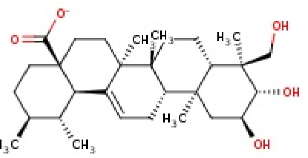

Frequency of Target Class

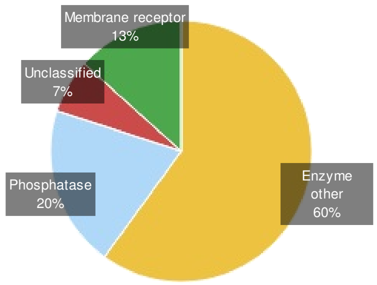

Target	Uniprot ID	Gene code	ChEMBL ID	Probability	# sim. cmpds (3D / 2D)	Target Class
Aldo-keto reductase family 1 member B10	O60218	AKR1B10	CHEMBL5983		6 / 7	Enzyme
Aldose reductase *(by homology)*	P15121	AKR1B1	CHEMBL1900		6 / 7	Enzyme
Aldo-keto reductase family 1 member B15 *(by homology)*	C9JRZ8	AKR1B15			6 / 7	Enzyme
Alcohol dehydrogenase [NADP(+)] *(by homology)*	P14550	AKR1A1	CHEMBL2246		6 / 7	Enzyme
1,5-anhydro-D-fructose reductase *(by homology)*	Q96JD6	AKR1E2			6 / 7	Enzyme
Tyrosine-protein phosphatase non-receptor type 2 *(by homology)*	P17706	PTPN2	CHEMBL3807		16 / 50	Tyr Phosphatase
Tyrosine-protein phosphatase non-receptor type 1	P18031	PTPN1	CHEMBL335		16 / 50	Tyr Phosphatase
Corticosteroid 11-beta-dehydrogenase isozyme 1	P28845	HSD11B1	CHEMBL4235		6 / 23	Enzyme
Hydroxysteroid 11-beta-dehydrogenase 1-like protein *(by homology)*	Q7Z5J1	HSD11B1L			6 / 23	Enzyme
Tyrosyl-DNA phosphodiesterase 1	Q9NUW8	TDP1	CHEMBL1075138		5 / 15	Enzyme
Microtubule-associated protein tau	P10636	MAPT	CHEMBL1293224		10 / 19	Unclassified
DNA polymerase beta	P06746	POLB	CHEMBL2392		6 / 17	Enzyme
Receptor-type tyrosine-protein phosphatase F	P10586	PTPRF	CHEMBL3521		1 / 1	Membrane receptor
Low molecular weight phosphotyrosine protein phosphatase	P24666	ACP1	CHEMBL4903		1 / 1	Tyr Phosphatase
Receptor-type tyrosine-protein phosphatase delta *(by homology)*	P23468	PTPRD			1 / 1	Membrane receptor

SwissTargetPrediction report:

Reference:
Gfeller D., Michielin O. & Zoete V.
Shaping the interaction landscape of bioactive molecules, *Bioinformatics* (2013) 29:3073-3079.

Query Molecule

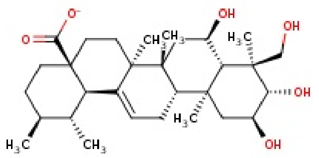

Frequency of Target Class

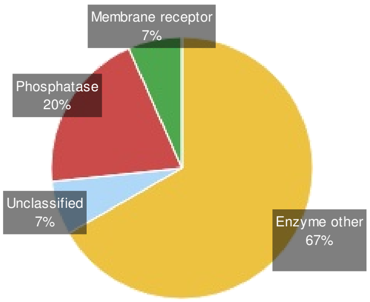

Target	Uniprot ID	Gene code	ChEMBL ID	Probability	# sim. cmpds (3D / 2D)	Target Class
Aldo-keto reductase family 1 member B10	O60218	AKR1B10	CHEMBL5983		7 / 6	Enzyme
Aldose reductase *(by homology)*	P15121	AKR1B1	CHEMBL1900		7 / 6	Enzyme
Aldo-keto reductase family 1 member B15 *(by homology)*	C9JRZ8	AKR1B15			7 / 6	Enzyme
Alcohol dehydrogenase [NADP(+)] *(by homology)*	P14550	AKR1A1	CHEMBL2246		6 / 6	Enzyme
1,5-anhydro-D-fructose reductase *(by homology)*	Q96JD6	AKR1E2			6 / 6	Enzyme
Microtubule-associated protein tau	P10636	MAPT	CHEMBL1293224		10 / 22	Unclassified
Tyrosyl-DNA phosphodiesterase 1	Q9NUW8	TDP1	CHEMBL1075138		3 / 12	Enzyme
Tyrosine-protein phosphatase non-receptor type 2 *(by homology)*	P17706	PTPN2	CHEMBL3807		14 / 50	Tyr Phosphatase
Tyrosine-protein phosphatase non-receptor type 1	P18031	PTPN1	CHEMBL335		14 / 50	Tyr Phosphatase
Corticosteroid 11-beta-dehydrogenase isozyme 1	P28845	HSD11B1	CHEMBL4235		6 / 23	Enzyme
Hydroxysteroid 11-beta-dehydrogenase 1-like protein *(by homology)*	Q7Z5J1	HSD11B1L			6 / 23	Enzyme
Phospholipase A2	P04054	PLA2G1B	CHEMBL4426		1 / 1	Enzyme
DNA polymerase beta	P06746	POLB	CHEMBL2392		6 / 16	Enzyme
Receptor-type tyrosine-protein phosphatase F	P10586	PTPRF	CHEMBL3521		2 / 1	Membrane receptor
Low molecular weight phosphotyrosine protein phosphatase	P24666	ACP1	CHEMBL4903		1 / 1	Tyr Phosphatase

SwissTargetPrediction report:

Reference:
Gfeller D., Michielin O. & Zoete V.
Shaping the interaction landscape of bioactive molecules, *Bioinformatics* (2013) 29:3073-3079.

Query Molecule

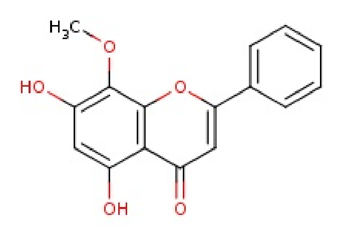

Frequency of Target Class

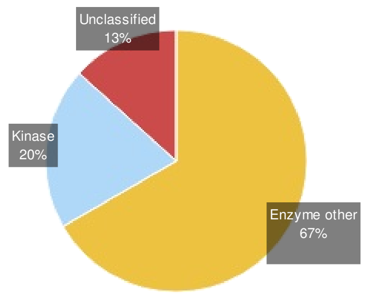

Target	Uniprot ID	Gene code	ChEMBL ID	Probability	# sim. cmpds (3D / 2D)	Target Class
Prostaglandin G/H synthase 1 *(by homology)*	P23219	PTGS1	CHEMBL221		14 / 37	Enzyme
Nitric oxide synthase, inducible *(by homology)*	P35228	NOS2	CHEMBL4481		6 / 3	Enzyme
Prostaglandin G/H synthase 2 *(by homology)*	P35354	PTGS2	CHEMBL230		14 / 37	Enzyme
Nitric oxide synthase, endothelial *(by homology)*	P29474	NOS3	CHEMBL4803		6 / 3	Enzyme
Nitric oxide synthase, brain *(by homology)*	P29475	NOS1	CHEMBL3568		6 / 3	Enzyme
Aldose reductase *(by homology)*	P15121	AKR1B1	CHEMBL1900		20 / 68	Enzyme
Aldo-keto reductase family 1 member B15 *(by homology)*	C9JRZ8	AKR1B15			20 / 68	Enzyme
Aldo-keto reductase family 1 member B10 *(by homology)*	O60218	AKR1B10	CHEMBL5983		20 / 68	Enzyme
Inhibitor of nuclear factor kappa-B kinase subunit beta	O14920	IKBKB	CHEMBL1991		14 / 1	Ser_Thr Kinase
Inhibitor of nuclear factor kappa-B kinase subunit alpha *(by homology)*	O15111	CHUK	CHEMBL3476		14 / 1	Ser_Thr Kinase
Dual specificity tyrosine-phosphorylation-regulated kinase 1A *(by homology)*	Q13627	DYRK1A	CHEMBL2292		40 / 21	Ser_Thr_Tyr Kinase
Microtubule-associated protein tau	P10636	MAPT	CHEMBL1293224		254 / 83	Unclassified
ATP-binding cassette sub-family G member 2	Q9UNQ0	ABCG2	CHEMBL5393		8 / 20	Unclassified
Tyrosyl-DNA phosphodiesterase 1	Q9NUW8	TDP1	CHEMBL1075138		70 / 24	Enzyme
Arachidonate 5-lipoxygenase	P09917	ALOX5	CHEMBL215		27 / 53	Enzyme

SwissTargetPrediction report:

Reference:
Gfeller D., Michielin O. & Zoete V.
Shaping the interaction landscape of bioactive molecules, *Bioinformatics* (2013) 29:3073-3079.

Query Molecule

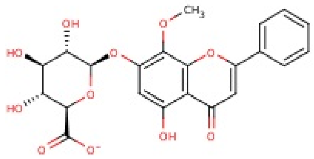

Frequency of Target Class

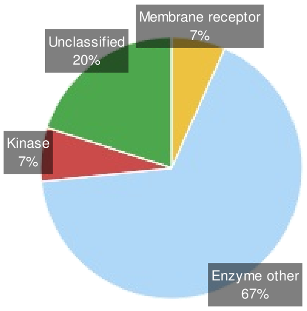

Target	Uniprot ID	Gene code	ChEMBL ID	Probability	# sim. cmpds (3D / 2D)	Target Class
Adenosine receptor A1 *(by homology)*	P30542	ADORA1	CHEMBL226		20 / 10	Membrane receptor
Aldose reductase *(by homology)*	P15121	AKR1B1	CHEMBL1900		1 / 56	Enzyme
Aldo-keto reductase family 1 member B15 *(by homology)*	C9JRZ8	AKR1B15			1 / 56	Enzyme
Aldo-keto reductase family 1 member B10 *(by homology)*	O60218	AKR1B10	CHEMBL5983		1 / 56	Enzyme
Tyrosyl-DNA phosphodiesterase 1	Q9NUW8	TDP1	CHEMBL1075138		3 / 11	Enzyme
Dual specificity tyrosine-phosphorylation-regulated kinase 1A *(by homology)*	Q13627	DYRK1A	CHEMBL2292		1 / 12	Ser_Thr_Tyr Kinase
Muscleblind-like protein 1	Q9NR56	MBNL1	CHEMBL1293317		1 / 4	Unclassified
Muscleblind-like protein 2 *(by homology)*	Q5VZF2	MBNL2			1 / 4	Unclassified
Muscleblind-like protein 3 *(by homology)*	Q9NUK0	MBNL3			1 / 4	Unclassified
Xanthine dehydrogenase/oxidase	P47989	XDH	CHEMBL1929		0 / 9	Enzyme
Aldehyde oxidase *(by homology)*	Q06278	AOX1	CHEMBL3257		0 / 9	Enzyme
Lysine-specific demethylase 4A	O75164	KDM4A	CHEMBL5896		0 / 3	Enzyme
Lysine-specific demethylase 4B *(by homology)*	O94953	KDM4B			0 / 3	Enzyme
Lysine-specific demethylase 4C *(by homology)*	Q9H3R0	KDM4C	CHEMBL6175		0 / 3	Enzyme
Prostaglandin G/H synthase 2	P35354	PTGS2	CHEMBL230		0 / 5	Enzyme

SwissTargetPrediction report:

Reference:
Gfeller D., Michielin O. & Zoete V.
Shaping the interaction landscape of bioactive molecules, *Bioinformatics* (2013) 29:3073-3079.

Query Molecule

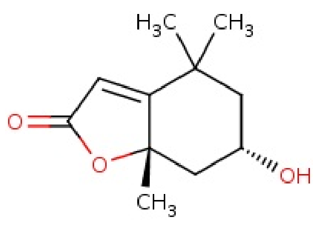

Frequency of Target Class

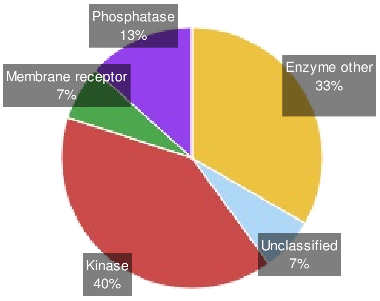

Target	Uniprot ID	Gene code	ChEMBL ID	Probability	# sim. cmpds (3D / 2D)	Target Class
Tyrosyl-DNA phosphodiesterase 1	Q9NUW8	TDP1	CHEMBL1075138		24 / 5	Enzyme
Microtubule-associated protein tau	P10636	MAPT	CHEMBL1293224		44 / 21	Unclassified
Quinone oxidoreductase *(by homology)*	Q08257	CRYZ	CHEMBL6118		1 / 21	Enzyme
Ribosomal protein S6 kinase alpha-5	O75582	RPS6KA5	CHEMBL4237		1 / 4	Ser_Thr Kinase
Ribosomal protein S6 kinase alpha-4 *(by homology)*	O75676	RPS6KA4	CHEMBL3125		1 / 4	Ser_Thr Kinase
Ribosomal protein S6 kinase alpha-3 *(by homology)*	P51812	RPS6KA3	CHEMBL2345		1 / 4	Ser_Thr Kinase
Ribosomal protein S6 kinase alpha-2 *(by homology)*	Q15349	RPS6KA2	CHEMBL3906		1 / 4	Ser_Thr Kinase
Ribosomal protein S6 kinase alpha-1 *(by homology)*	Q15418	RPS6KA1	CHEMBL2553		1 / 4	Ser_Thr Kinase
Ribosomal protein S6 kinase alpha-6 *(by homology)*	Q9UK32	RPS6KA6	CHEMBL4924		1 / 4	Ser_Thr Kinase
Prostaglandin G/H synthase 1 *(by homology)*	P23219	PTGS1	CHEMBL221		3 / 11	Enzyme
Prostaglandin G/H synthase 2	P35354	PTGS2	CHEMBL230		3 / 11	Enzyme
Platelet-activating factor receptor	P25105	PTAFR	CHEMBL250		14 / 19	Membrane receptor
M-phase inducer phosphatase 1	P30304	CDC25A	CHEMBL3775		1 / 17	Ser_Thr_Tyr Phosphatase
M-phase inducer phosphatase 2	P30305	CDC25B	CHEMBL4804		1 / 17	Ser_Thr_Tyr Phosphatase
Cytochrome P450 19A1	P11511	CYP19A1	CHEMBL1978		23 / 22	Enzyme

SwissTargetPrediction report:

Reference:
Gfeller D., Michielin O. & Zoete V.
Shaping the interaction landscape of bioactive molecules, *Bioinformatics* (2013) 29:3073-3079.

Query Molecule

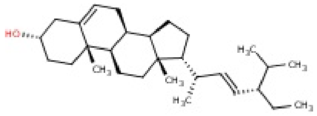

Frequency of Target Class

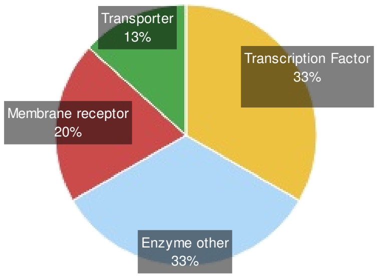

Target	Uniprot ID	Gene code	ChEMBL ID	Probability	# sim. cmpds (3D / 2D)	Target Class
Androgen receptor	P10275	AR	CHEMBL1871		13 / 90	Transcription Factor
Tyrosyl-DNA phosphodiesterase 1	Q9NUW8	TDP1	CHEMBL1075138		5 / 14	Enzyme
Cytochrome P450 19A1	P11511	CYP19A1	CHEMBL1978		4 / 135	Enzyme
3-hydroxy-3-methylglutaryl-coenzyme A reductase	P04035	HMGCR	CHEMBL402		24 / 33	Enzyme
Lanosterol 14-alpha demethylase *(by homology)*	Q16850	CYP51A1	CHEMBL3849		2 / 2	Enzyme
Oxysterols receptor LXR-beta *(by homology)*	P55055	NR1H2	CHEMBL4093		8 / 23	Transcription Factor
Oxysterols receptor LXR-alpha	Q13133	NR1H3	CHEMBL2808		8 / 23	Transcription Factor
Low-density lipoprotein receptor	P01130	LDLR	CHEMBL3311		10 / 10	Membrane receptor
Very low-density lipoprotein receptor *(by homology)*	P98155	VLDLR			10 / 10	Membrane receptor
Low-density lipoprotein receptor-related protein 8 *(by homology)*	Q14114	LRP8			10 / 10	Membrane receptor
Steroid 17-alpha-hydroxylase/17,20 lyase	P05093	CYP17A1	CHEMBL3522		21 / 56	Enzyme
Estrogen receptor	P03372	ESR1	CHEMBL206		1 / 52	Transcription Factor
Estrogen receptor beta	Q92731	ESR2	CHEMBL242		1 / 52	Transcription Factor
Sodium-dependent noradrenaline transporter	P23975	SLC6A2	CHEMBL222		37 / 6	Transporter
Sodium-dependent dopamine transporter *(by homology)*	Q01959	SLC6A3	CHEMBL238		37 / 6	Transporter

SwissTargetPrediction report:

Reference:
Gfeller D., Michielin O. & Zoete V.
Shaping the interaction landscape of bioactive molecules, *Bioinformatics* (2013) 29:3073-3079.

Query Molecule

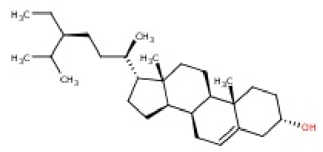

Frequency of Target Class

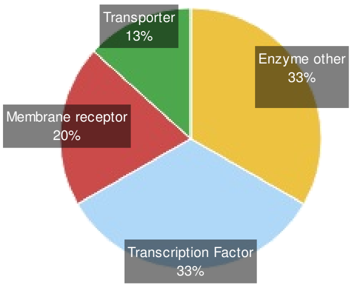

Target	Uniprot ID	Gene code	ChEMBL ID	Probability	# sim. cmpds (3D / 2D)	Target Class
Tyrosyl-DNA phosphodiesterase 1	Q9NUW8	TDP1	CHEMBL1075138		4 / 14	Enzyme
Androgen receptor	P10275	AR	CHEMBL1871		10 / 90	Transcription Factor
3-hydroxy-3-methylglutaryl-coenzyme A reductase	P04035	HMGCR	CHEMBL402		16 / 33	Enzyme
Lanosterol 14-alpha demethylase *(by homology)*	Q16850	CYP51A1	CHEMBL3849		2 / 2	Enzyme
Oxysterols receptor LXR-beta *(by homology)*	P55055	NR1H2	CHEMBL4093		13 / 23	Transcription Factor
Oxysterols receptor LXR-alpha	Q13133	NR1H3	CHEMBL2808		13 / 23	Transcription Factor
Cytochrome P450 19A1	P11511	CYP19A1	CHEMBL1978		5 / 135	Enzyme
Steroid 17-alpha-hydroxylase/17,20 lyase	P05093	CYP17A1	CHEMBL3522		17 / 56	Enzyme
Low-density lipoprotein receptor	P01130	LDLR	CHEMBL3311		10 / 10	Membrane receptor
Very low-density lipoprotein receptor *(by homology)*	P98155	VLDLR			10 / 10	Membrane receptor
Low-density lipoprotein receptor-related protein 8 *(by homology)*	Q14114	LRP8			10 / 10	Membrane receptor
Estrogen receptor	P03372	ESR1	CHEMBL206		3 / 52	Transcription Factor
Estrogen receptor beta	Q92731	ESR2	CHEMBL242		3 / 52	Transcription Factor
Sodium-dependent noradrenaline transporter	P23975	SLC6A2	CHEMBL222		38 / 6	Transporter
Sodium-dependent serotonin transporter	P31645	SLC6A4	CHEMBL228		40 / 6	Transporter

SwissTargetPrediction report:

Reference:
Gfeller D., Michielin O. & Zoete V.
Shaping the interaction landscape of bioactive molecules, *Bioinformatics* (2013) 29:3073-3079.

Query Molecule

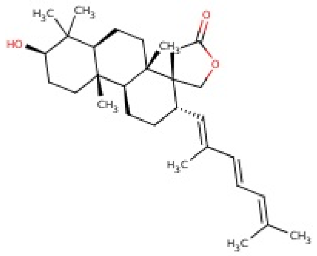

Frequency of Target Class

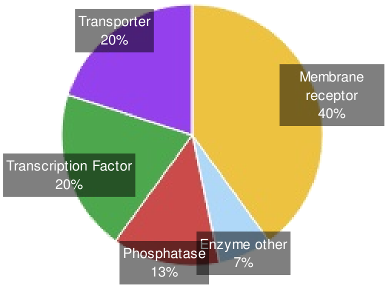

Target	Uniprot ID	Gene code	ChEMBL ID	Probability	# sim. cmpds (3D / 2D)	Target Class
D(2) dopamine receptor	P14416	DRD2	CHEMBL217		11 / 1	Membrane receptor
D(3) dopamine receptor	P35462	DRD3	CHEMBL234		8 / 1	Membrane receptor
3-hydroxy-3-methylglutaryl-coenzyme A reductase	P04035	HMGCR	CHEMBL402		7 / 98	Enzyme
5-hydroxytryptamine receptor 1A	P08908	HTR1A	CHEMBL214		7 / 1	Membrane receptor
5-hydroxytryptamine receptor 1B *(by homology)*	P28222	HTR1B	CHEMBL1898		7 / 1	Membrane receptor
Tyrosine-protein phosphatase non-receptor type 2 *(by homology)*	P17706	PTPN2	CHEMBL3807		5 / 48	Tyr Phosphatase
Tyrosine-protein phosphatase non-receptor type 1	P18031	PTPN1	CHEMBL335		5 / 48	Tyr Phosphatase
Glucocorticoid receptor	P04150	NR3C1	CHEMBL2034		7 / 18	Transcription Factor
Mineralocorticoid receptor *(by homology)*	P08235	NR3C2	CHEMBL1994		7 / 20	Transcription Factor
Androgen receptor *(by homology)*	P10275	AR	CHEMBL1871		17 / 29	Transcription Factor
Sodium-dependent noradrenaline transporter	P23975	SLC6A2	CHEMBL222		8 / 2	Transporter
Substance-P receptor *(by homology)*	P25103	TACR1	CHEMBL249		29 / 1	Membrane receptor
Sodium-dependent serotonin transporter	P31645	SLC6A4	CHEMBL228		11 / 2	Transporter
Sodium-dependent dopamine transporter	Q01959	SLC6A3	CHEMBL238		8 / 2	Transporter
Neuromedin-K receptor *(by homology)*	P29371	TACR3	CHEMBL4429		29 / 1	Membrane receptor

SwissTargetPrediction report:

Reference:
Gfeller D., Michielin O. & Zoete V.
Shaping the interaction landscape of bioactive molecules, *Bioinformatics* (2013) 29:3073-3079.

Query Molecule

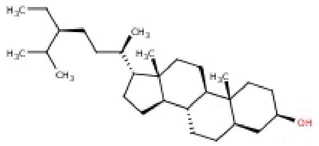

Frequency of Target Class

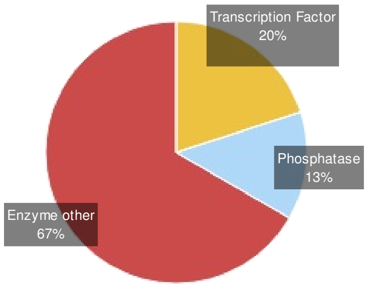

Target	Uniprot ID	Gene code	ChEMBL ID	Probability	# sim. cmpds (3D / 2D)	Target Class
Androgen receptor	P10275	AR	CHEMBL1871		11 / 14	Transcription Factor
M-phase inducer phosphatase 1	P30304	CDC25A	CHEMBL3775		5 / 8	Ser_Thr_Tyr Phosphatase
M-phase inducer phosphatase 2	P30305	CDC25B	CHEMBL4804		5 / 8	Ser_Thr_Tyr Phosphatase
UDP-glucuronosyltransferase 1-4	P22310	UGT1A4	CHEMBL3619		1 / 1	Enzyme
UDP-glucuronosyltransferase 1-9 *(by homology)*	O60656	UGT1A9	CHEMBL1743319		1 / 1	Enzyme
UDP-glucuronosyltransferase 1-6 *(by homology)*	P19224	UGT1A6	CHEMBL1743316		1 / 1	Enzyme
UDP-glucuronosyltransferase 1-1 *(by homology)*	P22309	UGT1A1	CHEMBL1287617		1 / 1	Enzyme
UDP-glucuronosyltransferase 1-3 *(by homology)*	P35503	UGT1A3			1 / 1	Enzyme
UDP-glucuronosyltransferase 1-5 *(by homology)*	P35504	UGT1A5			1 / 1	Enzyme
UDP-glucuronosyltransferase 1-7 *(by homology)*	Q9HAW7	UGT1A7	CHEMBL1743317		1 / 1	Enzyme
UDP-glucuronosyltransferase 1-10 *(by homology)*	Q9HAW8	UGT1A10	CHEMBL1743320		1 / 1	Enzyme
UDP-glucuronosyltransferase 1-8 *(by homology)*	Q9HAW9	UGT1A8	CHEMBL1743318		1 / 1	Enzyme
Estrogen receptor	P03372	ESR1	CHEMBL206		3 / 2	Transcription Factor
Estrogen receptor beta	Q92731	ESR2	CHEMBL242		3 / 2	Transcription Factor
Carbonic anhydrase 1	P00915	CA1	CHEMBL261		23 / 2	Enzyme

SwissTargetPrediction report:

Reference:
Gfeller D., Michielin O. & Zoete V.
Shaping the interaction landscape of bioactive molecules, *Bioinformatics* (2013) 29:3073-3079.

Query Molecule

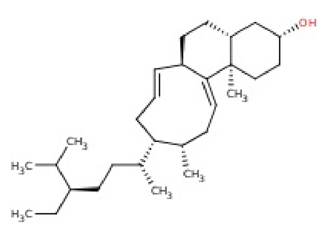

Frequency of Target Class

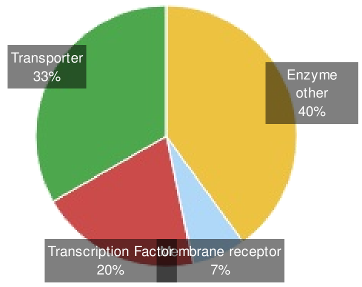

Target	Uniprot ID	Gene code	ChEMBL ID	Probability	# sim. cmpds (3D / 2D)	Target Class
Tyrosyl-DNA phosphodiesterase 1	Q9NUW8	TDP1	CHEMBL1075138		4 / 12	Enzyme
Cannabinoid receptor 1 *(by homology)*	P21554	CNR1	CHEMBL218		78 / 26	Membrane receptor
Androgen receptor	P10275	AR	CHEMBL1871		5 / 77	Transcription Factor
Sodium-dependent noradrenaline transporter	P23975	SLC6A2	CHEMBL222		17 / 6	Transporter
Sodium-dependent serotonin transporter	P31645	SLC6A4	CHEMBL228		20 / 6	Transporter
Sodium-dependent dopamine transporter *(by homology)*	Q01959	SLC6A3	CHEMBL238		17 / 6	Transporter
Steroid 17-alpha-hydroxylase/17,20 lyase	P05093	CYP17A1	CHEMBL3522		2 / 32	Enzyme
3-hydroxy-3-methylglutaryl-coenzyme A reductase	P04035	HMGCR	CHEMBL402		16 / 3	Enzyme
Oxysterols receptor LXR-beta	P55055	NR1H2	CHEMBL4093		17 / 23	Transcription Factor
Oxysterols receptor LXR-alpha	Q13133	NR1H3	CHEMBL2808		17 / 23	Transcription Factor
Cholinesterase *(by homology)*	P06276	BCHE	CHEMBL1914		12 / 1	Enzyme
Acetylcholinesterase	P22303	ACHE	CHEMBL220		12 / 1	Enzyme
Lanosterol 14-alpha demethylase *(by homology)*	Q16850	CYP51A1	CHEMBL3849		2 / 2	Enzyme
Sodium- and chloride-dependent glycine transporter 1 *(by homology)*	P48067	SLC6A9	CHEMBL2337		5 / 6	Transporter
Sodium- and chloride-dependent glycine transporter 2 *(by homology)*	Q9Y345	SLC6A5	CHEMBL3060		5 / 6	Transporter

SwissTargetPrediction report:

Reference:
Gfeller D., Michielin O. & Zoete V.
Shaping the interaction landscape of bioactive molecules, *Bioinformatics* (2013) 29:3073-3079.

Query Molecule

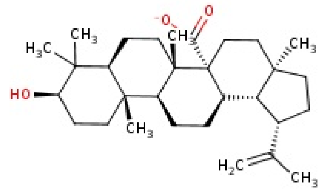

Frequency of Target Class

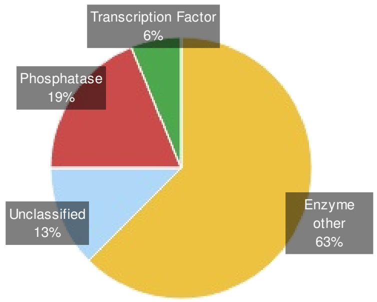

Target	Uniprot ID	Gene code	ChEMBL ID	Probability	# sim. cmpds (3D / 2D)	Target Class
DNA polymerase beta *(by homology)*	P06746	POLB	CHEMBL2392		5 / 12	Enzyme
Aldo-keto reductase family 1 member B10	O60218	AKR1B10	CHEMBL5983		3 / 8	Enzyme
Aldose reductase *(by homology)*	P15121	AKR1B1	CHEMBL1900		3 / 8	Enzyme
Complex	Q9UBE0/Q9UBT2	SAE1/UBA2	CHEMBL2095174		1 / 1	Unclassified /Enzyme
Aldo-keto reductase family 1 member B15 *(by homology)*	C9JRZ8	AKR1B15			3 / 8	Enzyme
Alcohol dehydrogenase [NADP(+)] *(by homology)*	P14550	AKR1A1	CHEMBL2246		3 / 8	Enzyme
1,5-anhydro-D-fructose reductase *(by homology)*	Q96JD6	AKR1E2			3 / 8	Enzyme
Microtubule-associated protein tau	P10636	MAPT	CHEMBL1293224		2 / 17	Unclassified
Corticosteroid 11-beta-dehydrogenase isozyme 1	P28845	HSD11B1	CHEMBL4235		5 / 14	Enzyme
Hydroxysteroid 11-beta-dehydrogenase 1-like protein *(by homology)*	Q7Z5J1	HSD11B1L			5 / 14	Enzyme
Tyrosyl-DNA phosphodiesterase 1	Q9NUW8	TDP1	CHEMBL1075138		1 / 17	Enzyme
Tyrosine-protein phosphatase non-receptor type 2	P17706	PTPN2	CHEMBL3807		10 / 32	Tyr Phosphatase
Tyrosine-protein phosphatase non-receptor type 1	P18031	PTPN1	CHEMBL335		10 / 32	Tyr Phosphatase
Bile acid receptor	Q96RI1	NR1H4	CHEMBL2047		0 / 13	Transcription Factor
M-phase inducer phosphatase 1	P30304	CDC25A	CHEMBL3775		5 / 19	Ser_Thr_Tyr Phosphatase

SwissTargetPrediction report:

Reference:
Gfeller D., Michielin O. & Zoete V.
Shaping the interaction landscape of bioactive molecules, *Bioinformatics* (2013) 29:3073-3079.

Query Molecule

Frequency of Target Class

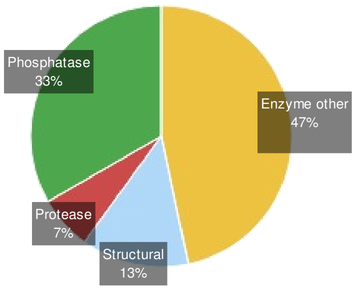

Target	Uniprot ID	Gene code	ChEMBL ID	Probability	# sim. cmpds (3D / 2D)	Target Class
Cocaine esterase	O00748	CES2	CHEMBL3180		0 / 6	Enzyme
Liver carboxylesterase 1	P23141	CES1	CHEMBL2265		0 / 6	Enzyme
Dynamin-1	Q05193	DNM1	CHEMBL4958		0 / 1	Structural
Tyrosyl-DNA phosphodiesterase 1	Q9NUW8	TDP1	CHEMBL1075138		0 / 1	Enzyme
N-acylethanolamine-hydrolyzing acid amidase subunit alpha *(by homology)*	Q02083	NAAA	CHEMBL4349		0 / 1	Enzyme
Carboxylesterase 5A *(by homology)*	Q6NT32	CES5A			0 / 6	Enzyme
Carboxylesterase 3 *(by homology)*	Q6UWW8	CES3			0 / 6	Enzyme
Dynamin-2 *(by homology)*	P50570	DNM2	CHEMBL5812		0 / 1	Enzyme
Dynamin-3 *(by homology)*	Q9UQ16	DNM3			0 / 1	Structural
Activation peptide fragment 1	P00734	F2	CHEMBL204		1 / 0	Serine Protease
Dual specificity protein phosphatase 3	P51452	DUSP3	CHEMBL2635		2 / 0	Ser_Thr_Tyr Phosphatase
M-phase inducer phosphatase 1 *(by homology)*	P30304	CDC25A	CHEMBL3775		5 / 0	Ser_Thr_Tyr Phosphatase
M-phase inducer phosphatase 2 *(by homology)*	P30305	CDC25B	CHEMBL4804		5 / 0	Ser_Thr_Tyr Phosphatase
Inactive dual specificity phosphatase 27 *(by homology)*	Q5VZP5	DUSP27			2 / 0	Ser_Thr_Tyr Phosphatase
Dual specificity phosphatase DUPD1 *(by homology)*	Q68J44	DUPD1			2 / 0	Ser_Thr_Tyr Phosphatase

SwissTargetPrediction report:

Reference:
Gfeller D., Michielin O. & Zoete V.
Shaping the interaction landscape of bioactive molecules, *Bioinformatics* (2013) 29:3073-3079.

Query Molecule

Frequency of Target Class

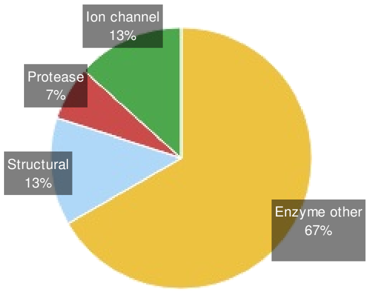

Target	Uniprot ID	Gene code	ChEMBL ID	Probability	# sim. cmpds (3D / 2D)	Target Class
Cocaine esterase	O00748	CES2	CHEMBL3180		0 / 6	Enzyme
Liver carboxylesterase 1	P23141	CES1	CHEMBL2265		0 / 6	Enzyme
Dynamin-1	Q05193	DNM1	CHEMBL4958		0 / 1	Structural
Tyrosyl-DNA phosphodiesterase 1	Q9NUW8	TDP1	CHEMBL1075138		0 / 1	Enzyme
N-acylethanolamine-hydrolyzing acid amidase subunit alpha *(by homology)*	Q02083	NAAA	CHEMBL4349		0 / 1	Enzyme
Carboxylesterase 5A *(by homology)*	Q6NT32	CES5A			0 / 6	Enzyme
Carboxylesterase 3 *(by homology)*	Q6UWW8	CES3			0 / 6	Enzyme
Dynamin-2 *(by homology)*	P50570	DNM2	CHEMBL5812		0 / 1	Enzyme
Dynamin-3 *(by homology)*	Q9UQ16	DNM3			0 / 1	Structural
Carbonic anhydrase 1	P00915	CA1	CHEMBL261		0 / 3	Enzyme
Carbonic anhydrase 2	P00918	CA2	CHEMBL205		0 / 3	Enzyme
Alpha-trypsin chain 1	P07477	PRSS1	CHEMBL209		0 / 1	Serine Protease
Carbonic anhydrase 4	P22748	CA4	CHEMBL3729		0 / 3	Enzyme
Glutamate receptor ionotropic, NMDA 1	Q05586	GRIN1	CHEMBL2015		0 / 1	Ion channel
Complex	Q05586/Q12879	GRIN1/GRIN2A	CHEMBL1907604		0 / 1	Ion channel

SwissTargetPrediction report:

Reference:
Gfeller D., Michielin O. & Zoete V.
Shaping the interaction landscape of bioactive molecules, *Bioinformatics* (2013) 29:3073-3079.

Query Molecule

Frequency of Target Class

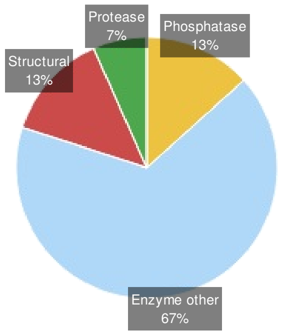

Target	Uniprot ID	Gene code	ChEMBL ID	Probability	# sim. cmpds (3D / 2D)	Target Class
M-phase inducer phosphatase 1 *(by homology)*	P30304	CDC25A	CHEMBL3775		1 / 0	Ser_Thr_Tyr Phosphatase
M-phase inducer phosphatase 2 *(by homology)*	P30305	CDC25B	CHEMBL4804		1 / 0	Ser_Thr_Tyr Phosphatase
Cocaine esterase	O00748	CES2	CHEMBL3180		0 / 6	Enzyme
Liver carboxylesterase 1	P23141	CES1	CHEMBL2265		0 / 6	Enzyme
Dynamin-1	Q05193	DNM1	CHEMBL4958		0 / 1	Structural
Tyrosyl-DNA phosphodiesterase 1	Q9NUW8	TDP1	CHEMBL1075138		0 / 1	Enzyme
N-acylethanolamine-hydrolyzing acid amidase subunit alpha *(by homology)*	Q02083	NAAA	CHEMBL4349		0 / 1	Enzyme
Carboxylesterase 5A *(by homology)*	Q6NT32	CES5A			0 / 6	Enzyme
Carboxylesterase 3 *(by homology)*	Q6UWW8	CES3			0 / 6	Enzyme
Dynamin-2 *(by homology)*	P50570	DNM2	CHEMBL5812		0 / 1	Enzyme
Dynamin-3 *(by homology)*	Q9UQ16	DNM3			0 / 1	Structural
Carbonic anhydrase 1	P00915	CA1	CHEMBL261		0 / 3	Enzyme
Carbonic anhydrase 2	P00918	CA2	CHEMBL205		0 / 3	Enzyme
Alpha-trypsin chain 1	P07477	PRSS1	CHEMBL209		0 / 1	Serine Protease
Carbonic anhydrase 4	P22748	CA4	CHEMBL3729		0 / 3	Enzyme

SwissTargetPrediction report:

Reference:
Gfeller D., Michielin O. & Zoete V.
Shaping the interaction landscape of bioactive molecules, *Bioinformatics* (2013) 29:3073-3079.

Query Molecule

Frequency of Target Class

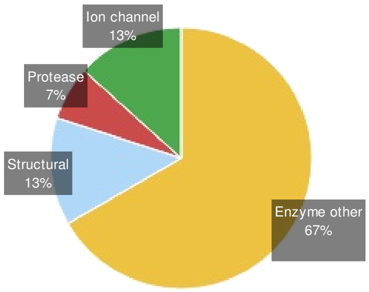

Target	Uniprot ID	Gene code	ChEMBL ID	Probability	# sim. cmpds (3D / 2D)	Target Class
Cocaine esterase	O00748	CES2	CHEMBL3180		0 / 6	Enzyme
Liver carboxylesterase 1	P23141	CES1	CHEMBL2265		0 / 6	Enzyme
Dynamin-1	Q05193	DNM1	CHEMBL4958		0 / 1	Structural
Tyrosyl-DNA phosphodiesterase 1	Q9NUW8	TDP1	CHEMBL1075138		0 / 1	Enzyme
N-acylethanolamine-hydrolyzing acid amidase subunit alpha *(by homology)*	Q02083	NAAA	CHEMBL4349		0 / 1	Enzyme
Carboxylesterase 5A *(by homology)*	Q6NT32	CES5A			0 / 6	Enzyme
Carboxylesterase 3 *(by homology)*	Q6UWW8	CES3			0 / 6	Enzyme
Dynamin-2 *(by homology)*	P50570	DNM2	CHEMBL5812		0 / 1	Enzyme
Dynamin-3 *(by homology)*	Q9UQ16	DNM3			0 / 1	Structural
Carbonic anhydrase 1	P00915	CA1	CHEMBL261		0 / 3	Enzyme
Carbonic anhydrase 2	P00918	CA2	CHEMBL205		0 / 3	Enzyme
Alpha-trypsin chain 1	P07477	PRSS1	CHEMBL209		0 / 1	Serine Protease
Carbonic anhydrase 4	P22748	CA4	CHEMBL3729		0 / 3	Enzyme
Glutamate receptor ionotropic, NMDA 1	Q05586	GRIN1	CHEMBL2015		0 / 1	Ion channel
Complex	Q05586/Q12879	GRIN1/GRIN2A	CHEMBL1907604		0 / 1	Ion channel

SwissTargetPrediction report:

Reference:
Gfeller D., Michielin O. & Zoete V.
Shaping the interaction landscape of bioactive molecules, *Bioinformatics* (2013) 29:3073-3079.

Query Molecule

Frequency of Target Class

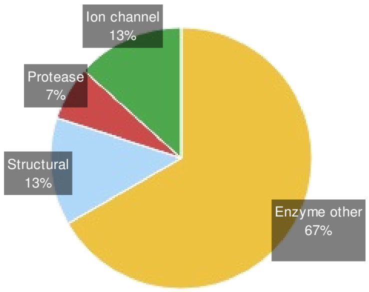

Target	Uniprot ID	Gene code	ChEMBL ID	Probability	# sim. cmpds (3D / 2D)	Target Class
Cocaine esterase	O00748	CES2	CHEMBL3180		0 / 6	Enzyme
Liver carboxylesterase 1	P23141	CES1	CHEMBL2265		0 / 6	Enzyme
Dynamin-1	Q05193	DNM1	CHEMBL4958		0 / 1	Structural
Tyrosyl-DNA phosphodiesterase 1	Q9NUW8	TDP1	CHEMBL1075138		0 / 1	Enzyme
N-acylethanolamine-hydrolyzing acid amidase subunit alpha *(by homology)*	Q02083	NAAA	CHEMBL4349		0 / 1	Enzyme
Carboxylesterase 5A *(by homology)*	Q6NT32	CES5A			0 / 6	Enzyme
Carboxylesterase 3 *(by homology)*	Q6UWW8	CES3			0 / 6	Enzyme
Dynamin-2 *(by homology)*	P50570	DNM2	CHEMBL5812		0 / 1	Enzyme
Dynamin-3 *(by homology)*	Q9UQ16	DNM3			0 / 1	Structural
Carbonic anhydrase 1	P00915	CA1	CHEMBL261		0 / 3	Enzyme
Carbonic anhydrase 2	P00918	CA2	CHEMBL205		0 / 3	Enzyme
Alpha-trypsin chain 1	P07477	PRSS1	CHEMBL209		0 / 1	Serine Protease
Carbonic anhydrase 4	P22748	CA4	CHEMBL3729		0 / 3	Enzyme
Glutamate receptor ionotropic, NMDA 1	Q05586	GRIN1	CHEMBL2015		0 / 1	Ion channel
Complex	Q05586/Q12879	GRIN1/GRIN2A	CHEMBL1907604		0 / 1	Ion channel

SwissTargetPrediction report:

Reference:
Gfeller D., Michielin O. & Zoete V.
Shaping the interaction landscape of bioactive molecules, *Bioinformatics* (2013) 29:3073-3079.

Query Molecule

Frequency of Target Class

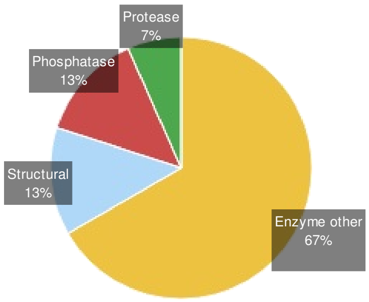

Target	Uniprot ID	Gene code	ChEMBL ID	Probability	# sim. cmpds (3D / 2D)	Target Class
Cocaine esterase	O00748	CES2	CHEMBL3180		0 / 6	Enzyme
Liver carboxylesterase 1	P23141	CES1	CHEMBL2265		0 / 6	Enzyme
Dynamin-1	Q05193	DNM1	CHEMBL4958		0 / 1	Structural
Tyrosyl-DNA phosphodiesterase 1	Q9NUW8	TDP1	CHEMBL1075138		0 / 1	Enzyme
N-acylethanolamine-hydrolyzing acid amidase subunit alpha *(by homology)*	Q02083	NAAA	CHEMBL4349		0 / 1	Enzyme
Carboxylesterase 5A *(by homology)*	Q6NT32	CES5A			0 / 6	Enzyme
Carboxylesterase 3 *(by homology)*	Q6UWW8	CES3			0 / 6	Enzyme
Dynamin-2 *(by homology)*	P50570	DNM2	CHEMBL5812		0 / 1	Enzyme
Dynamin-3 *(by homology)*	Q9UQ16	DNM3			0 / 1	Structural
M-phase inducer phosphatase 1 *(by homology)*	P30304	CDC25A	CHEMBL3775		1 / 0	Ser_Thr_Tyr Phosphatase
M-phase inducer phosphatase 2 *(by homology)*	P30305	CDC25B	CHEMBL4804		1 / 0	Ser_Thr_Tyr Phosphatase
Carbonic anhydrase 1	P00915	CA1	CHEMBL261		0 / 3	Enzyme
Carbonic anhydrase 2	P00918	CA2	CHEMBL205		0 / 3	Enzyme
Alpha-trypsin chain 1	P07477	PRSS1	CHEMBL209		0 / 1	Serine Protease
Carbonic anhydrase 4	P22748	CA4	CHEMBL3729		0 / 3	Enzyme

SwissTargetPrediction report:

Reference:
Gfeller D., Michielin O. & Zoete V.
Shaping the interaction landscape of bioactive molecules, *Bioinformatics* (2013) 29:3073-3079.

Query Molecule

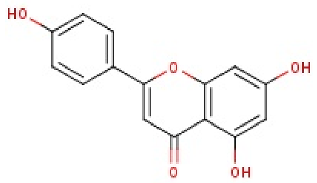

Frequency of Target Class

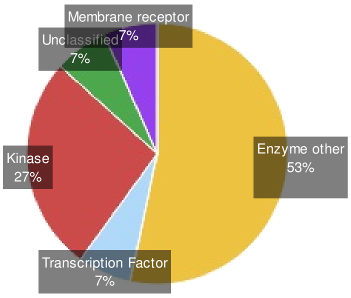

Target	Uniprot ID	Gene code	ChEMBL ID	Probability	# sim. cmpds (3D / 2D)	Target Class
Aldo-keto reductase family 1 member B10 *(by homology)*	O60218	AKR1B10	CHEMBL5983		21 / 67	Enzyme
Estrogen receptor	P03372	ESR1	CHEMBL206		165 / 60	Transcription Factor
Cytochrome P450 1A2 *(by homology)*	P05177	CYP1A2	CHEMBL3356		7 / 13	Enzyme
Cyclin-dependent kinase 1 *(by homology)*	P06493	CDK1	CHEMBL308		13 / 22	Ser_Thr Kinase
Microtubule-associated protein tau	P10636	MAPT	CHEMBL1293224		44 / 88	Unclassified
Cytochrome P450 19A1	P11511	CYP19A1	CHEMBL1978		10 / 19	Enzyme
Cyclin-dependent kinase 4 *(by homology)*	P11802	CDK4	CHEMBL331		13 / 22	Ser_Thr Kinase
Estradiol 17-beta-dehydrogenase 1	P14061	HSD17B1	CHEMBL3181		87 / 4	Enzyme
Aldose reductase *(by homology)*	P15121	AKR1B1	CHEMBL1900		21 / 67	Enzyme
Casein kinase II subunit alpha' *(by homology)*	P19784	CSNK2A2	CHEMBL4070		3 / 2	Ser_Thr Kinase
Amine oxidase [flavin-containing] A	P21397	MAOA	CHEMBL1951		8 / 45	Enzyme
Prostaglandin G/H synthase 1 *(by homology)*	P23219	PTGS1	CHEMBL221		15 / 15	Enzyme
Cyclin-dependent kinase 2 *(by homology)*	P24941	CDK2	CHEMBL301		13 / 22	Ser_Thr Kinase
Amine oxidase [flavin-containing] B *(by homology)*	P27338	MAOB	CHEMBL2039		8 / 45	Enzyme
Adenosine receptor A2a *(by homology)*	P29274	ADORA2A	CHEMBL251		5 / 13	Membrane receptor

SwissTargetPrediction report:

Reference:
Gfeller D., Michielin O. & Zoete V.
Shaping the interaction landscape of bioactive molecules, *Bioinformatics* (2013) 29:3073-3079.

Query Molecule

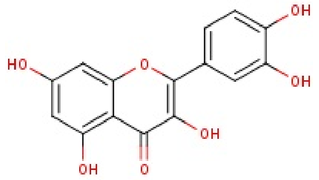

Frequency of Target Class

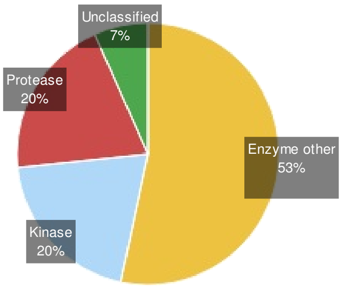

Target	Uniprot ID	Gene code	ChEMBL ID	Probability	# sim. cmpds (3D / 2D)	Target Class
Carbonic anhydrase 12	O43570	CA12	CHEMBL3242		3 / 5	Enzyme
Epidermal growth factor receptor	P00533	EGFR	CHEMBL203		6 / 28	Tyr Kinase
Carbonic anhydrase 1	P00915	CA1	CHEMBL261		10 / 7	Enzyme
Carbonic anhydrase 2	P00918	CA2	CHEMBL205		10 / 7	Enzyme
Phospholipase A2	P04054	PLA2G1B	CHEMBL4426		1 / 1	Enzyme
Receptor tyrosine-protein kinase erbB-2 *(by homology)*	P04626	ERBB2	CHEMBL1824		6 / 28	Tyr Kinase
Myeloperoxidase	P05164	MPO	CHEMBL2439		1 / 1	Enzyme
Cytochrome P450 1A2 *(by homology)*	P05177	CYP1A2	CHEMBL3356		8 / 13	Enzyme
Cyclin-dependent kinase 1	P06493	CDK1	CHEMBL308		3 / 27	Ser_Thr Kinase
Carbonic anhydrase 3	P07451	CA3	CHEMBL2885		10 / 7	Enzyme
Alpha-trypsin chain 1	P07477	PRSS1	CHEMBL209		1 / 2	Serine Protease
PEX	P08253	MMP2	CHEMBL333		5 / 2	Metallo Protease
Stromelysin-1	P08254	MMP3	CHEMBL283		3 / 2	Metallo Protease
Arachidonate 5-lipoxygenase	P09917	ALOX5	CHEMBL215		10 / 52	Enzyme
Microtubule-associated protein tau	P10636	MAPT	CHEMBL1293224		25 / 77	Unclassified

SwissTargetPrediction report:

Reference:
Gfeller D., Michielin O. & Zoete V.
Shaping the interaction landscape of bioactive molecules, *Bioinformatics* (2013) 29:3073-3079.

Query Molecule

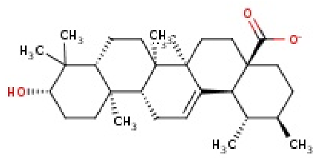

Frequency of Target Class

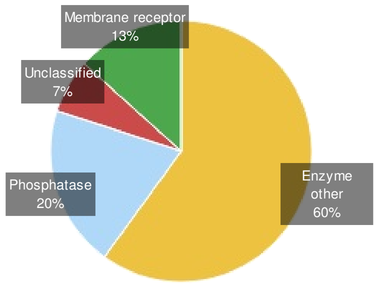

Target	Uniprot ID	Gene code	ChEMBL ID	Probability	# sim. cmpds (3D / 2D)	Target Class
DNA polymerase beta	P06746	POLB	CHEMBL2392		6 / 19	Enzyme
Tyrosine-protein phosphatase non-receptor type 2	P17706	PTPN2	CHEMBL3807		15 / 49	Tyr Phosphatase
Tyrosine-protein phosphatase non-receptor type 1	P18031	PTPN1	CHEMBL335		15 / 49	Tyr Phosphatase
Corticosteroid 11-beta-dehydrogenase isozyme 1	P28845	HSD11B1	CHEMBL4235		6 / 24	Enzyme
Hydroxysteroid 11-beta-dehydrogenase 1-like protein *(by homology)*	Q7Z5J1	HSD11B1L			6 / 24	Enzyme
Aldo-keto reductase family 1 member B10	O60218	AKR1B10	CHEMBL5983		4 / 7	Enzyme
Aldose reductase *(by homology)*	P15121	AKR1B1	CHEMBL1900		4 / 7	Enzyme
Aldo-keto reductase family 1 member B15 *(by homology)*	C9JRZ8	AKR1B15			4 / 7	Enzyme
Alcohol dehydrogenase [NADP(+)] *(by homology)*	P14550	AKR1A1	CHEMBL2246		4 / 7	Enzyme
1,5-anhydro-D-fructose reductase *(by homology)*	Q96JD6	AKR1E2			4 / 7	Enzyme
Phospholipase A2	P04054	PLA2G1B	CHEMBL4426		2 / 1	Enzyme
Microtubule-associated protein tau	P10636	MAPT	CHEMBL1293224		9 / 20	Unclassified
Receptor-type tyrosine-protein phosphatase F	P10586	PTPRF	CHEMBL3521		1 / 1	Membrane receptor
Low molecular weight phosphotyrosine protein phosphatase	P24666	ACP1	CHEMBL4903		1 / 1	Tyr Phosphatase
Receptor-type tyrosine-protein phosphatase delta *(by homology)*	P23468	PTPRD			1 / 1	Membrane receptor

SwissTargetPrediction report:

Reference:
Gfeller D., Michielin O. & Zoete V.
Shaping the interaction landscape of bioactive molecules, *Bioinformatics* (2013) 29:3073-3079.

Query Molecule

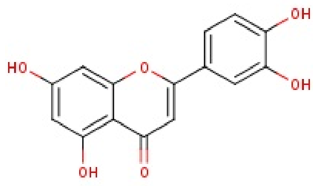

Frequency of Target Class

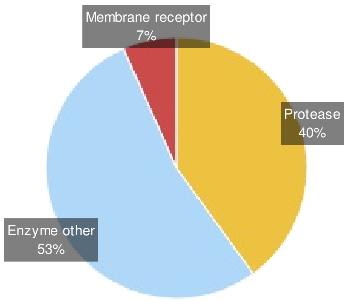

Target	Uniprot ID	Gene code	ChEMBL ID	Probability	# sim. cmpds (3D / 2D)	Target Class
22 kDa interstitial collagenase *(by homology)*	P03956	MMP1	CHEMBL332		4 / 2	Metallo Protease
Cytochrome P450 1A2 *(by homology)*	P05177	CYP1A2	CHEMBL3356		9 / 13	Enzyme
PEX	P08253	MMP2	CHEMBL333		4 / 2	Metallo Protease
Stromelysin-1 *(by homology)*	P08254	MMP3	CHEMBL283		4 / 2	Metallo Protease
67 kDa matrix metalloproteinase-9	P14780	MMP9	CHEMBL321		4 / 2	Metallo Protease
Aldose reductase *(by homology)*	P15121	AKR1B1	CHEMBL1900		19 / 68	Enzyme
Amine oxidase [flavin-containing] A	P21397	MAOA	CHEMBL1951		7 / 31	Enzyme
Amine oxidase [flavin-containing] B *(by homology)*	P27338	MAOB	CHEMBL2039		7 / 31	Enzyme
ADP-ribosyl cyclase 1	P28907	CD38	CHEMBL4660		1 / 2	Enzyme
Adenosine receptor A1 *(by homology)*	P30542	ADORA1	CHEMBL226		5 / 23	Membrane receptor
Macrophage metalloelastase	P39900	MMP12	CHEMBL4393		4 / 2	Metallo Protease
Collagenase 3 *(by homology)*	P45452	MMP13	CHEMBL280		4 / 2	Metallo Protease
Xanthine dehydrogenase/oxidase	P47989	XDH	CHEMBL1929		8 / 14	Enzyme
Lactoylglutathione lyase	Q04760	GLO1	CHEMBL2424		3 / 4	Enzyme
Lysine--tRNA ligase	Q15046	KARS	CHEMBL5575		4 / 3	Enzyme

SwissTargetPrediction report:

Reference:
Gfeller D., Michielin O. & Zoete V.
Shaping the interaction landscape of bioactive molecules, *Bioinformatics* (2013) 29:3073-3079.

Query Molecule

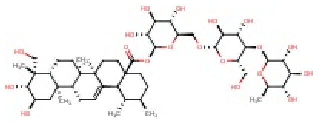

Frequency of Target Class

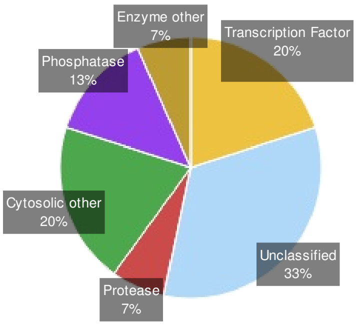

Target	Uniprot ID	Gene code	ChEMBL ID	Probability	# sim. cmpds (3D / 2D)	Target Class
Signal transducer and activator of transcription 3	P40763	STAT3	CHEMBL4026		1 / 4	Transcription Factor
Signal transducer and activator of transcription 1-alpha/beta *(by homology)*	P42224	STAT1	CHEMBL6101		1 / 4	Unclassified
Signal transducer and activator of transcription 2 *(by homology)*	P52630	STAT2			1 / 4	Transcription Factor
Signal transducer and activator of transcription 4 *(by homology)*	Q14765	STAT4			1 / 4	Transcription Factor
Activation peptide fragment 1	P00734	F2	CHEMBL204		1 / 10	Serine Protease
Bcl-2-like protein 1	Q07817	BCL2L1	CHEMBL4625		0 / 3	Cytosolic other
Apoptosis regulator Bcl-2 *(by homology)*	P10415	BCL2	CHEMBL4860		0 / 3	Cytosolic other
Bcl-2-like protein 2 *(by homology)*	Q92843	BCL2L2	CHEMBL4677		0 / 3	Cytosolic other
Microtubule-associated protein tau	P10636	MAPT	CHEMBL1293224		3 / 24	Unclassified
Tyrosine-protein phosphatase non-receptor type 2	P17706	PTPN2	CHEMBL3807		12 / 35	Tyr Phosphatase
Tyrosine-protein phosphatase non-receptor type 1	P18031	PTPN1	CHEMBL335		12 / 35	Tyr Phosphatase
3-hydroxy-3-methylglutaryl-coenzyme A reductase	P04035	HMGCR	CHEMBL402		2 / 83	Enzyme
Muscleblind-like protein 1	Q9NR56	MBNL1	CHEMBL1293317		2 / 4	Unclassified
Muscleblind-like protein 2 *(by homology)*	Q5VZF2	MBNL2			2 / 4	Unclassified
Muscleblind-like protein 3 *(by homology)*	Q9NUK0	MBNL3			2 / 4	Unclassified

SwissTargetPrediction report:

Reference:
Gfeller D., Michielin O. & Zoete V.
Shaping the interaction landscape of bioactive molecules, *Bioinformatics* (2013) 29:3073-3079.

Query Molecule

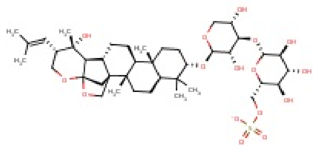

Frequency of Target Class

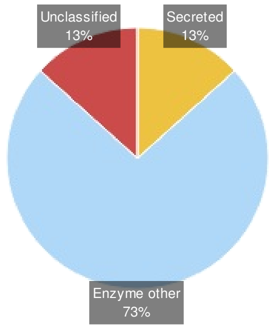

Target	Uniprot ID	Gene code	ChEMBL ID	Probability	# sim. cmpds (3D / 2D)	Target Class
Fibroblast growth factor 1	P05230	FGF1	CHEMBL2120		0 / 12	Secreted
Fibroblast growth factor 2	P09038	FGF2	CHEMBL3107		0 / 12	Secreted
Heparanase 8 kDa subunit	Q9Y251	HPSE	CHEMBL3921		0 / 7	Enzyme
Inactive heparanase-2 *(by homology)*	Q8WWQ2	HPSE2			0 / 7	Enzyme
Carbonic anhydrase 1	P00915	CA1	CHEMBL261		1 / 21	Enzyme
Carbonic anhydrase 2	P00918	CA2	CHEMBL205		1 / 21	Enzyme
Carbonic anhydrase 5A, mitochondrial *(by homology)*	P35218	CA5A	CHEMBL4789		1 / 21	Enzyme
Carbonic anhydrase 7 *(by homology)*	P43166	CA7	CHEMBL2326		1 / 21	Enzyme
Carbonic anhydrase 13 *(by homology)*	Q8N1Q1	CA13	CHEMBL3912		1 / 21	Enzyme
Carbonic anhydrase 5B, mitochondrial *(by homology)*	Q9Y2D0	CA5B	CHEMBL3969		1 / 21	Enzyme
Carbonic anhydrase 3 *(by homology)*	P07451	CA3	CHEMBL2885		1 / 21	Enzyme
Carbonic anhydrase 4	P22748	CA4	CHEMBL3729		1 / 1	Enzyme
Ras-related protein Rap-1A	P62834	RAP1A	CHEMBL1255139		0 / 1	Unclassified
cAMP-specific 3',5'-cyclic phosphodiesterase 4D	Q08499	PDE4D	CHEMBL288		0 / 1	Enzyme
Ras-related protein Rap-1b *(by homology)*	P61224	RAP1B			0 / 1	Unclassified

SwissTargetPrediction report:

Reference:
Gfeller D., Michielin O. & Zoete V.
Shaping the interaction landscape of bioactive molecules, *Bioinformatics* (2013) 29:3073-3079.

Query Molecule

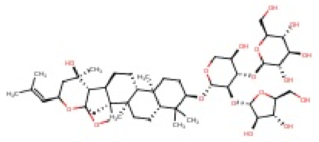

Frequency of Target Class

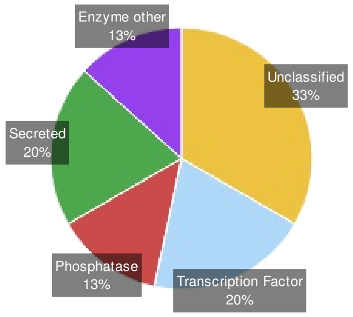

Target	Uniprot ID	Gene code	ChEMBL ID	Probability	# sim. cmpds (3D / 2D)	Target Class
Microtubule-associated protein tau	P10636	MAPT	CHEMBL1293224		1 / 12	Unclassified
Signal transducer and activator of transcription 3	P40763	STAT3	CHEMBL4026		2 / 4	Transcription Factor
Signal transducer and activator of transcription 1-alpha/beta *(by homology)*	P42224	STAT1	CHEMBL6101		2 / 4	Unclassified
Signal transducer and activator of transcription 2 *(by homology)*	P52630	STAT2			2 / 4	Transcription Factor
Signal transducer and activator of transcription 4 *(by homology)*	Q14765	STAT4			2 / 4	Transcription Factor
Muscleblind-like protein 1	Q9NR56	MBNL1	CHEMBL1293317		0 / 2	Unclassified
Muscleblind-like protein 2 *(by homology)*	Q5VZF2	MBNL2			0 / 2	Unclassified
Muscleblind-like protein 3 *(by homology)*	Q9NUK0	MBNL3			0 / 2	Unclassified
Tyrosine-protein phosphatase non-receptor type 1	P18031	PTPN1	CHEMBL335		4 / 6	Tyr Phosphatase
Tyrosine-protein phosphatase non-receptor type 2 *(by homology)*	P17706	PTPN2	CHEMBL3807		4 / 6	Tyr Phosphatase
Fibroblast growth factor 1	P05230	FGF1	CHEMBL2120		1 / 6	Secreted
Fibroblast growth factor 2	P09038	FGF2	CHEMBL3107		1 / 6	Secreted
Vascular endothelial growth factor A	P15692	VEGFA	CHEMBL1783		1 / 2	Secreted
Heparanase 8 kDa subunit	Q9Y251	HPSE	CHEMBL3921		1 / 3	Enzyme
Inactive heparanase-2 *(by homology)*	Q8WWQ2	HPSE2			1 / 3	Enzyme
