# Supplementary figures and images for: Bacopa monnieri and Their Bioactive Compounds Inferred Multi-Target Treatment Strategy for Neurological Diseases: A Cheminformatics and System Pharmacology Approach
Source: Biomolecules. 2020 Apr 2;10(4):536. doi: 10.3390/biom10040536 (PMC7225932; doi:10.3390/biom10040536)

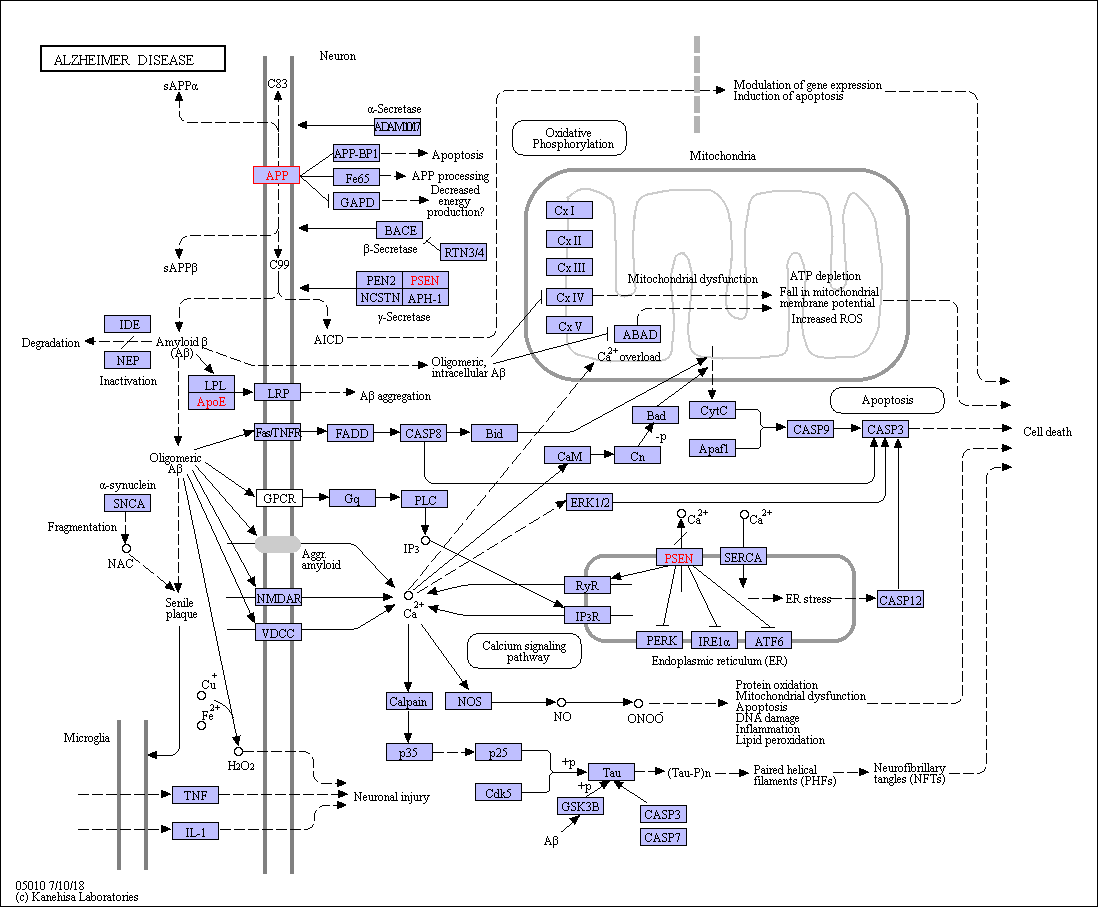

Supplement: Supplementary file 1 [file biomolecules-10-00536-s001.zip › Supplementary Figure 1 alzheimer's disease pathway.png]

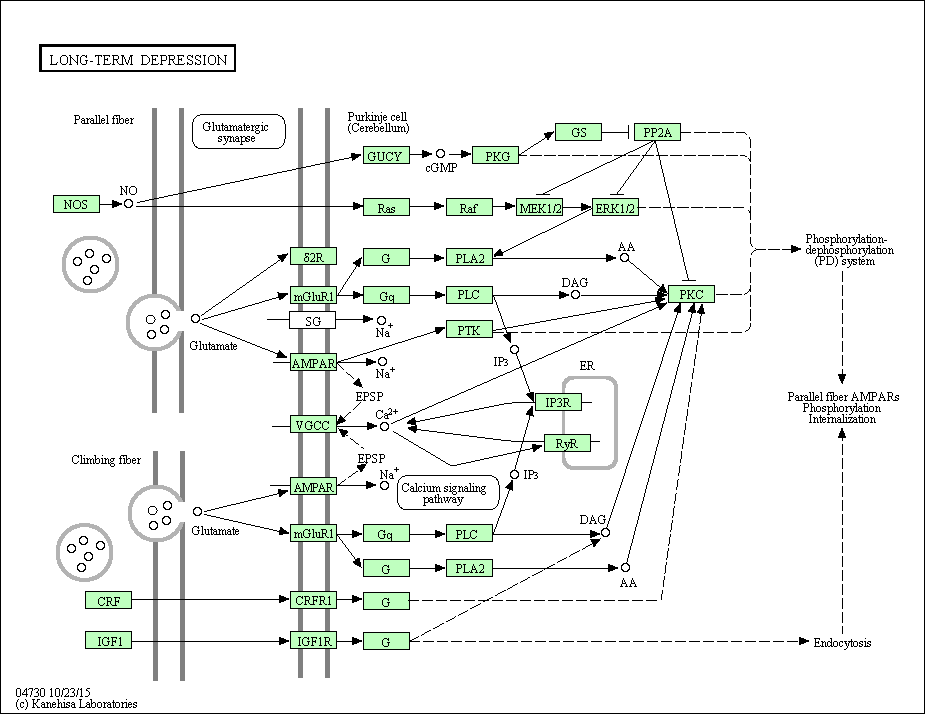

Supplement: Supplementary file 1 [file biomolecules-10-00536-s001.zip › Supplementary Figure 2 SCA.png]
