# Supplementary material for: Bacopa monnieri and Their Bioactive Compounds Inferred Multi-Target Treatment Strategy for Neurological Diseases: A Cheminformatics and System Pharmacology Approach
Source: Biomolecules. 2020 Apr 2;10(4):536. doi: 10.3390/biom10040536 (PMC7225932; doi:10.3390/biom10040536)
Supplement: Supplementary file 1 [file biomolecules-10-00536-s001.zip › Table S1 Canonical SMILES.doc]

**Table S1.** *Compound and canonical SMILES*

| *Compound* | *Canonical SMILES* |
| --- | --- |
| *Nt* | *CN1CCCC1C2=CN=CC=C2* |
| *D-Mn* | *C(C(C(C(C(CO)O)O)O)O)O* |
| *BCS A* | *CC(=CCCC(C)(C1C2CCC3C(C2(CC1=O)C)(CCC4C3(CCC(C4(C)C)OC5C(C(C(C(O5)CO)OC6C(C(C(CO6)O)O)O)O)O)CO)C)O)C* |
| *BCSN A* | *CC(=CC1CC(C2C3CCC4C5(CCC(C(C5CCC4(C36CC2(O1)OC6)C)(C)C)OC7C(C(C(CO7)O)O)O)C)(C)OC8C(C(C(CO8)O)O)O)C* |
| *BCSN B* | *CC(=CC1COC23CC4(CO2)C(C3C1(C)O)CCC5C4(CCC6C5(CCC(C6(C)C)OC7C(C(C(CO7)O)O)OC8C(C(C(O8)CO)O)O)C)C)C* |
| *BCSN C* | *CC(=CC1COC23CC4(CO2)C(C3C1(C)O)CCC5C4(CCC6C5(CCC(C6(C)C)OC7C(C(C(CO7)O)OC8C(C(C(C(O8)CO)O)O)O)OC9C(C(C(O9)CO)O)O)C)C)C* |
| *BCSN D* | *CC(=CC1COC23CC4(CO2)C(C3C1(C)O)CCC5C4(CCC6C5(CCC(C6(C)C)OC7C(C(C(C(O7)CO)O)O)OC8C(C(C(O8)CO)O)O)C)C)C* |
| *BCSN E* | *CC(=CC1CC(C2C3CCC4C5(CCC(C(C5CCC4(C36CC2(O1)OC6)C)(C)C)OC7C(C(C(CO7)O)OC8C(C(C(C(O8)CO)O)O)O)OC9C(C(C(O9)CO)O)O)C)(C)OC1C(C(C(CO1)O)O)O)C* |
| *BCSN F* | *CC(=CC1CC(C2C3CCC4C5(CCC(C(C5CCC4(C36CC2(O1)OC6)C)(C)C)OC7C(C(C(C(O7)CO)O)OC8C(C(C(C(O8)CO)O)O)O)OC9C(C(C(O9)CO)O)O)C)(C)OC1C(C(C(CO1)O)O)O)C* |
| *BCSN G* | *CC(=CC1CC(C2C3CCC4C5(CCC(C(C5CCC4(C36CC2(O1)OC6)C)(C)C)OC7C(C(C(CO7)O)O)OC8C(C(C(O8)CO)O)O)C)(C)O)C* |
| *BPS I* | *CC(=CC1COC23CC4(CO2)C(C3C1(C)O)CCC5C4(CCC6C5(CCC(C6(C)C)OC7C(C(C(CO7)O)OC8C(C(C(C(O8)COS(=O)(=O)O)O)O)O)OC9C(C(C(O9)CO)O)O)C)C)C* |
| *BPS II* | *CC(=CC1COC23CC4(CO2)C(C3C1(C)O)CCC5C4(CCC6C5(CCC(C6(C)C)OC7C(C(C(C(O7)CO)O)OC8C(C(C(C(O8)CO)O)O)O)OC9C(C(C(O9)CO)O)O)C)C)C* |
| *BPS III* | *CC(=CC1COC23CC4(CO2)C(C3C1(C)O)CCC5C4(CCC6C5(CCC(C6(C)C)OC7C(C(C(CO7)O)OC8C(C(C(C(O8)COS(=O)(=O)O)O)O)O)O)C)C)C* |
| *BPS IV* | *CC(=CC1CC(C2C3CCC4C5(CCC(C(C5CCC4(C36CC2(O1)OC6)C)(C)C)OC7C(C(C(CO7)O)OC8C(C(C(C(O8)CO)O)O)O)O)C)(C)O)C* |
| *BPS V* | *CC(=CC1CC(C2C3CCC4C5(CCC(C(C5CCC4(C36CC2(O1)OC6)C)(C)C)OC7C(C(C(CO7)O)OC8C(C(C(C(O8)CO)O)O)O)O)C)(C)O)C* |
| *BPS VIII* | *CC(=CC1CC(C2C3CCC4C5(CCC(C(C5CCC4(C36CC2(O1)OC6)C)(C)C)OC7C(C(C(C(O7)CO)O)OC8C(C(C(C(O8)CO)O)O)O)OC9C(C(C(O9)CO)O)O)C)(C)OC1C(C(C(CO1)O)O)O)C* |
| *BPS XII* | *CC(=CC1COC23CC4(CO2)C(C3C1(C)OC5C(C(C(CO5)O)O)O)CCC6C4(CCC7C6(CCC(C7(C)C)OC8C(C(C(C(O8)CO)O)OC9C(C(C(C(O9)CO)O)O)O)OC1C(C(C(O1)CO)O)O)C)C)C* |
| *PTS B* | *C1=CC(=C(C=C1CCOC2C(C(C(C(O2)CO)O)O)OC(=O)C=CC3=CC(=C(C=C3)O)O)O)O* |
| *BTA* | *CC(=C)C1CCC2(C1C3CCC4C5(CCC(C(C5CCC4(C3(CC2)C)C)(C)C)O)C)C(=O)O* |
| *CCB A* | *CC(=O)OC(C)(C)C=CC(=O)C(C)(C1C(CC2(C1(CC(=O)C3(C2CC=C4C3CC(C(=O)C4(C)C)O)CO)C)C)O)O* |
| *CCB B* | *CC(=O)OC(C)(C)C=CC(=O)C(C)(C1C(CC2(C1(CC(=O)C3(C2CC=C4C3CC(C(=O)C4(C)C)O)C)C)C)O)O* |
| *CCB C* | *CC(=O)OC(C)(C)C=CC(=O)C(C)(C1C(CC2(C1(CC(=O)C3(C2CC=C4C3CCC(C4(C)C)O)CO)C)C)O)O* |
| *CCB D* | *CC1(C2=CCC3C4(CC(C(C4(CC(=O)C3(C2CC(C1=O)O)C)C)C(C)(C(=O)C=CC(C)(C)O)O)O)C)C* |
| *CCB E* | *CC(=O)OC(C)(C)C=CC(=O)C(C)(C1C(CC2(C1(CC(=O)C3(C2CC=C4C3C=C(C(=O)C4(C)C)O)C)C)C)O)O* |
| *STRA* | *CCCCCCCCCCCCCCCCCC(=O)O* |
| *RSV* | *C1C(C(C(C(O1)OCC2C(C(C(C(O2)OCC=CC3=CC=CC=C3)O)O)O)O)O)O* |
| *3,4DMCA* | *COC1=C(C=C(C=C1)C=CC(=O)O)OC* |
| *ASBA* | *C(C(C1C(=C(C(=O)O1)O)O)O)O* |
| *ASTA* | *CC1CCC2(CCC3(C(=CCC4C3(CCC5C4(CC(C(C5(C)CO)O)O)C)C)C2C1C)C)C(=O)O* |
| *BMA* | *CC1CCC2(CCC3(C(=CCC4C3(CC(C5C4(CC(C(C5(C)CO)O)O)C)O)C)C2C1C)C)C(=O)O* |
| *WG* | *COC1=C(C=C(C2=C1OC(=CC2=O)C3=CC=CC=C3)O)O* |
| *OX* | *COC1=C(C=C(C2=C1OC(=CC2=O)C3=CC=CC=C3)O)OC4C(C(C(C(O4)C(=O)O)O)O)O* |
| *LLD* | *CC1(CC(CC2(C1=CC(=O)O2)C)O)C* |
| *SGMS* | *CCC(C=CC(C)C1CCC2C1(CCC3C2CC=C4C3(CCC(C4)O)C)C)C(C)C* |
| *β-SS* | *CCC(CCC(C)C1CCC2C1(CCC3C2CC=C4C3(CCC(C4)O)C)C)C(C)C* |
| *EBL* | *CC(=CC=CC(=CC1CCC2C3(CCC(C(C3CCC2(C14CC(=O)OC4)C)(C)C)O)C)C)C* |
| *SGMSL* | *CCC(CCC(C)C1CCC2C1(CCC3C2CCC4C3(CCC(C4)O)C)C)C(C)C* |
| *BCSt* | *CCC(CCC(C)C1CC=CC2CCC3CC(CCC3(C2=CCC1C)C)O)C(C)C* |
| *BCSn* | *CC(=C)C1CCC2(C1C3CCC4C5(CCC(C(C5CCC4(C3(CC2)C(=O)O)C)(C)C)O)C)C* |
| *HCS* | *CCCCCCCCCCCCCCCCCCCCCCCCCCC* |
| *OCS* | *CCCCCCCCCCCCCCCCCCCCCCCCCCCC* |
| *NCS* | *CCCCCCCCCCCCCCCCCCCCCCCCCCCCC* |
| *TC* | *CCCCCCCCCCCCCCCCCCCCCCCCCCCCCC* |
| *HTA* | *CCCCCCCCCCCCCCCCCCCCCCCCCCCCCCC* |
| *DOT* | *CCCCCCCCCCCCCCCCCCCCCCCCCCCCCCCC* |
| *AG* | *C1=CC(=CC=C1C2=CC(=O)C3=C(C=C(C=C3O2)O)O)O* |
| *QR* | *C1=CC(=C(C=C1C2=C(C(=O)C3=C(C=C(C=C3O2)O)O)O)O)O* |
| *USA* | *CC1CCC2(CCC3(C(=CCC4C3(CCC5C4(CCC(C5(C)C)O)C)C)C2C1C)C)C(=O)O* |
| *LT* | *C1=CC(=C(C=C1C2=CC(=O)C3=C(C=C(C=C3O2)O)O)O)O* |
| *ASTS* | *CC1CCC2(CCC3(C(=CCC4C3(CCC5C4(CC(C(C5(C)CO)O)O)C)C)C2C1C)C)C(=O)OC6C(C(C(C(O6)COC7C(C(C(C(O7)CO)OC8C(C(C(C(O8)C)O)O)O)O)O)O)O)O* |
| *BPS VI* | *CC(=CC1COC23CC4(CO2)C(C3C1(C)O)CCC5C4(CCC6C5(CCC(C6(C)C)OC7C(C(C(CO7)O)OC8C(C(C(C(O8)COS(=O)(=O)O)O)O)O)O)C)C)C* |
| *BPS VII* | *CC(=CC1CC(C2C3CCC4C5(CCC(C(C5CCC4(C36CC2(O1)OC6)C)(C)C)OC7C(C(C(CO7)O)OC8C(C(C(C(O8)CO)O)O)O)OC9C(C(C(O9)CO)O)O)C)(C)O)C* |
