# Supplementary material for: Bacopa monnieri and Their Bioactive Compounds Inferred Multi-Target Treatment Strategy for Neurological Diseases: A Cheminformatics and System Pharmacology Approach
Source: Biomolecules. 2020 Apr 2;10(4):536. doi: 10.3390/biom10040536 (PMC7225932; doi:10.3390/biom10040536)
Supplement: Supplementary file 1 [file biomolecules-10-00536-s001.zip › Table S2 Compound and targets.doc]

**Table S2.** *List of compounds and human targets*

| *S. No* | *Compounds* | *Target* | *Total No. of Targets* |
| --- | --- | --- | --- |
| *1* | *Nt* | *CHRNB1/CHRND/CHRNA1/CHRNG, CHRNB2/CHRNA4, CHRNB4/CHRNA3, CHRNB4/CHRNA4, CHRNB4/CHRNA2, CHRNA3, CHRNA3/CHRNB2, CHRNA7, CHRNA4, CHRNA2/CHRNB2, CHRFAM7A, CHRNA2, CHRNB2, CHRNB4, CHRNA10* | *24* |
| *2* | *D-Mn* | *TDP1, GBA, CA1, CA2, CA3, CA5A, CA7, CA13, CA5B, FUCA1, FUCA2, NR1H2, NR1H3, MGAM, SI* | *15* |
| *3* | *BCS A* | *STAT3, STAT1, STAT2, STAT4, MAPT, FLT1, KDR, FLT4, ADRA2A, ADRA2B, ADRA2C, CYP2D6, CYP2J2, PTAFR, HSD11B1* | *15* |
| *4* | *BCSN A* | *MBNL1, MBNL2, MBNL3, STAT3, STAT1, STAT2, STAT4, PTPN2, PTPN1, MAPT, PRKCG, PRKCB, PRKCA, PRKCQ, PRKCD* | *15* |
| *5* | *BCSN B* | *STAT3, STAT1, STAT2, STAT4, FGF1, FGF2, VEGFA, HPSE, HPSE2, CHRM5, CHRM1, CHRM3, CHRM2, CHRM4, MBNL1* | *15* |
| *6* | *BCSN C* | *PTPN2, PTPN1, STAT3, STAT1, STAT2, STAT4, FGF1, FGF2, VEGFA, HPSE, HPSE2, MBNL1, MBNL2, MBNL3, CHRM5* | *15* |
| *7* | *BCSN D* | *PTPN1, PTPN2, MBNL1, MBNL2, MBNL3, MAPT, PPP1CC, PPP1CA, PPP1CB, STAT3, STAT1, STAT2, STAT4, DRD2, HTR2A* | *15* |
| *8* | *BCSN E* | *MBNL1, MBNL2, MBNL3, PTPN1, PTPN2, STAT3, STAT1, STAT2, STAT4, MAPT, OPRM1, OPRD1, OPRK1, OPRL1, FGF1* | *15* |
| *9* | *BCSN F* | *MBNL1, MBNL2, MBNL3, PTPN1, PTPN2, STAT3, STAT1, STAT2, STAT4, MAPT, OPRM1, OPRD1, OPRK1, OPRL1, FGF1* | *15* |
| *10* | *BCSN G* | *MAPT, STAT3, STAT1, STAT2, STAT4, MBNL1, MBNL2, MBNL3, PTPN1, PTPN2, FLT1, KDR, FLT4, PRKCG, PRKCB* | *15* |
| *11* | *BPS I* | *FGF1, FGF2, HPSE, HPSE2, CA1, CA2, CA5A, CA7, CA13, CA5B, CA3, CA4, CA12, CA9, CA14* | *15* |
| *12* | *BPS II* | *PTPN1, PTPN2, STAT3, STAT1, STAT2, STAT4, FGF1, FGF2, VEGFA, HPSE, HPSE2, MBNL1, MBNL2, MBNL3, CHRM5* | *15* |
| *13* | *BPS III* | *FGF1, FGF2, HPSE, HPSE2, CA1, CA2, CA5A, CA7, CA13, CA5B, CA3, CA4, RAP1A, PDE4D, RAP1B* | *15* |
| *14* | *BPS IV* | *MBNL1, MBNL2, MBNL3, STAT3, STAT1, STAT2, STAT4, FLT1, KDR, FLT4, MAPT, FGF1, FGF2, VEGFA, HPSE* | *15* |
| *15* | *BPS V* | *STAT3, STAT1, STAT2, STAT4, BCL2L1, BCL2, BCL2L2, MBNL1, MBNL2, MBNL3, FLT1, KDR, FLT4, FGF1, FGF2* | *15* |
| *16* | *BPS VIII* | *MBNL1, MBNL2, MBNL3, PTPN1, PTPN2, STAT3, STAT1, STAT2, STAT4, MAPT, OPRM1, OPRD1, OPRK1, OPRL1, FGF1* | *15* |
| *17* | *BPS XII* | *STAT3, STAT1, STAT2, STAT4, PTPN1, PTPN2, FGF1, FGF2, VEGFA, HPSE, HPSE2, MBNL1, MBNL2, MBNL3, CHRM5* | *15* |
| *18* | *PTS B* | *PRKCG, PRKCB, PRKCA, PRKCQ, PRKCD, MMP2, MMP9, MMP1, MMP3, MMP12, MMP13, MMP10, MMP27, AKR1B10, AKR1B1* | *15* |
| *19* | *BTA* | *AKR1B10, POLB, AKR1B1, AKR1B15, AKR1A1, AKR1E2, SAE1/UBA2, MAPT, HSD11B1, HSD11B1L, NR1H4, CDC25A, CDC25B, TOP2A, TOP2B* | *16* |
| *20* | *CCB A* | *ITGAL, STAT3, STAT1, STAT2, STAT4, POLA1, CRYZ, MAPT, HMGCR, TTL, MMP10, MMP3, MMP8, MMP12, MMP13* | *15* |
| *21* | *CCB B* | *ITGAL, STAT3, STAT1, STAT4, STAT2, MAPT, POLA1, NOS1, NOS2, NOS3, MMP12, MMP3, MMP10, MMP8, MMP12* | *15* |
| *22* | *CCB C* | *ITGAL, STAT3, STAT1, STAT4, STAT2, MAPT, POLA1, CRYZ, HSD11B1, HSD11B1L, NOS2, NOS3, NOS1, HMGCR, BCL2* | *15* |
| *23* | *CCB D* | *ITGAL, STAT3, STAT1, STAT2, STAT4, PTGS1, PTGS2, MMP1, MMP3, MMP8, MMP12, MMP13, MMP10, MMP27, NR3C1* | *15* |
| *24* | *CCB E* | *ITGAL, STAT3, STAT1, STAT4, STAT2, CRYZ, POLA1, ADORA1, NR1I2, PTGS1, PTGS2, CYP19A1, MMP1, MMP3, MMP8* | *15* |
| *25* | *STRA* | *FABP3, FABP4, FABP5, FABP12, FABP7, PMP2, FABP9, FABP2, TDP1, CDC25A, CDC25B, HSD11B1, HSD11B1L, PTGER2, MAPT* | *15* |
| *26* | *RSV* | *MMP1, MMP12, MMP13, MMP3, MMP10, MMP27, MMP2, MMP9, PRKCG, PRKCB, PRKCA, PRKCQ, PRKCD, FDFT1, MMP20* | *15* |
| *27* | *3,4DMCA* | *CA1, CA2, CA3, CA5A, CA7, CA13, CA5B, CA12, CA9, CA14, DYRK1A, MAOA, MAOB, PTGS1, PTGS2* | *15* |
| *28* | *ASBA* | *TDP1, TTL, PRKCA, PRKCD, PRKCQ, PRKCG, PRKCB, DUSP3, DUSP27, DUPD1, DUSP13, DUSP26, RPS6KA5, RPS6KA4, RPS6KA3* | *15* |
| *29* | *ASTA* | *AKR1B10, AKR1B1, AKR1B15, AKR1A1, AKR1E2, PTPN2, PTPN1, HSD11B1, HSD11B1L, TDP1, MAPT, POLB, PTPRF, ACP1, PTPRD* | *15* |
| *30* | *BMA* | *AKR1B10, AKR1B1, AKR1B15, AKR1A1, AKR1E2, MAPT, TDP1, PTPN2, PTPN1, HSD11B1, HSD11B1L, PLA2G1B, POLB, PTPRF, ACP1* | *15* |
| *31* | *WG* | *PTGS1, NOS2, PTGS2, NOS3, NOS1, AKR1B1, AKR1B15, AKR1B10, IKBKB, CHUK, DYRK1A, MAPT, ABCG2, TDP1, ALOX5* | *15* |
| *32* | *OX* | *ADORA1, AKR1B1, AKR1B15, AKR1B10, TDP1, DYRK1A, MBNL1, MBNL2, MBNL3, XDH, AOX1, KDM4A, KDM4B, KDM4C, PTGS2* | *15* |
| *33* | *LLD* | *TDP1, MAPT, CRYZ, RPS6KA5, RPS6KA4, RPS6KA3, RPS6KA2, RPS6KA1, RPS6KA6, PTGS1, PTGS2, PTAFR, CDC25A, CDC25B, CYP19A1* | *15* |
| *34* | *SGMS* | *AR, TDP1, CYP19A1, HMGCR, CYP51A1, NR1H2, NR1H3, LDLR, VLDLR, LRP8, CYP17A1, ESR1, ESR2, SLC6A2, SLC6A315* | *15* |
| *35* | *β-SS* | *TDP1, AR, HMGCR, CYP51A1, NR1H2, NR1H3, CYP19A1, CYP17A1, LDLR, VLDLR, LRP8, ESR1, ESR2, SLC6A2, SLC6A4* | *15* |
| *36* | *EBL* | *DRD2, DRD3, HMGCR, HTR1A, HTR1B, PTPN2, PTPN1, NR3C1, NR3C2, AR, SLC6A2, TACR1, SLC6A4, SLC6A3, TACR3* | *15* |
| *37* | *SGMSL* | *AR, CDC25A, CDC25B, UGT1A4, UGT1A9, UGT1A6, UGT1A1, UGT1A3, UGT1A5, UGT1A7, UGT1A10, UGT1A8, ESR1, ESR2, CA1* | *15* |
| *38* | *BCSt* | *TDP1, CNR1, AR, SLC6A2, SLC6A4, SLC6A3, CYP17A1, HMGCR, NR1H2, NR1H3, BCHE, ACHE, CYP51A1, SLC6A9, SLC6A5* | *15* |
| *39* | *BCSn* | *POLB, AKR1B10, AKR1B1, SAE1/UBA2, AKR1B15, AKR1A1, AKR1E2, MAPT, HSD11B1, HSD11B1L, TDP1, PTPN2, PTPN1, NR1H4, CDC25A* | *16* |
| *40* | *HCS* | *CES2, CES1, DNM1, TDP1, NAAA, CES5A, CES3, DNM2, DNM3, F2, DUSP3, CDC25A, CDC25B, DUSP27, DUPD1* | *15* |
| *41* | *OCS* | *CES2, CES1, DNM1, TDP1, NAAA, CES5A, CES3, DNM2, DNM3, CA1, CA2, PRSS1, CA4, GRIN1, GRIN1/GRIN2A* | *16* |
| *42* | *NCS* | *CDC25A, CDC25B, CES2, CES1, DNM1, TDP1, NAAA, CES5A, CES3, DNM2, DNM3, CA1, CA2, PRSS1, CA4* | *15* |
| *43* | *TC* | *CES2, CES1, DNM1, TDP1, NAAA, CES5A, CES3, DNM2, DNM3, CA1, CA2, PRSS1, CA4, GRIN1, GRIN1/GRIN2A* | *16* |
| *44* | *HTA* | *CES2, CES1, DNM1, TDP1, NAAA, CES5A, CES3, DNM2, DNM3, CA1, CA2, PRSS1, CA4, GRIN1, GRIN1/GRIN2A* | *16* |
| *45* | *DOT* | *CES2, CES1, DNM1, TDP1, NAAA, CES5A, CES3, DNM2, DNM3, CDC25A, CDC25B, CA1, CA2, PRSS1, CA4* | *15* |
| *46* | *AG* | *AKR1B10, ESR1, CYP1A2, CDK1, MAPT, CYP19A1, CDK4, HSD17B1, AKR1B1, CSNK2A2, MAOA, PTGS1, CDK2, MAOB, ADORA2A* | *15* |
| *47* | *QR* | *CA12, EGFR, CA12, CA2, PLA2G1B, ERBB2, MPO, CYP1A2, CDK1, CA3, PRSS1, MMP2, MMP3, ALOX5, MAPT* | *15* |
| *48* | *USA* | *POLB, PTPN2, PTPN1, HSD11B1, HSD11B1L, AKR1B10, AKR1B1, AKR1B15, AKR1A1, AKR1E2, PLA2G1B, MAPT, PTPRF, ACP1, PTPRD* | *15* |
| *49* | *LT* | *MMP1, CYP1A2, MMP2, MMP3, MMP9, AKR1B10, MAOA, MAOB, CD38, ADORA1, MMP12, MMP13, XDH, GLO1, KARS* | *15* |
| *50* | *ASTS* | *STAT3, STAT1, STAT2, STAT4, F2, BCL2L1, BCL2, BCL2L2, MAPT, PTPN2, PTPN1, HMGCR, MBNL1, MBNL2, MBNL3* | *15* |
| *51* | *BPS VI* | *FGF1, FGF2, HPSE, HPSE2, CA1, CA2, CA5A, CA7, CA13, CA5B, CA3, CA4, RAP1A, PDE4D, RAP1B* | *15* |
| *52* | *BPS VII* | *MAPT, STAT3, STAT1, STAT2, STAT4, MBNL1, MBNL2, MBNL3, PTPN1, PTPN2, FGF1, FGF2, VEGFA, HPSE, HPSE* | *15* |
